# Use of Alignment-Free Phylogenetics for Rapid Genome Sequence-Based Typing of Helicobacter pylori Virulence Markers and Antibiotic Susceptibility

**DOI:** 10.1128/JCM.01357-15

**Published:** 2015-08-18

**Authors:** Arnoud H. M. van Vliet, Johannes G. Kusters

**Affiliations:** a Institute of Food Research, Gut Health and Food Safety Programme, Norwich Research Park, Norwich, United Kingdom; b University Medical Center Utrecht, Department of Medical Microbiology, Utrecht, The Netherlands

## Abstract

Whole-genome sequencing is becoming a leading technology in the typing and epidemiology of microbial pathogens, but the increase in genomic information necessitates significant investment in bioinformatic resources and expertise, and currently used methodologies struggle with genetically heterogeneous bacteria such as the human gastric pathogen Helicobacter pylori. Here we demonstrate that the alignment-free analysis method feature frequency profiling (FFP) can be used to rapidly construct phylogenetic trees of draft bacterial genome sequences on a standard desktop computer and that coupling with *in silico* genotyping methods gives useful information for comparative and clinical genomic and molecular epidemiology applications. FFP-based phylogenetic trees of seven gastric Helicobacter species matched those obtained by analysis of 16S rRNA genes and ribosomal proteins, and FFP- and core genome single nucleotide polymorphism-based analysis of 63 H. pylori genomes again showed comparable phylogenetic clustering, consistent with genomotypes assigned by using multilocus sequence typing (MLST). Analysis of 377 H. pylori genomes highlighted the conservation of genomotypes and linkage with phylogeographic characteristics and predicted the presence of an incomplete or nonfunctional *cag* pathogenicity island in 18/276 genomes. *In silico* analysis of antibiotic susceptibility markers suggests that most H. pylori hspAmerind and hspEAsia isolates are predicted to carry the T_2812_C mutation potentially conferring low-level clarithromycin resistance, while levels of metronidazole resistance were similar in all multilocus sequence types. In conclusion, the use of FFP phylogenetic clustering and *in silico* genotyping allows determination of genome evolution and phylogeographic clustering and can contribute to clinical microbiology by genomotyping for outbreak management and the prediction of pathogenic potential and antibiotic susceptibility.

## INTRODUCTION

Molecular typing is a cornerstone of clinical microbiology and epidemiology, as it allows the identification of pathogenic microorganisms at the genus, species, type, and subtype levels and assists in the identification of outbreaks and decisions on treatment, prevention, and policy (1). Molecular typing is commonly based on techniques such as multilocus sequence typing (MLST), which utilizes five to eight housekeeping genes conserved among all of the isolates investigated (2), or alternatives based on restriction fragment or repeat polymorphisms. Although powerful, these methods use only partial information from the genome, and use of the whole genome sequence has been shown to improve the reconstruction of genome-based bacterial phylogenetics (3) and epidemiological investigations (4, 5).

Recent advances in genome sequencing technologies allow the rapid and affordable determination of large collections of bacterial genome sequences, but this requires a concurrent increase in bioinformatic methods for the analysis of bacterial evolution and pathogen epidemiology (2). Epidemiological analyses are usually based on either single nucleotide polymorphisms (SNPs) in the core genome or whole-genome MLST with a reference genome (6), which works well for organisms with a larger core genome but is complicated for organisms that show high levels of genetic diversity or have a very small core genome. Most comparative genomic approaches rely on some sort of alignment routine for assignment of sequences shared between the genomes analyzed. Many of the genome sequences available are, however, in draft format and often consist of 15 to 1,000 contigs in random orientation, which may or may not be annotated, and those with annotations are commonly not curated. These problems can be circumvented by using alignment-free analysis methods (7, 8), which work by calculating the frequency of “words” or features that are converted to a distance matrix and subsequently used for construction of a phylogenetic tree (8). Alignment-free techniques have the advantage of being much faster than pairwise or multiple alignments and, because of their reduced complexity, are also capable of potentially handling large numbers of genome sequences, as was previously demonstrated with genome-based studies of Escherichia coli and Shigella spp. (9, 10).

Members of the genus Helicobacter colonize the gastrointestinal tracts of mammals, with the best known representative being the human gastric pathogen Helicobacter pylori, which colonizes the gastric environment of approximately half the world's population (11–13). Colonization with Helicobacter spp. is often lifelong and can elicit a strong immune response. In the case of H. pylori, this chronic infection may develop into pathologies like peptic ulcer disease and precancerous lesions and ultimately induce gastric cancer (11, 12). Early analysis of genome sequences of H. pylori revealed high levels of genome variation in both gene content and gene order (14, 15). The high levels of recombination and mutation, frequent horizontal gene transfer, and natural competence of H. pylori isolates not only contribute to this genetic variability (16, 17) but also create potential problems with several classical molecular typing techniques (18, 19).

MLST analysis does allow the subdivision of H. pylori strains into phylogeographic clusters, which are strongly correlated with human migration patterns (18, 19). However, MLST analysis of H. pylori is complicated because of the high level of genetic diversity rapidly attributing a unique MLST profile to each isolate and have the disadvantage of relying on alignments and the need for selection of a single reference genome. H. pylori has a very small core genome, estimated at 244 of the ∼1,500 genes per genome (20), and has an open pangenome (21, 22), thus making the selection of a reference genome more problematic than for most other human pathogens. The clinical importance of H. pylori as a human pathogen has resulted in the availability of a large number of sequenced genomes in public databases such as GenBank, and these genomes have given new insights into transmission and disease outcome (16). However, the analysis methods used in such studies are not easily transferable to other bacterial pathogens and do not allow the rapid analysis required for attribution, epidemiology, pathogenic potential, and antimicrobial susceptibility predictions, which would be helpful in clinical situations. Therefore, we have used H. pylori and other gastric members of the genus Helicobacter as an example for the power of the alignment-free analysis method feature frequency profiling (FFP) (8, 23). We demonstrate its usability in molecular and clinical epidemiology applications based on whole genome sequences by using 377 H. pylori genome sequences and associated genotyping data available from public databases.

## MATERIALS AND METHODS

### Analysis of whole genomes and whole proteomes of Helicobacter species.

Genome sequences were downloaded as FASTA files with contigs or complete genome sequences from PATRIC (http://patricbrc.vbi.vt.edu/portal/portal/patric/Home) (24) and the NCBI website (http://www.ncbi.nlm.nih.gov/genome/browse/). Predicted proteomes were downloaded as FASTA files with amino acid sequences of annotated features by using the PATRIC reannotation (24). Sixteen genomes/proteomes from gastric Helicobacter species were used for initial analyses (see Table S1 in the supplemental material), followed by comparison of a total of 377 H. pylori genomes/proteomes (see Table S2 in the supplemental material). Tables S1 and S2 contain GenBank/EMBL/DDBJ accession numbers of each genome sequence used, and Table S2 also contains MLST, clinical, phylogeographic, and virulence marker information for each H. pylori genome. For comparison of genome sequences assembled *de novo* with published genome sequences, the FASTQ files for nine H. pylori isolates were downloaded and extracted from the NCBI Short Read Archive and assembled with Velvet version 1.2.09 (25) by using the *k* value suggested by the Velvetk script (http://bioinformatics.net.au/software.velvetk.shtml).

### FFP.

FFP was performed with the FFP version 3.19 suite of programs (http://sourceforge.net/projects/ffp-phylogeny/) (8, 23), utilizing the FFPry program for genome sequences and FFPaa for amino acid sequences. These FFP programs generate a distance matrix, with phylogenetic trees being generated by the neighbor-joining algorithm. Analyses were performed on standard desktop and laptop computers running 64-bit Windows 7 with the 32-bit Cygwin Linux emulator and on a VirtualBox virtual computer running Bio-Linux8 (26). Input files were a single text file per genome/proteome, and bootstrapping was done with the default settings by using 100 replicates and the Phylip Consense utility (27). The FFPvprof utility was used to determine the lower word length limit (L = 11), whereas the FFPreprof utility was used to determine the upper word length limit (L = 26) (see Fig. S1 in the supplemental material), with H. pylori 26695 (G+C content of 38.9%), H. felis ATCC 49179 (G+C content of 44.5%), and H. mustelae NCTC 12198 (G+C content of 42.5%). The 16 genomes/proteomes of gastric Helicobacter species (see Table S1 in the supplemental material) were used to compare word lengths of 11 to 24 for DNA and 3 to 8 for amino acid sequences (see Fig. S2 in the supplemental material), leading to the selection of word lengths of L = 18 for DNA and L = 6 for amino acids.

### Identification of core genome SNPs.

Core genome SNPs were identified in 63 H. pylori genomes (50 complete and 13 draft genomes), covering six of the nine major multilocus sequence types (see Table S2 in the supplemental material), with the kSNP v2 software suite (6, 28–31) with standard settings and a *k*-mer value of 31, and the parSNP program from the Harvest suite (32) with the “-a 13 -c -x” switches, within Bio-Linux (26).

### Bioinformatic analyses.

Alignments were made with ClustalX2 and MEGA v5.2 (33, 34). Assignment of multilocus sequence types was done by downloading 1,409 concatenated seven-gene alleles (*atpA*, *efp*, *mutY*, *ppa*, *trpC*, *ureI*, *yphC*) from PubMLST (http://pubmlst.org/helicobacter), extraction and concatenation of the corresponding sequences from the 377 H. pylori genome sequences included, and subsequent generation of a phylogenetic tree in MEGA v5.2 (33) by using the Kimura two-parameter method and the neighbor-joining algorithm. The MLST sequences included from pubMLST were hpAfrica1 (*n* = 310), hpAfrica2 (*n* = 67), hpAsia2 (*n* = 18), hpEurope (*n* = 614), hspAmerind (*n* = 21), hspEAsia (*n* = 183), hspMaori (*n* = 80), hpSahul (*n* = 54), and hpNEAfrica (*n* = 62) (see Fig. S4 in the supplemental material). Phylogeny of ribosomal protein amino acid sequences was determined by concatenation of the RpsT (HP0076), RpsI (HP0083), RplM (HP0084), RplT (HP0126), RpmF (HP0200), RplU (HP0296), RpmA (HP0297), RpmB (HP0491), RplI (HP0514), and RpmE (HP0551) amino acid sequences, followed by alignment and generation of phylogenetic trees with MEGA v5.2. FigTree (http://tree.bio.ed.ac.uk/software/figtree/) was used for visualization of phylogenetic trees.

### Determination of H. pylori virulence and antibiotic susceptibility markers.

Genotyping by *in silico* PCR (i.e., amplification predictions) with previously described primer sets (see Table S3 in the supplemental material) was used to assess the distribution of virulence markers of H. pylori by using the FFP-derived phylogenetic trees. *In silico* PCR/genotyping was done with the microbial *in silico* typing (MIST) software package (35) and the NCBI Blast+ v2.28 executables. For the sequences of the primers used, see Table S3. The virulence factors included were the presence or absence of the *cag* pathogenicity island (PAI), the s/m/i/d subtypes of the VacA vacuolating cytotoxin, the *babA2* allele, *dupA* types, the *iceA1* and *iceA2* markers, the *jhp0917* and *jhp0918* genes, and the PZ1 and PZ2 plasticity zones (based on *jhp0945*, *jhp0947*, and *jhp0949* for PZ1 and *jhp0940* for PZ2) (36). Detection of the presence or absence of full-length Cag proteins and *cag* genes was performed by BLAST+ searches of the genome sequences with the annotated genes and amino acid sequences of the complete *cag* PAI of H. pylori 26695 (HP0520 to HP0547) as query sequences (37). The *cag3*, *cag*β, *cagZ*, *cagY*, *cagX*, *cagV*, *cagT*, *cagM*, *cagI*, *cagE*, and *cagC* genes and associated proteins were used as markers for the functionality of the *cag* PAI on the basis of the requirement for pilus formation and interleukin-8 (IL-8) production (38, 39). Prediction of antimicrobial susceptibility was based on BLAST+ searches of genomes/proteomes by using the *rdxA* and *fdxA* genes and the presence of an open reading frame encoding full-length proteins for metronidazole susceptibility (40, 41), specific regions of the 16S rRNA gene for tetracycline susceptibility (42) and of the 23S rRNA gene for clarithromycin susceptibility (43), and the GyrA protein for fluoroquinolone susceptibility (44).

## RESULTS

### Comparison of FFP with 16S rRNA genes, ribosomal proteins, and SNP-based phylogeny of gastric Helicobacter species.

**FIG 1:**
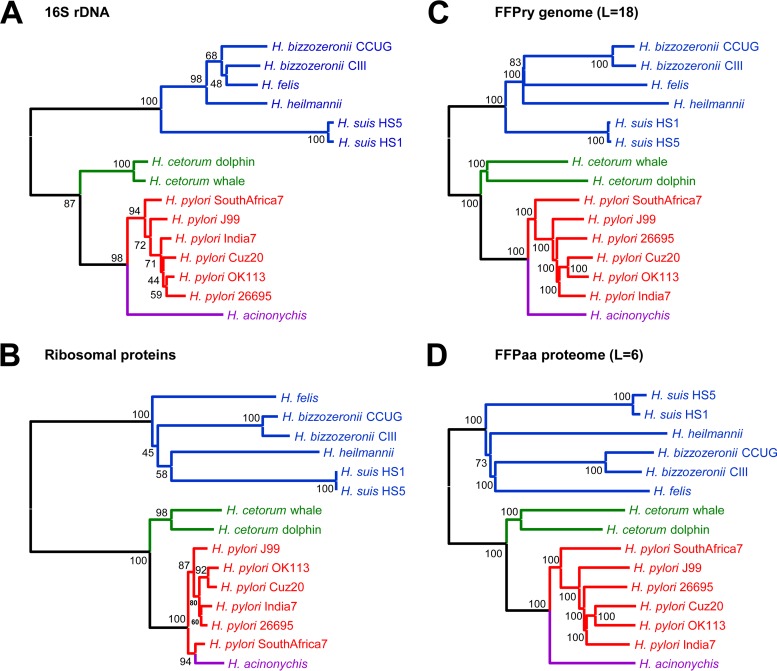
FFP-estimated phylogeny matches 16S rRNA genes and ribosomal protein-derived phylogenetic relationships within gastric Helicobacter species. Comparison of 16S rRNA gene (A)- and ribosomal protein (B)-based phylogeny with FFP-based trees of whole genomes (C) and whole proteomes (D) for gastric Helicobacter species. H. mustelae was used as the outgroup for tree rooting. Values at relevant branching points show bootstrap values (FFP, 100 replicates; 16S rRNA genes and ribosomal protein sequences, 500 replicates).

To assess how FFP-based phylogeny compares to classical phylogenetic analyses, we used 15 genome sequences and the predicted proteomes of seven gastric Helicobacter species, with G+C percentages ranging from 38 to 45%. H. pylori was represented by each of the major multilocus sequence types (hpAfrica1, hpAfrica2, hpAsia2, hpEurope, hspAmerind, and hspEAsia), whereas the other six gastric Helicobacter species were represented by the one or two genome sequences available (see Table S1 in the supplemental material). The phylogenetic trees obtained from 16S rRNA gene sequences (Fig. 1A) and concatenated amino acid sequences of 10 ribosomal proteins (Fig. 1B) were compared with the trees obtained with FFPry and FFPaa (Fig. 1C and D). All of the analysis methods used showed the same subgrouping of gastric Helicobacter species into two sublineages, one containing H. pylori, H. acinonychis, and H. cetorum and one containing H. felis, H. heilmannii, H. bizzozeronii, and H. suis. We also used these samples to confirm the optimal word length for FFPry and FFPaa analyses (see Fig. S2 in the supplemental material) and set these to L = 18 for FFPry and L = 6 for FFPaa. Higher values of L did not change the overall topology of the trees (see Fig. S2) but increased the computation time significantly (not shown), whereas shorter word lengths resulted in inconsistencies in subbranches of the trees (see Fig. S2).

**FIG 2:**
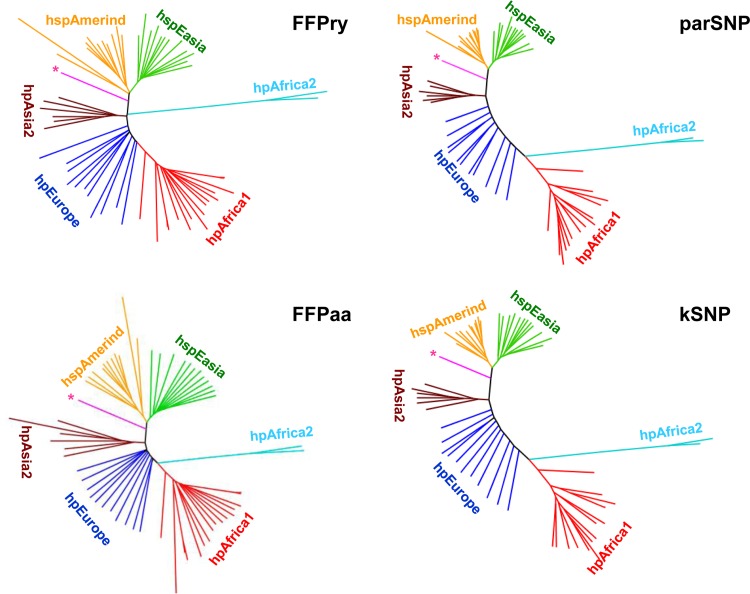
Comparison of FFP with core genome SNP-based analysis methods. A total of 63 H. pylori genomes was analyzed with two SNP-based software packages (kSNP [6] and parSNP [32]) and whole-genome- and proteome-based FFP (labeled FFPry and FFPaa, respectively). The kSNP tree is based on 86,896 SNPs present in at least half of the 63 genomes, the parSNP tree is based on 101,563 SNPs, FFPry used L = 18, and FFPaa used L = 6. The multilocus sequence types included are hpAfrica1 (red, *n* = 17), hpAfrica2 (light blue, *n* = 2), hpEurope (dark blue, *n* = 12), hpAsia2 (brown, *n* = 6), hspAmerind (orange, *n* = 11), and hspEasia (green, *n* = 14). The pink branch with the asterisk is H. pylori strain PeCan4, which is a mixed-ancestry isolate combining hspAmerind and western multilocus sequence types (22, 45).

As there is currently no whole-genome- or core genome-based MLST scheme available for H. pylori, we used two core genome SNP-based analysis tools for the comparison of whole-genome- and proteome-based FFP with existing phylogenomic analysis tools. A total of 63 H. pylori genome sequences (50 complete, 13 draft) covering six of the nine major multilocus sequence types were selected and used as the input for FFPry and FFPaa (8), kSNP (6), and parSNP (32). The phylogenetic trees generated by each of the four analysis approaches (kSNP, parSNP, FFPry, and FFPaa) showed good congruency, and each clearly separated the major multilocus sequence types (Fig. 2). The analysis included one genome (PeCan4) previously described as a chimeric strain containing a mixture of hspAmerind and western multilocus sequence types (45), and all three analyses positioned this genome correctly between hspAmerind and hpAsia2 (Fig. 2). The major difference among the SNP-based analyses, FFPaa, and FFPry is the positioning of the hpAfrica2 branch, which in the SNP-based analyses and FFPaa is located between hpEurope and hpAfrica2 and in FFPry is located between hpEurope and hpAsia2. This has relatively little impact on the overall structure of the trees and the clustering observed.

To assess whether branching in FFP trees is affected by genome assembly, we performed a *de novo* assembly of Illumina sequencing reads for nine H. pylori genome sequences from the Short Reads Archive with the Velvet short read assembler by using default settings (25), and FFP analysis was performed with the publicly available versions of the nine genomes and the *de novo* assembled genomes. Each of the *de novo* assembled genomes clustered with its publicly available counterpart (see Fig. S3 in the supplemental material), suggesting that FFP analysis is not affected by assembly specifics.

### FFP analysis of H. pylori transmission events in South African families.

**FIG 3:**
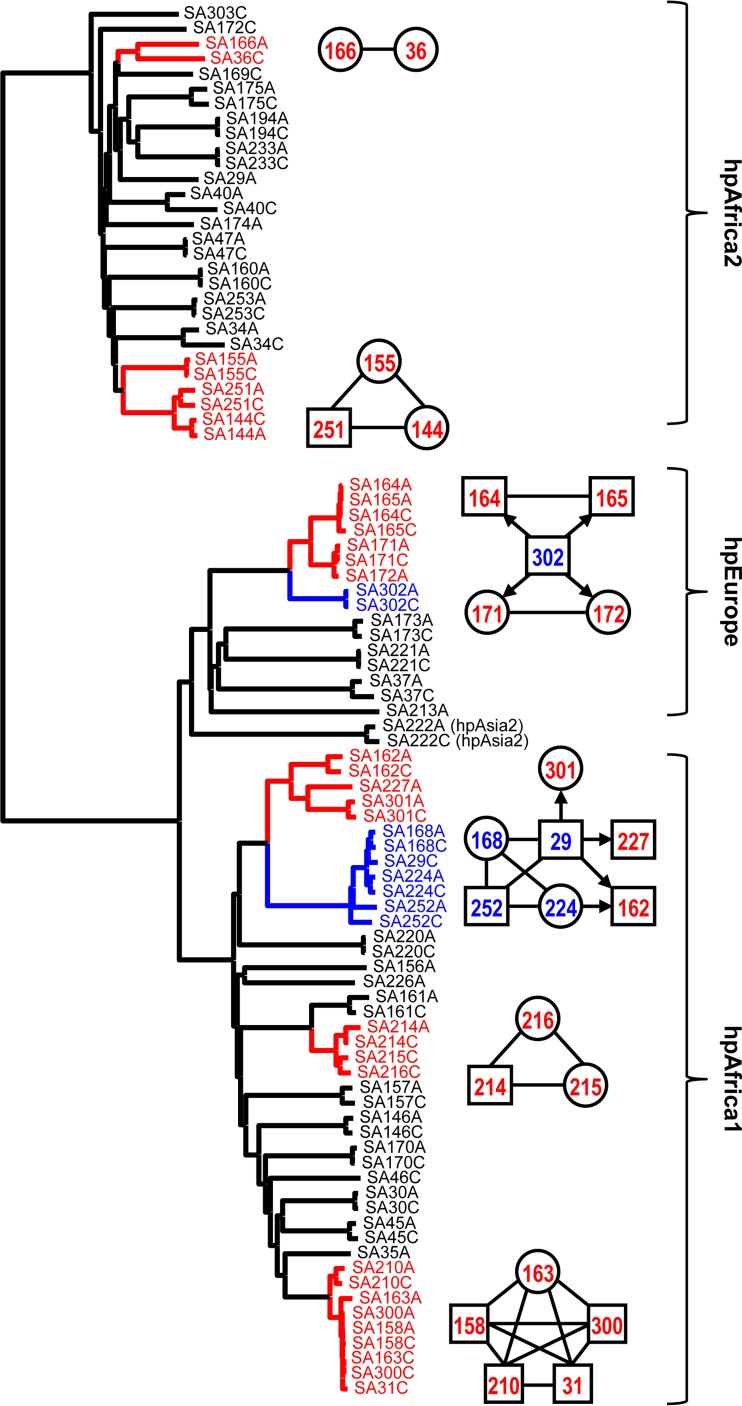
Comparison of recombination-based clustering and transmission events and FFP-based whole-genome clustering for 90 South African H. pylori isolates. A total of 90 genomes from reference 16 were used to create an FFPry-based tree and labeled according to the transmission events described in reference 16. Squares and circles represent males and females, respectively, and the number used is the unique identifier number used in reference 16. Red-labeled isolates represent recipients and isolates for which the direction of recombination could not be deduced, and blue-labeled isolates are donor isolates in the transmission indicated by the arrow. These events are matched by short branch lengths in the FFP phylogenomic tree.

To assess whether FFP analysis is comparable to mutation rate/recombination-based analysis of transmission events, we used a data set previously used to investigate H. pylori transmission within two South African families (16). In that study, the mutation rate and recombination frequency were calculated by using genomes of H. pylori isolates obtained from the antrum and corpus of the same patient, and this information was combined with the phylogeny of 786 genes to predict transmission events between individuals on the basis of calculations of the time to the most recent common ancestor (TMRCA). We used 90 genome sequences from that study, which represented four different multilocus sequence types (29 hpAfrica2, 16 hpEurope, 2 hpAsia2, and 43 hpAfrica1), and used FFP to generate trees based on the genome sequences (Fig. 3) and proteomes (see Fig. S4 in the supplemental material).

There was subgrouping of the different multilocus sequence types, with hpEurope and hpAsia2 clustering together, while hpAfrica1 and hpAfrica2 genomes are clearly separated (Fig. 3; see Fig. S4 in the supplemental material). A total of 36 out of 38 within-host couples clustered together with very short branch lengths, whereas couples were separated from each other by longer branch lengths, with the notable exceptions of the SA29 and SA172 couples, consistent with the TMRCA-based findings (16). Compared to the TMRCA-based transmission predictions, genomes clustered closely together, consistent with their shared heritage (Fig. 3). This was confirmed by using proteome-based FFPaa (see Fig. S4 in the supplemental material), which gave the same clustering of isolates implicated as between-host transmission events. Overall, this confirms that FFP can be used to analyze relatedness between H. pylori genomes.

### FFP-assigned genomotypes of H. pylori match MLST and phylogeographic assignments.

Previous work with large-scale MLST studies and *in silico* chromosome painting of a relatively small number of H. pylori genomes suggested that there is a strong correlation among whole-genome phylogeny, geographic location, and host genetic background (17, 45, 46), allowing the tracking of historical human migration patterns (18, 19). We included a total of 377 H. pylori genomes (Table 1; see Table S2 in the supplemental material) to which multilocus sequence types were assigned by mapping their positions on a neighbor-joining tree of 1,409 MLST profiles of H. pylori (see Fig. S5 in the supplemental material) and compared multilocus sequence types and geographic origins based on (sub)continents (Table 1). Within the currently available genome sequences and multilocus sequence types, those of HpAfrica1 and HpEurope isolates dominate, whereas there is only a single hpSahul genome and no hspMaori or hpNEAfrica genome available. Regarding geographic location, genomes of African and North American isolates make up almost two-thirds of the data set (Table 1).

**TABLE 1:** Characteristics of the H. pylori genomes included in this study

Multilocus sequence type	No. of isolates from:^*a*^						
	Africa	Europe	North America	South America^*b*^	East Asia	Other locations^c^	All locations
hpAfrica1^*d*^	108		60	3			171
hpAfrica2	32						32
hpAsia2	2				11	4	17
hpEurope	16	19	28	29^c^	2	5	99
hspEAsia			2	9			11
hspAmerind			4		41	1	46
hpSahul						1	1
Total	158	19	94	41	54	11	377

a Geographic assignment was based on information included in GenBank data files.

b Combines isolates from South and Central America.

c The category “other locations” contains isolates from Russia (*n = 2), India (n* = 5), and Australia (*n* = 4).

d H. pylori isolate PeCan4 is a mixture of hspAmerind and western multilocus sequence types (22, 45) and is included in hpEurope.

FFPry and FFPaa phylogenetic trees were generated from 377 genome sequences and proteomes and annotated on the basis of MLST classification and geographic origin (see Fig. S6 and S7 in the supplemental material). The genome and proteome trees showed similar topologies, which broadly matched the MLST assignments and trees. Within the FFP tree, most of the branches are relatively long (see Fig. S6), consistent with the rapid diversification of H. pylori genomes because of recombination, genetic loss, and rearrangements, as well as horizontal and lateral gene transfer (17). Comparison of the MLST and geographical data allowed the identification of specific patterns, such as within the hpAfrica1 clade, which consists of two major location subgroups (Africa and North America) (see Fig. S7, cluster A). Although ethnic information was not given in the GenBank annotation, it is tempting to speculate that hpAfrica1 from North America will be primarily from people of African American descent (see Fig. S7). Similarly, isolates from South America are primarily of either the hpEurope or the hspAmerind multilocus sequence type (see Fig. S7, clusters B and C, respectively), consistent with the initial migration from East Asia via the Bering Strait and later, from the 15th century onward, from Europe (18, 47). These subgroups are clearly separated in both the whole-genome and proteome FFP trees.

### Virulence marker distribution is partially correlated with phylogeographic lineages.

Many putative virulence markers have been described for H. pylori and show differential distribution over lineages, but whether this is connected to genome similarity is not known. The distribution of eight virulence markers was assessed for the 377 genomes, and an overview of virulence marker distribution per multilocus sequence type and geographic origin is shown in Table 2, whereas the linkage of the *cag* PAI with other virulence markers and disease outcomes are shown in Table 3. As previously described (48–50), the presence of the *cag* PAI is strongly associated with the s1 subtype of VacA and with the presence of the *babA2* allele, whereas the other virulence markers do not show a strong correlation with the presence or absence of the *cag* PAI. It should be noted that although the presence of *dupA* did not differ between *cag*-positive and *cag*-negative isolates, the *dupAS* (short) allele is more prevalent in *cag*-positive isolates, whereas the *dupAL* (long) allele is more prevalent in *cag*-negative isolates (Table 3). Clinical information was available for only 141/377 genomes, and all of the groups showed the expected higher proportion of *cag* positivity (Table 3).

**TABLE 2:** Distribution of H. pylori virulence markers and antimicrobial susceptibility over multilocus sequence types and geographic origins of H. pylori isolates

Type or origin (no.) of isolates	% of isolates positive for virulence factor:								% of isolates antibiotic resistant^*a*^			
	*cag*	*vacA* s1	*babA2*	*dupA*	*iceA2*	PZ1^*b*^	PZ2^*b*^	*jhp918*	Cla^r^^*c*^	Mtz^r^	Fq^r^	Tet^r^^*d*^
Multilocus sequence types:												
hpAfrica1 (171)	85	87	75	32	71	80	45	92	1/0	57	4	18
hpAfrica2 (32)	0	0	28	91	6	66	50	72	2/0	41	0	3
hpAsia2 (17)	94	59	100	100	47	65	35	24	0/94	59	12	0
hpEurope (98)	61	67	63	44	57	62	61	51	9/11	45	6	1
hspAmerind (11)	73	91	73	100	64	0	18	64	0/100	9	9	0
hspEasia (47)	96	98	91	60	28	40	34	38	11/81	34	11	0
Geographic origins:^*e*^												
Africa (158)	57	60	60	77	58	69	47	88	3/1	58	4	13
North America (94)	72	84	80	74	68	84	50	67	5/13	36	3	11
South America (41)	88	98	78	63	56	49	44	63	7/22	66	10	2
Europe (19)	89	89	36	26	42	53	68	32	0/16	16	0	0
East Asia (54)	100	98	91	52	31	44	39	39	11/80	44	11	0
Other (11)^*e*^	91	100	91	27	45	64	27	55	0/64	36	18	0

a Cla^r^, clarithromycin resistance; Mtz^r^, metronidazole resistance; Fq^r^, fluoroquinolone resistance; Tet^r^, tetracycline resistance.

b The presence of PZ1 and PZ2 was determined as described in reference 36.

c The first value is percent resistance based on A_2142_G, A_2142_C, and A_2143_G mutations, and the second value possible low-level resistance based on the T_2182_C mutation (52, 60).

d Tet^r^ is represented by single and double AGA_926_ mutations and represents only low levels of tetracycline resistance.

e Geographic origin is based on information included in GenBank data files. The category “other” includes Russia (*n* = 2), India (*n* = 5), and Australia (*n* = 4).

**TABLE 3:** Correlation of virulence markers and disease outcomes with the presence of the *cag* PAI

Characteristic (no. of isolates)	% of 276 isolates *cag*^+^	% of 101 isolates *cag* negative
Virulence markers:		
*vacA* s1 (277)	93	19
*babA2* (268)	80	47
*dupA* (short/long) (253)	67 (48/20)	66 (15/51)
*iceA2* (208)	56	53
*jhp918* (261)	69	69
PZ1 (249)	68	59
PZ2 (177)	45	51
Disease outcomes:		
Gastritis (66)	77	23
Peptic ulcer (41)	88	12
Atrophy/metaplasia/dysplasia (10)	70	30
Gastric cancer (11)	100	0
MALT lymphoma (1)	0	100
Nonulcer dyspepsia (12)	100	0
Other (12)^*a*^	92	8
Unknown (224)	66	34

a The category “other” consists of isolates used in mouse and gerbil infection experiments and one isolated from a cat.

**FIG 4:**
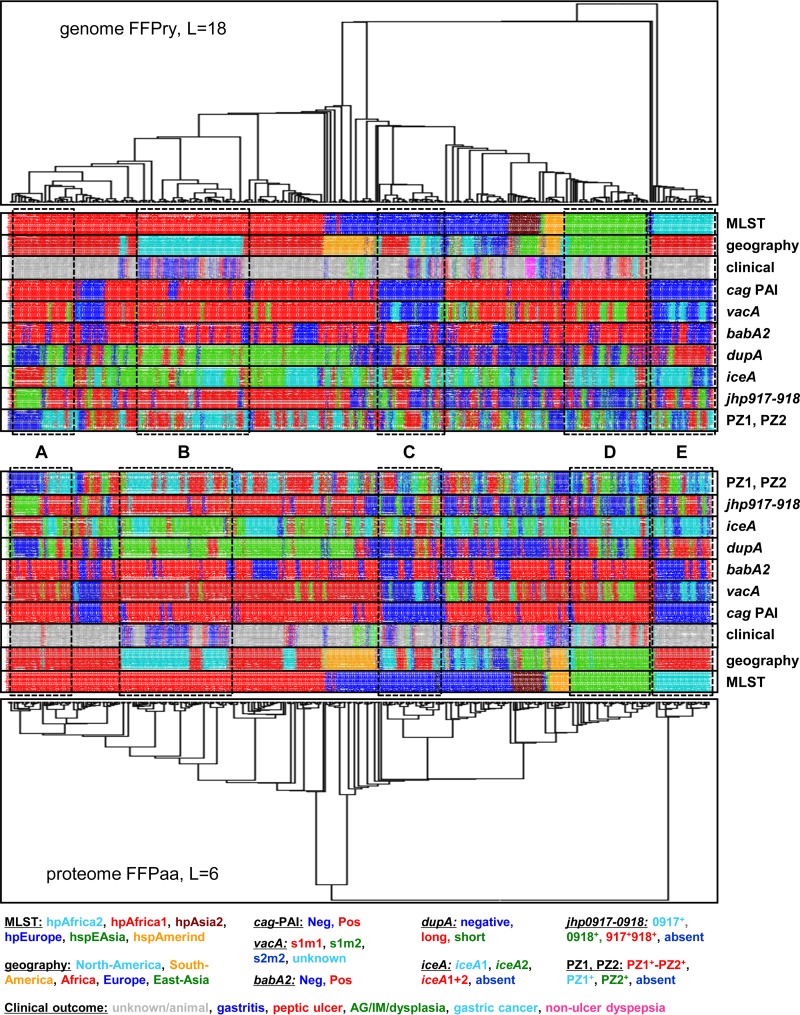
The distribution of H. pylori virulence markers is partially correlated with phylogeographic clustering. The distribution of seven known virulence markers in the 377 H. pylori genomes was assessed by *in silico* PCR with MIST (35) and previously published primer sequences (see Table S3 in the supplemental material) and plotted on an FFPry genome-derived phylogenetic tree with L = 18 and on an FFPaa proteome-derived phylogenetic tree with L = 6. The trees were transformed by using the “proportional” setting of FigTree for presentational purposes; for the nontransformed trees with the original branch lengths, see Fig. S6 in the supplemental material. For each sample, the multilocus sequence type, continent of isolation, and clinical outcome are also shown (if available), and the color code is shown at the bottom. The boxed areas show a subgroup of *cag*^+^, *vacA* s1m1, *babA2*^+^, and *jhp0918*^+^ hpAfrica1 genomes that are mixed for other virulence markers (A); a group of hpAfrica1 genomes from North America that are *cag*^+^, *vacA* s1m1, *babA2*^+^, *dupAS*, *iceA2*^+^, *jhp0917*^+^, *jhp0918*^+^, and PZ1^+^ (B); a group of hpEurope *cag*-negative *vacA* s2m2 genomes that are mixed for all other virulence markers (C); a group of hspEAsia genomes that are *cag*^+^, mixed *vacA* s1m1 and s1m2, *babA2*^+^, and *iceA1*^+^ but mixed for other virulence markers (D); and a group of hpAfrica2 isolates that are *cag* negative, *vacA* s2m2, and mostly *dupAL*^+^ but mixed for other virulence markers (E). The trees are rooted with the H. acinonychis Sheeba genome/proteome (see Table S1 in the supplemental material) as the outgroup.

The FFPry and FFPaa trees obtained with the 377 H. pylori whole genomes and proteomes (Fig. 4; see Fig. S6 in the supplemental material) were coupled to the status of the virulence markers to visualize specific patterns in distribution compared to multilocus sequence types/genomotypes and geographic origins. In general, the virulence marker distribution over the phylogenomic tree and MLST classifications matched the previously described associations, but the grouping on genomic relatedness showed some differences between geographic regions (Fig. 4, examples marked A to E). Compared to the different multilocus sequence types and geographic distributions, the availability of clinical information was restricted mostly to subgroups within the six major multilocus sequence types (Fig. 4). While correlations between disease outcomes and the distribution of virulence markers may exist in this collection, the data set used here combines genomes from many different studies and hence is unlikely to represent an unbiased collection. Hence, such associations could well be based on collection bias rather than a true biological linkage.

**FIG 5:**
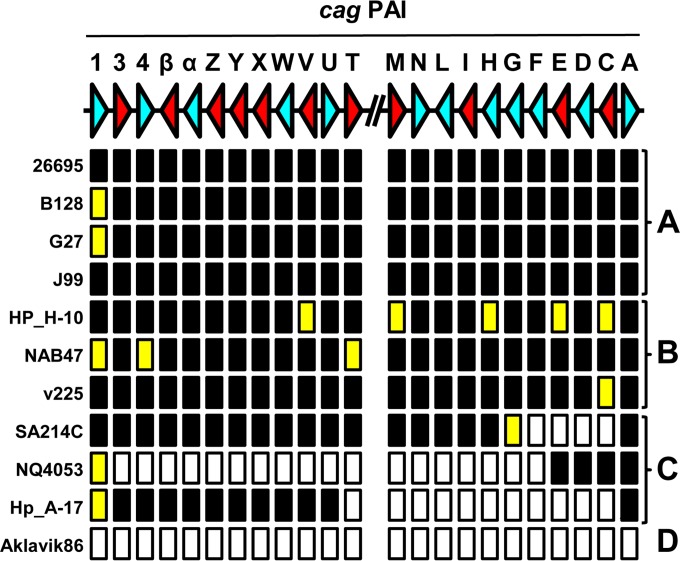
Subdivision of presence or absence and functionality analysis of the H. pylori *cag* PAI. BLAST+ analysis of H. pylori genomes and encoded proteomes was used to determine whether all of the genes of the *cag* PAI were present or absent and, if present, whether they are predicted to encode a full-length protein. The top line shows a schematic representation of a gene map of the two arms of the *cag* PAI of H. pylori 26695, with each gene being represented by an arrowhead and letters representing the gene names. The direction of the arrowhead shows the transcriptional orientation. Genes essential for interleukin induction or pilus formation (38, 39) are red, whereas nonessential genes are light blue. The absence or presence of the gene and encoded protein are indicated below the gene map, with black rectangles indicating that both the gene and protein are present, yellow rectangles indicating the presence of the gene but absence or truncation of the corresponding protein, and white rectangles indicating the absence of the gene and protein. The H. pylori genomes could be subdivided in four classes based on the *cag* PAI status as follows: A, complete and functional *cag* PAI; B, complete *cag* PAI but functionally negative because of lack of expression of essential components; C, contains only fragments of the *cag* PAI, with large fragments missing; D, *cag*-negative genome lacking the *cag* PAI completely. Representative examples of each subgroup are shown. For full analyses of the individual genes of the *cag* PAI, see Table S4 in the supplemental material.

Besides *in silico* PCR, we tested whether the *cag* PAI should give a functional type IV secretion system for induction of IL-8. Previous studies showed that while CagA is not required for functionality, 11 other proteins are required for pilus formation and interleukin induction (38, 39). Hence, BLAST searches were used to test for the presence of genes of the *cag* PAI of H. pylori 26695, as well as whether the complete and uninterrupted open reading frames encoding the Cag proteins were present (see Table S4 in the supplemental material). Of the genomes negative for *cag* genes by PCR, two hspAmerind genomes (Shi417 and Shi470) and one hpAfrica1 genome (GAM96Ai) were subsequently shown to be *cag* positive. Of the 276 genomes thus classified as *cag* positive, 18 are predicted to be functionally *cag* negative on the basis of the absence of 1 of the 11 proteins essential for the functionality of the *cag* PAI (38, 39) (Fig. 5; see Table S4 in the supplemental material). Furthermore, of the 101 genomes classified as *cag* negative, 7 contained parts of the *cag* PAI, ranging from a large deletion of the region from *cagF* onward in isolate NQ4053 to a deletion of only genes *cagC* to *cagG* in SA214C (Fig. 5; see Table S4). There were no *cag*-positive isolates that lacked the *cagA* gene, indicative of the crucial role of CagA in the function of the *cag* PAI.

### Use of genome sequences for prediction of antibiotic susceptibility in H. pylori.

Treatment of H. pylori infection often relies on therapies where two antibiotics are combined with a proton pump inhibitor and an optional bismuth component but is hampered by the widespread antibiotic resistance of H. pylori (51). Resistance to commonly used antibiotics such as metronidazole, clarithromycin, tetracycline, and fluoroquinolones is based mostly on point mutations in oxidoreductase genes, the 23S and 16S rRNA genes, and the gyrase gene *gyrA*, respectively (41, 42, 44, 52). Hence, we investigated the 377 genomes for such mutations to predict the antibiotic susceptibility profile of each of the isolates (Table 2; see Table S5 in the supplemental material). The majority of the hpAsia2, hspAmerind, and hspEAsia genomes contain the T_2182_C mutation in the 23S rRNA gene and hence may show a low level of clarithromycin resistance (52), while only a small proportion of hpAfrica1, hpAfrica2, and hpEurope genomes was predicted to carry this mutation potentially conferring low-level clarithromycin resistance. The high-level clarithromycin resistance-associated A_2142_G, A_2142_C, and A_2143_G mutations were rare and were present in only 3, 1, and 14 of 377 genomes, respectively. In contrast, metronidazole resistance (based on the presence of a truncated RdxA or FrxA protein or both proteins) is relatively rare in the hspAmerind and hspEAsia genomes (10% and 30%, respectively) but present in 45 to 67% in hpAfrica1, hpAfrica2, hpAsia2, and hpEurope genomes, although these results need to be interpreted with caution, as the link between *rdxA* and *frxA* mutations and metronidazole resistance is not absolute, and other (nongenetic) mechanisms of metronidazole resistance have been described (53). Only a few genomes contained a mutation at position N87 or D91 in the GyrA protein, which is known to confer fluoroquinolone resistance, including Malaysian genomes known to be fluoroquinolone resistant (43). The number of genomes containing 16S rRNA gene mutations known to confer tetracycline resistance was relatively low, with none of the genomes containing the triplet AGA_926-928_TTC mutation in the 16S rRNA gene (54), which is known to confer high-level tetracycline resistance, but several single and double mutations at these positions, which give low-level tetracycline resistance, were detected (42, 55). Finally, 4/377 (1.1%) genomes contain the genotypes predicted to confer resistance to three antibiotics (3 clarithromycin, fluoroquinolone, and metronidazole and 1 fluoroquinolone, metronidazole, and tetracycline), whereas 16/377 (4.2%) genomes combined the genotypes to confer clarithromycin and metronidazole (*n* = 10), clarithromycin and fluoroquinolone (*n* = 26), or fluoroquinolone and metronidazole resistance (*n* = 4) (see Table S5 in the supplemental material).

## DISCUSSION

Although molecular information was already used in typing and epidemiology applications before the advent of genome sequencing 20 years ago (56), the rapid changes in genome sequencing technologies and capabilities are now feeding into clinical microbiology and epidemiology. With the cost of sequencing dropping, this means that genome sequencing is likely to become the new standard for molecular typing and epidemiology. Since genome sequences contain all of the information used in other DNA sequence-based applications, genome sequences give backward compatibility with earlier test methods, which can be reproduced *in silico*. Although the field of molecular epidemiology based on genome sequences is still in development, two analysis methods have emerged that could be considered the standard: core genome SNP analysis and MLST based on whole genomes, pangenomes, or core genomes (2). Both analysis methods require preprocessing of data, such as alignment, strand determination, and relative positioning, and require a significant proportion of the genomes included to be conserved to allow direct comparison. Also, these analysis methods rapidly become processing intensive if scaled up, often requiring specialist hardware and software. In this study, we have applied the alignment-free analysis method FFP (8) for construction of whole-genome-based phylogenetic trees, which has the advantage of not requiring preprocessing of genome sequences other than assembly and can handle large collections of genomes without the need for specialist hardware or software.

The suitability of FFP for phylogenetic analyses was shown by using a set of genome sequences of the bacterial pathogen H. pylori, a bacterial pathogen that has been challenging for molecular epidemiology applications because of its high levels of genetic diversity and its small core genome (20). We populated the H. pylori phylogenetic trees thus obtained with information on the country or continent of isolation, the presence or absence of virulence markers, and predicted antimicrobial susceptibility. Unfortunately, many of the genome sequences available for H. pylori do not include clinical information, such as disease outcome, as this information was not available for 224 of the 377 genomes included, while 12 of the 377 genomes were from isolates used with animals. The primary aim of this study was to show the possibilities offered by using FFP analysis for rapid genome- and proteome-based phylogenetics and was not intended as an in-depth study of possible correlations among disease outcome, virulence factors, and phylogenetic information. The genomes included were obtained from many different studies, and for many there is no additional information on sampling strategies and other important characteristics, and hence, the correlations shown in Fig. 4 were not statistically evaluated. This does demonstrate the need for GenBank/EMBL to include more of such data with genome sequence submissions where available, as other associations between genetic features and clinically relevant parameters cannot be analyzed from the available genome data alone.

As a benchmark for comparison, we have used core genome SNP analysis and seven-gene MLST (Fig. 2), but as there is no validated genome-based MLST scheme for H. pylori, we were unable to compare genome-based MLST and FFP. The major advantages of SNP- and MLST-based analyses are that there is functional and/or positional information included with the data, whereas FFPry- and FFPaa-based data are not easily converted to reveal such information. Also, because of the conversion of DNA sequences to purine-pyrimidine couples, it can be expected that genome SNP and genomic MLST analyses are more sensitive for discrimination of very closely related isolates. Within the 377 isolates investigated, those isolated from the same person clustered very closely together, as shown in Fig. 3 (see also Fig. S4 in the supplemental material) (16). Hence, FFP, in our opinion, cannot completely replace SNP- or MLST-based analysis but rather adds to the toolbox for the analysis of microbial genomes. For molecular epidemiology purposes, it can be used to rapidly determine what the closest relatives of unknown isolates are, as shown in Fig. 3 (see also Fig. S3 and S4), and can easily be expanded to include new whole genomes and proteomes when these become available.

This is not the first study to use FFP for bacterial genomics. Besides a study comparing E. coli isolates (10), one of the earlier papers using FFP investigated the phylogeny of Shigella and E. coli and compared the use of FFP of whole genome sequences with FFP based on core genome features only (9). The former is not a true representation of genome evolution, as the whole genome sequence includes sequences obtained by horizontal gene transfer, as well as niche-induced adaptations, whereas the latter are more likely to represent the true evolutionary phylogeny. In the case of H. pylori, this is apparent only by the difference in where the hpAfrica2 genomes branch off compared to the hpAfrica1 and hpEurope genomes in Fig. 2, although this difference is, interestingly, not observed when using the proteome from these genomes (Fig. 2). Our results show that while it may not be completely accurate in assessing evolutionary relationships between genomes in H. pylori, the FFPry and FFPaa trees are sufficiently similar to suggest that FFP is usable for assessing genomic relatedness between H. pylori isolates. We confirmed this by comparing *de novo* assembled H. pylori genomes with their published counterparts, in which the assembled genomes all clustered with the published version (see Fig. S3 in the supplemental material), and by using a recombination-based H. pylori transmission study in South Africa, where again the clustering observed in the whole-genome and proteome-based trees matched the published transmission patterns (Fig. 3; see Fig. S4 in the supplemental material) (16). Hence, we are confident that FFP-based whole-genome and proteome-based analyses can be used for H. pylori phylogenomic analyses and by extension also for other bacterial genera and are very powerful when combined with genotyping analyses, as shown in Fig. 4. A major difference between SNP- and MLST-based analyses is that FFP utilizes the full genome, including the accessory genome, while SNP- and MLST-based methods are limited to core or conserved regions of the genome.

Using FFP for these analyses has several advantages. First, FFP analysis is reference free and hence not dependent on the choice of a suitable reference genome that may restrict interstudy comparisons, as well as future expansion of data sets. Second, because it is alignment free, it does not depend on assembly characteristics, contig orientation, or contig order. Random reordering of contigs does not affect the clustering of genomes, as FFP is not dependent on positional information, only on whether words occur in the genome. Third, FFP has modest hardware requirements, and analyses can be done on consumer desktop and laptop computers running Linux in a virtual machine or through a Linux emulator such as Cygwin. For example, generation of the FFPry phylogenetic tree with L = 18 shown in Fig. 2 (without bootstrap) takes ∼3 min 30 s on a modern Windows 7 desktop computer, whereas the FFPaa tree with L = 6 takes ∼11 min. We used L = 18 for genome analyses and L = 6 for proteome analyses, based on the analysis shown in Fig. 1 (see also Fig. S1 and S2 in the supplemental material), as trees with L = >17 and L = >6 converged into a single, stable topology (see Fig. S2) that, in our opinion, represent a good balance between speed and accuracy of the phylogenetic analyses. A typical FFPry analysis with L = 18 takes <1 h for the H. pylori 377-genome data set on a standard desktop computer, making it both cheaper and independent from larger computing resources. In addition to allowing analysis of genome sequences, FFP also allows comparison of annotated proteomes from the respective genomes (Fig. 4; see Fig. S7 in the supplemental material), and these gave very comparable phylogenetic trees, supporting the relatedness assigned by FFP based on whole genomes (Fig. 4; see Fig. S7). SNP-based comparison methods are particularly effective with genomes with relatively low numbers of SNPs and inversions, insertions, and deletions, and hence, the SNP-based methods struggled with H. pylori genomes; the parSNP program was unable to analyze the full 377-genome data set because the genomes have <10% conserved sequences.

Although the first H. pylori genome sequence was published in 1997 (37), it is only in the last few years that the number of available H. pylori genome sequences has dramatically increased. In our study, we have included 377 H. pylori sequences, and 376 of these belong to six of the nine major multilocus sequence types (18, 57). At the time of the analysis, there was only a single hpSahul genome sequence available (46), and there are no hpNEAfrica or hspMaori genome sequences publicly available. However, since the phylogenetic trees obtained are highly similar to the one obtained by MLST analysis (see Fig. S5 to S7 in the supplemental material), it can be expected that the hpNEAfrica and hpSahul genomes will cluster with the hpEurope genomes, whereas hspMaori will likely cluster between the hspAmerind and hspEAsia genomes. Similar distributions were recently reported for Malaysian isolates of H. pylori (58). Thus, MLST analysis is still very powerful and has good predictive power for genomotypes and possible phylogeographic implications (see Fig. S7 in the supplemental material; Table 1) but, like genomotyping, has little predictive power for virulence markers (Fig. 4 and Table 2; see Table S2 in the supplemental material). As there is an as-yet-unclear relationship between the presence of the *cag* PAI and the presence or absence of other virulence markers like *vacA* s1 and *babA2*^+^ (Table 3; see Table S2 in the supplemental material), there is need for caution in interpreting the linkages and absence of linkages observed in Fig. 4. However, this study is the first to be able to visualize the relationships among genomotype, phylogeography, and virulence marker distribution with genome sequences and proteomes on a scale of hundreds of H. pylori genome sequences.

The availability of genome sequences also allowed for the prediction of the antibiotic susceptibility profiles of the genomes included, and this was done for four of the antibiotics commonly used for H. pylori (clarithromycin, metronidazole, fluoroquinolone, and tetracycline) (see Table S5 in the supplemental material) for which genetic mechanisms of resistance are known, and our *in silico* predictions corresponded to 21 strains for which experimental data were available (43, 59) with regard to fluoroquinolone and tetracycline resistance and for clarithromycin resistance with regard to the A_2142_G, A_2142_C, and A_2143_G mutations in the 23S rRNA gene. As the contribution of the T_2182_C mutation to clarithromycin resistance has been questioned (60), the genotypic data with regard to that mutation need to be interpreted with caution, as our analysis indicates that the majority of hpAsia2, hspAmerind, and hspEAsia strains would be resistant, a feature that is not matched by actual clinical data from those regions (43, 51, 59). Similarly, assigning metronidazole resistance based on truncation of the RdxA and FrxA proteins can lead to overestimation of resistance levels, as 5/21 strains were not reported as metronidazole resistant (43, 59) but have a truncated FrxA protein. Also, as metronidazole resistance can also be mediated by nongenetic, redox-based mechanisms (53), genome sequence-based predictive analyses can currently be used only as a tool to forecast potential resistance and, for now, still requires experimental confirmation. Although improvements in these analyses are still needed, the results obtained do show the power of *in silico* prediction of antimicrobial susceptibility.

In conclusion, we show here that FFP allows the rapid but sensitive clustering of H. pylori isolates based on relatedness of whole genomes and whole proteomes without requiring prior knowledge of genome annotation, mutation rates, or selection of a reference genome. The resulting phylogenetic trees of H. pylori genomes and proteomes correspond to MLST-based assignment of isolates and support the previously observed phylogeographic signal within H. pylori. When we combined them with genotyping data, we could show that some of the H. pylori virulence markers (*cag*, *vacA*, *babA2*) do have a link with phylogeographic clusters, while other virulence markers (such as *dupA*, *iceA*, and the plasticity zones PZ1 and PZ2) do not show such a correlation. The expected expansion of genomic information for H. pylori is likely to show new patterns of genetic diversification in this intriguing human bacterial pathogen.

## Supplementary Material

Supplemental material
