# Supplementary material for: Use of Alignment-Free Phylogenetics for Rapid Genome Sequence-Based Typing of Helicobacter pylori Virulence Markers and Antibiotic Susceptibility
Source: J Clin Microbiol. 2015 Aug 18;53(9):2877–88. doi: 10.1128/JCM.01357-15 (PMC4540916; doi:10.1128/JCM.01357-15)
Supplement: Supplemental material [file supp_53_9_2877__index.html]

Use of Alignment-Free Phylogenetics for Rapid Genome Sequence-Based Typing of Helicobacter pylori Virulence Markers and Antibiotic Susceptibility — Supplemental material 

# Use of Alignment-Free Phylogenetics for Rapid Genome Sequence-Based Typing of Helicobacter pylori Virulence Markers and Antibiotic Susceptibility

## Supplemental material

- Supplemental file 1 -

  Fig. S1 (Determination of optimal feature length for three *Helicobacter* species with varied G+C percentages), S2 (Effect of feature length selection on FFP whole-genome and FFP whole-proteome trees of the gastric *Helicobacter* species shown in Fig. 1), S3 (*De novo*-assembled *H. pylori* genomes branch with their published versions in FFP analysis), S4 (FFP analysis of whole proteomes and SNP-based genome clustering for 90 South African *H. pylori* isolates), S5 (Multilocus sequence typing of *H. pylori* isolates used in this study), S6 (Untransformed trees in traditional and radial formats of the whole-genome phylogenetic tree and the whole-proteome FFP tree shown in Fig. 4 and 5), and S7 (Feature frequency profiling of whole genomes and whole proteomes matches phylogeographic molecular epidemiology of *H. pylori*) and Tables S1 (Accession numbers, isolate designations, MLST types [for *H. pylori*], and hosts from which the strains were isolated), S2 (Overview of *H. pylori* genome sequences included in this study, with genotype, MLST type, geography, and disease outcome information), S3 (Primers used for *in silico* PCR genotyping of *H. pylori* virulence markers), S4 (Presence/absence analysis of genes belonging to the *H. pylori cag* pathogenicity island), and S5 (Prediction of antibiotic resistance encoded by *H. pylori* genomes)

  PDF, 1.5M
