# Supplementary material for: Use of Alignment-Free Phylogenetics for Rapid Genome Sequence-Based Typing of Helicobacter pylori Virulence Markers and Antibiotic Susceptibility
Source: J Clin Microbiol. 2015 Aug 18;53(9):2877–88. doi: 10.1128/JCM.01357-15 (PMC4540916; doi:10.1128/JCM.01357-15)
Supplement: Supplemental material [file JCM.01357-15_zjm999094462so1.pdf]

SUPPLEMENTARY INFORMATION FOR

Use of alignment-free phylogenetics for rapid genome sequence-based typing of *Helicobacter pylori* virulence markers and antibiotic susceptibility

Arnoud H. M. van Vliet [a,*], Johannes G. Kusters [b]

Table of contents:

- **Figure S1.** Determination of optimal feature length for three *Helicobacter* species with varied G+C percentages.
- **Figure S2.** Effect of feature length selection on FFP whole genome (A) and FFP whole proteome (B) trees of the gastric *Helicobacter* species shown in Figure 1.
- **Figure S3.** *De novo* assembled *H. pylori* genomes branch with their published versions in FFP analysis.
- **Figure S4.** FFP-analysis of whole proteomes (A), and SNP-based genome clustering (B) for 90 South-African *H. pylori* isolates.
- **Figure S5.** Multi Locus Sequence Typing of *H. pylori* isolates used in this study.
- **Figure S6.** Untransformed trees in traditional (left) and radial (right) format of the (A) whole genome phylogenetic tree and (B) the whole proteome FFP tree used in Figures 4 and 5.
- **Figure S7.** Feature frequency profiling of whole genomes (top) and whole proteomes (bottom) matches phylogeographic-based molecular epidemiology of *H. pylori*.
- **Table S1.** Accession number, isolate designation, MLST-type (for *H. pylori*), and host from which the strain was isolated is indicated.
- **Table S2.** Overview of *H. pylori* genome sequences included in this study, with genotypes, MLST, geographic and disease outcome information.
- **Table S3.** Primers used for in silico PCR genotyping of *H. pylori* virulence markers.
- **Table S4.** Presence / absence analysis of genes belonging to the *H. pylori cag* pathogenicity island
- **Table S5.** Prediction of antibiotic resistance encoded in *H. pylori* genomes.
- **References Supplementary Information**

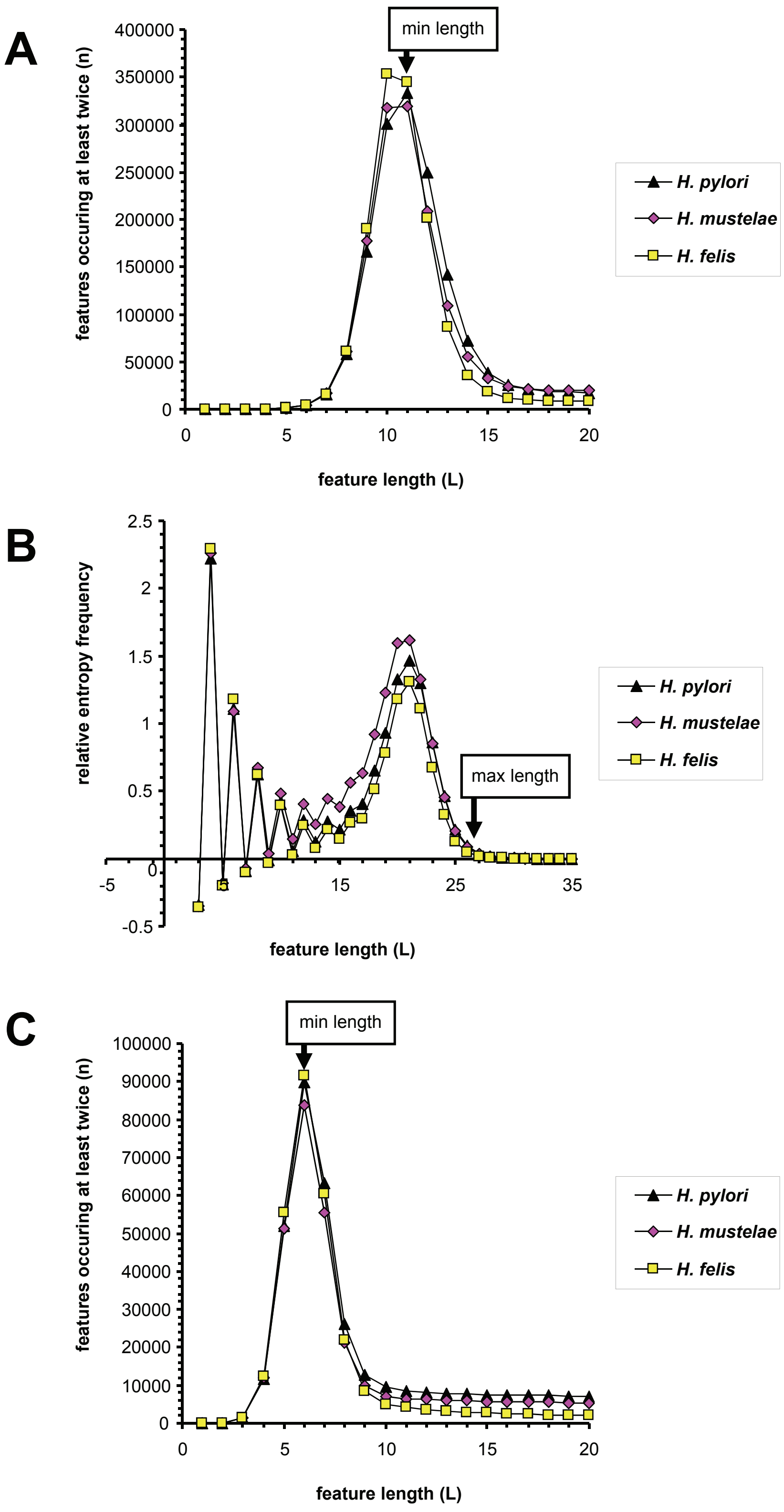

Figure S1. Determination of optimal feature length for three *Helicobacter* species with varied G+C percentages. Graphs A and C show the determination of the minimum feature length for DNA (A) and amino acid sequences (B) using the ffpvprof utility (1). Graph B shows the determination of the maximum feature length for DNA using the ffpreprof utility (1); we were unable to run this utility with amino acid sequences. The minimum feature length for DNA is 11 and the maximum length is 26, whereas the minimum feature length for amino acid sequences is 6. The genomes used are *H. pylori* 26695 (G+C=38.9%), *H. felis* ATCC 49179 (G+C= 44.5%) and *H. mustelae* NCTC 12198 (G+C=42.5%).

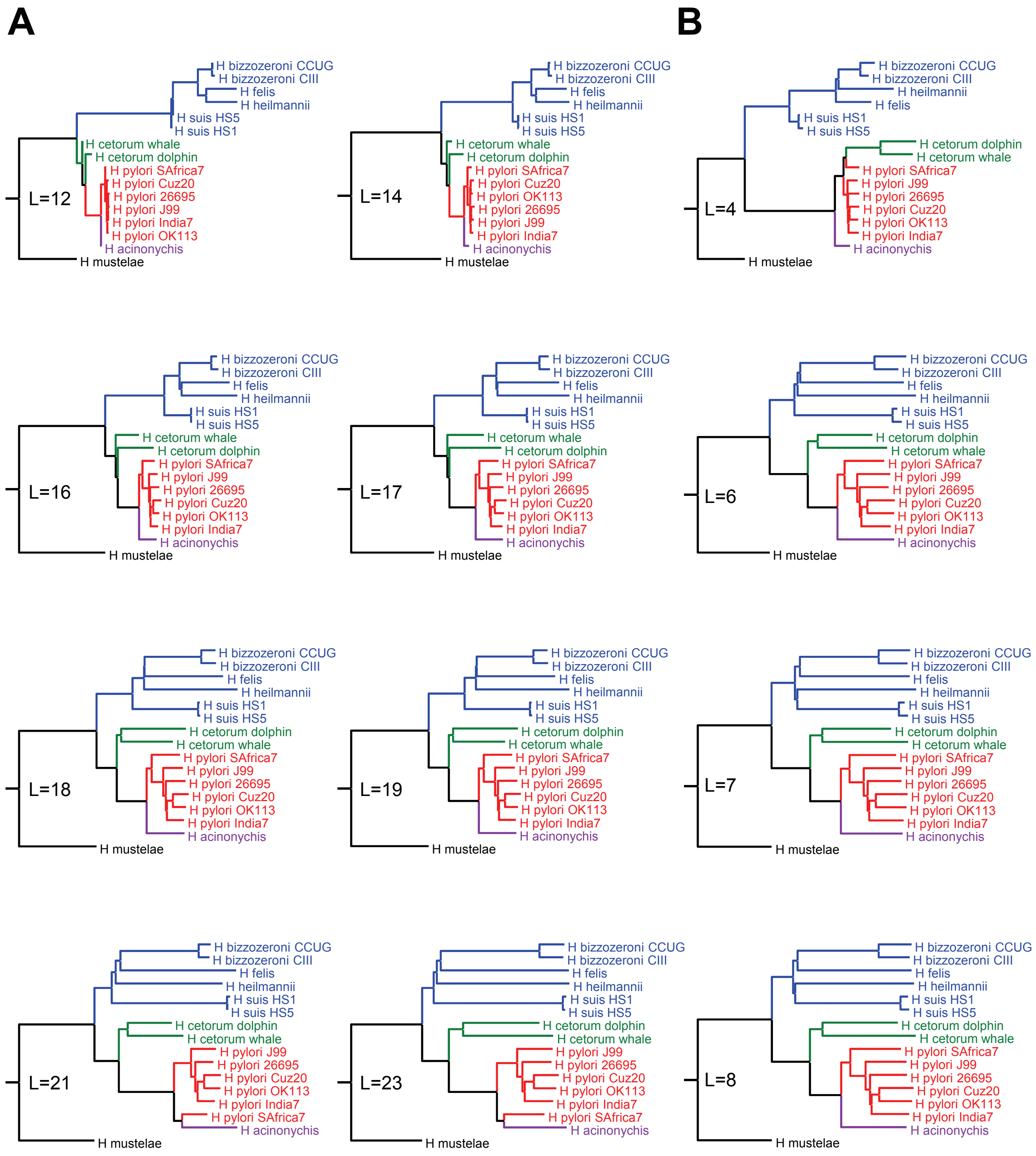

Figure S2. Effect of feature length selection on FFPry whole genome (A) and FFPaa proteome (B) trees of the gastric *Helicobacter* species shown in Figure 1. *H. mustelae* was used as outgroup for tree rooting. Coloring of branches is identical to Figure 1. The feature length was varied between L=11 and L=23 for DNA (RY), and between L=4 and L=8 for amino acid sequences. For RY-based phylogenomic trees, increasing the feature length results in better resolution and longer branch lengths, but also computational time (not shown).

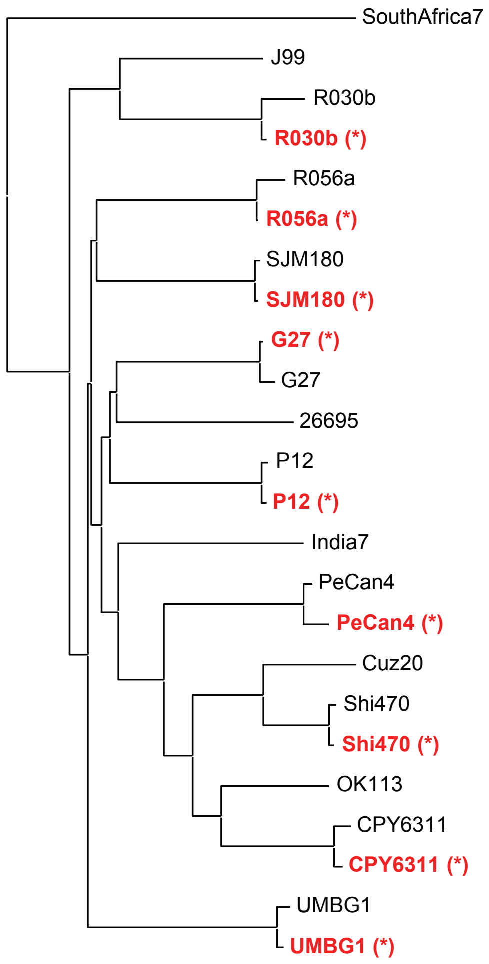

Figure S3. *De novo* assembled *H. pylori* genomes branch with their published versions in FFP analysis. Samples labelled red with an asterisk are genomes assembled *de novo* with the Velvet assembler (2) and used for FFPry analysis with L=18. Each of the newly assembled genomes branches clearly with the published version.

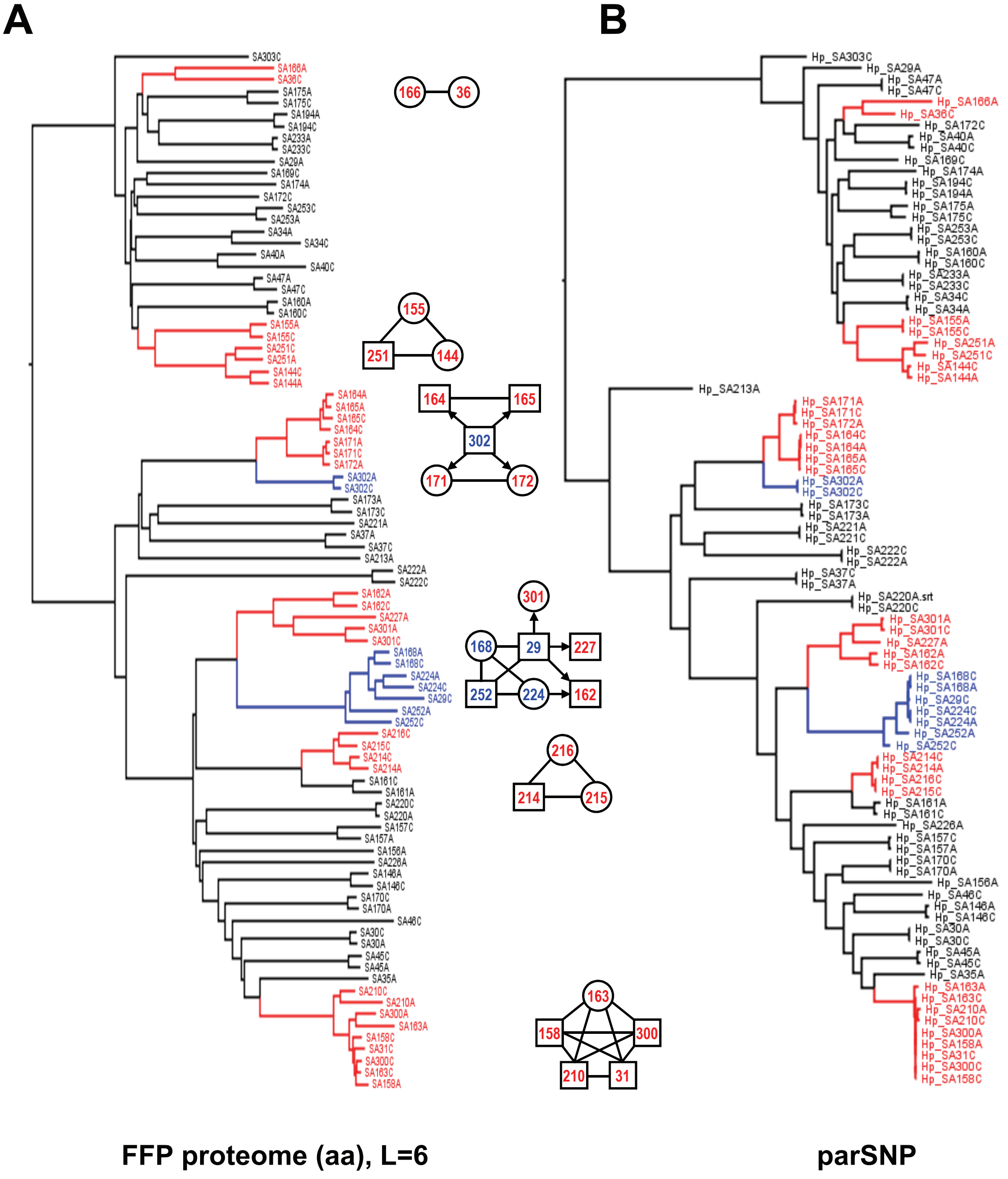

Figure S4. FFP-analysis of whole proteomes (A), and SNP-based genome clustering (B) for 90 South-African *H. pylori* isolates. The isolates are the ones shown in Figure 3 with whole genome FFP analysis (3). Squares and circles represent males and females, respectively, and the number used is the unique identifier number used in (3). Red-labeled isolates represent recipients and isolates for which the direction of recombination could not be deduced, blue-labeled isolates are donor isolates in the transmission indicated by the arrow. The FFP tree (A) was made using the amino acid sequences with L=6, the SNP-based tree (B) was created using parSNP.

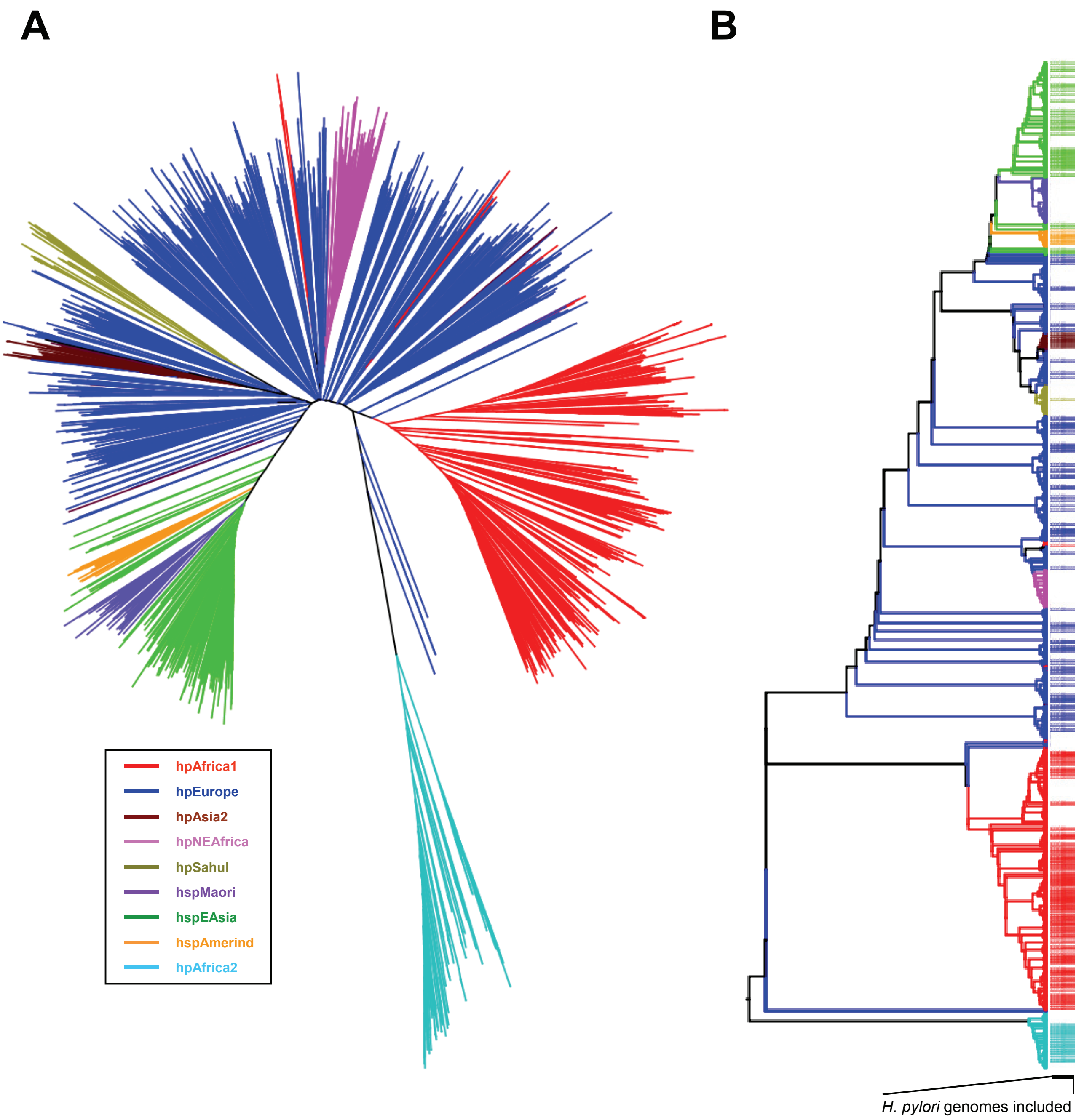

Figure S5. Multi Locus Sequence Typing of *H. pylori* isolates used in this study. (A) MLST using concatenated sequences of seven housekeeping genes (*atpA*, *efp*, *mutY*, *ppa*, *trpC*, *ureI*, *yphC*), aligned using the Muscle algorithm in MEGA 5.2 (4) and a Neighbor-Joining tree using the Kimura 2-parameter setting and pairwise deletion. The tree includes 1409 profiles from the PubMLST database (310 hpAfrica1, 67 hpAfrica2, 18 hpAsia2, 614 hpEurope, 21 hspAmerind, 183 hspEAsia, 80 hspMaori, 54 hpSahul and 62 hpNEAfrica) and MLST sequences from the 377 *H. pylori* genomes used in this study. (B) Tree showing the position of the MLST profiles of the 377 genomes included in this study, using the tree transformed with the proportional setting of FigTree 1.4.0.

A

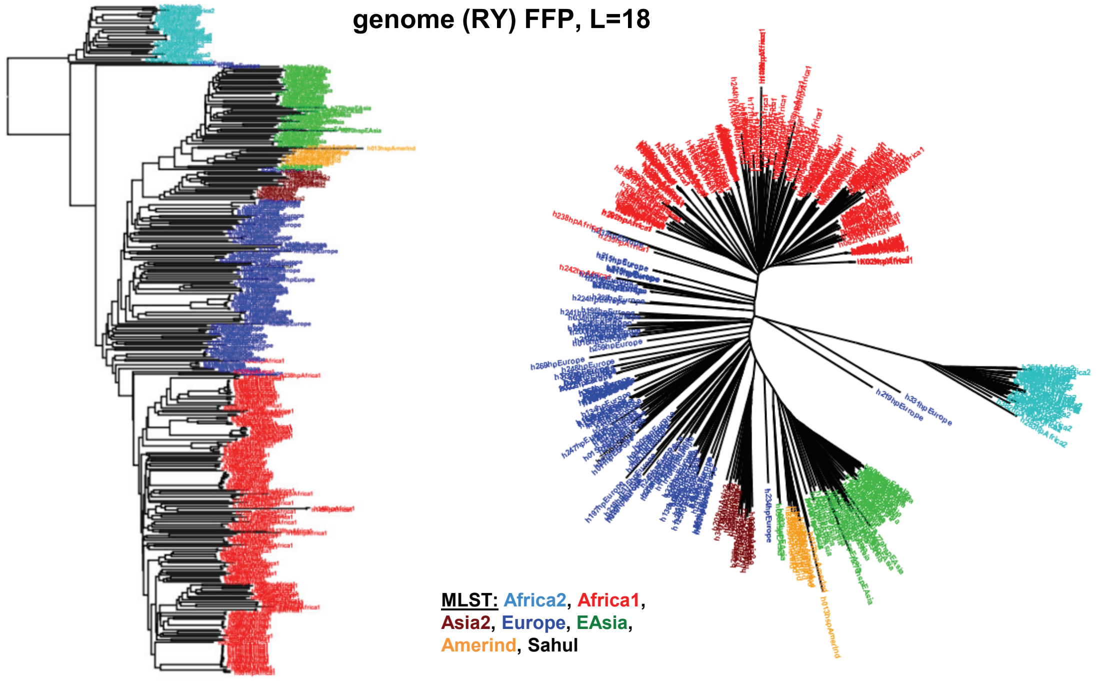

B

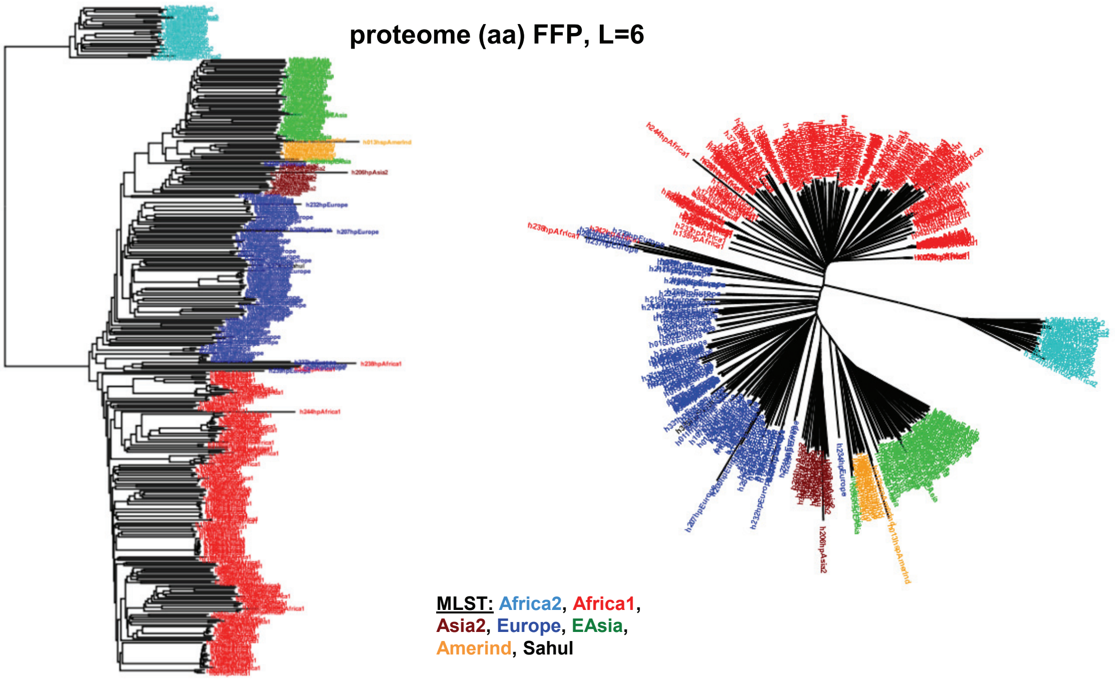

Figure S6. Untransformed trees in traditional (left) and radial (right) format of the (A) whole genome FFPry phylogenetic tree and (B) the whole proteome FFPaa tree used in Figures 4 and S7. The sample codes are colored according to the MLST-typing scheme. Trees are rooted using *H. acinonychis* strain Sheeba (Table S1) as outgroup.

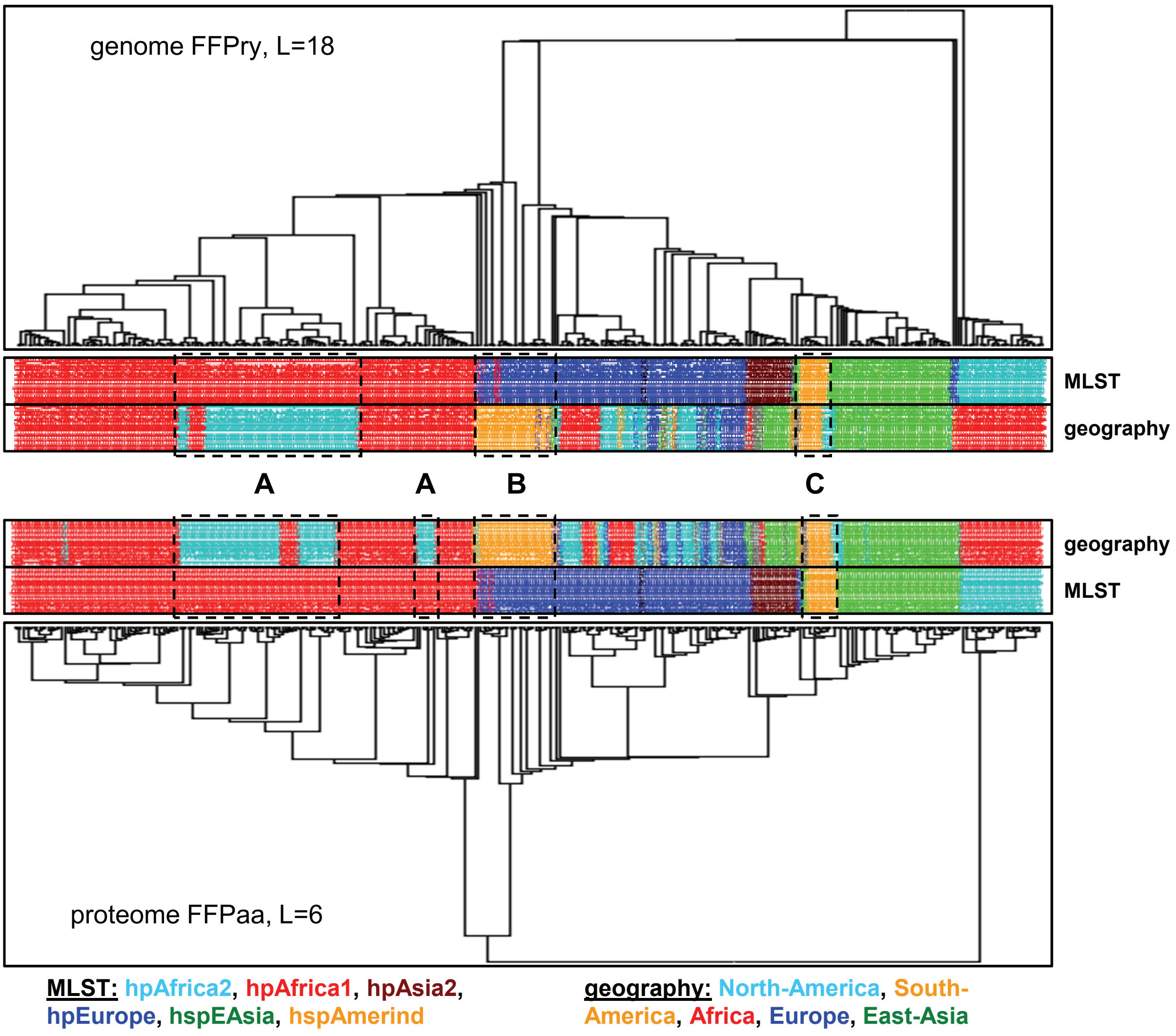

Figure S7. Feature frequency profiling of whole genomes (top) and whole proteomes (bottom) matches phylogeographic-based molecular epidemiology of *H. pylori*. A total of 377 *H. pylori* genome sequences and proteomes derived from annotated genome sequences (Table S2) were compared to MLST-derived genotypes and the (sub)continent where the strain was isolated. The trees have been transformed using the "proportional" setting of Figtree 1.4.0 for presentational purposes, the non-transformed trees with the original branch lengths are shown in Figure S6. The boxed areas show (A) a subgroup of hpAfrica1 strains isolated in North-America, probably representing migrations from the 17th century onwards (5, 6), and (B,C) the two separate clusters of *H. pylori* strains from South- and Middle-America (indicated as "South-America"), which represent the hspAmerind group from early human migration (C) and the hpEurope/hpAfrica1 strains from recent migrations (B). Trees are rooted using *H. acinonychis* Sheeba genome/proteome (Table S1) as outgroup.

Table S1. Overview of genome sequences of gastric *Helicobacter* species used for Figure 1.

Species	Strain	Isolated from	Accession number	MLST-type
H. acinonychis	Sheeba	lion	NC_008229	n/a
H. bizzozeronii	CCUG 35545	dog	CAGP01	n/a
H. bizzozeronii	CIII-1	human	NC_015674	n/a
H. cetorum	MIT 99-5656	dolphin	CP003481	n/a
H. cetorum	MIT 00-7128	whale	CP003479	n/a
H. felis	ATCC 49179	cat	NC_014810	n/a
H. heilmannii	ASB1.4	cat	HE984298	n/a
H. mustelae	12198	ferret	NC_013949	n/a
H. pylori	26695	human	NC_000915	hpEurope
H. pylori	Cuz20	human	CP002076	hspAmerind
H. pylori	India7	human	CP002331	hpAsia2
H. pylori	J99	human	NC_000921	hpAfrica1
H. pylori	OK113	human	AP012600	hspEAsia
H. pylori	SouthAfrica7	human	CP002336	hpAfrica2
H. suis	HS1	pig	ADGY01	n/a
H. suis	HS5	pig	ADHO01	n/a

Table S2. Overview of *H. pylori* genome sequences included in this study. Accession number, isolate designation, MLST-type, continent/subcontinent of isolation and virulence marker genotypes are indicated.

[a]Genome number labeled **blue** are included in Figure 2

[b] Continent/subcontinent where strain was isolated

[c]as determined by in silico PCR using MIST, with primers listed in Table S3

[d] PZ1/PZ2 positivity based on jhp0945/0947/0949-positivity for PZ1 and jhp0940-positivity for PZ2

Isolate	number[a]	Accession number	geography[b]	MLST	disease / species	cag PAI [c]	vacA s-m [c]	vacA i-d [c]	babA2[c]	dupA[c]	jhp917-918 [c]	iceA1-A2 [c]	PZ1/PZ2 [c,d]
2017	**h001**	CP002571	Africa	hpAfrica1	unknown	cag+	s1-m1	i1-d1	babA2+	short	917+918	only iceA2	neither
2018	**h002**	CP002572	Africa	hpAfrica1	unknown	cag+	s1-m1	i1-d1	babA2+	short	917+918	only iceA2	neither
26695	**h003**	CP002096	Europe	hpEurope	gastritis	cag+	s1-m1	i1-d1	no babA2	absent	neither	iceA1 only	PZ2 only
35A	**h004**	CP002096	North America	hspEAsia	unknown	cag+	s1-m1	i1-d1	babA2+	absent	neither	only iceA2	neither
51	**h005**	CP000012	East Asia	hspEAsia	peptic ulcer	cag+	s1-m1	i1-d1	babA2+	long	neither	iceA1 only	neither
52	**h006**	CP001680	East Asia	hspEAsia	unknown	cag+	s1-m1	i1-d1	babA2+	absent	neither	iceA1 only	neither
83	**h007**	CP002605	North America	hspEAsia	unknown	cag+	s1-m1	i1-d1	babA2+	long	neither	iceA1 only	PZ1+PZ2
8A3	h008	CADD01	North America	hspEAsia	unknown	cag-neg	s1-m1	i1-d1	babA2+	absent	neither	iceA1+A2	PZ1 only
908	**h009**	CP002184	Africa	hpAfrica1	unknown	cag+	s1-?	i1-d1	babA2+	short	917+918	only iceA2	neither
98-10	h010	ABSX01	East Asia	hspEAsia	gastric cancer	cag+	s1-m1	i1-d1	babA2+	long	only 918	iceA1 only	neither
A45	h011	AMYU01	Europe	hpEurope	peptic ulcer	cag-neg	s2-m2	i2-d2	no babA2	long	917+918	only iceA2	neither
Aklavik117	**h012**	CP003483	North America	hspAmerInd	gastritis	cag-neg	s2-m2	i2-d2	babA2+	long	917+918	iceA1 only	neither
Aklavik86	**h013**	CP003476	North America	hspAmerInd	gastritis	cag-neg	s1-m1	i1-d2	no babA2	absent	neither	only iceA2	neither
B128	h014	ABSY01	Europe	hpEurope	peptic ulcer	cag+	s1-m2	i1-d2	no babA2	absent	neither	iceA1 only	PZ1+PZ2
B38	**h015**	NC_012973	Europe	hpEurope	peptic ulcer	cag-neg	s2-m2	i2-d2	babA2+	absent	neither	iceA1 only	PZ1 only
B45	h016	AFAO01	Europe	hpEurope	unknown	cag+	s1-m1	i2-d2	no babA2	absent	only 918	iceA1 only	PZ1 only
B8	**h017**	NC_014256	Europe	hpEurope	peptic ulcer	cag+	s1-m2	i1-d2	babA2+	absent	neither	iceA1 only	PZ1+PZ2
BCS100H1	h018	CADC01	North America	hspEAsia	unknown	cag-neg	s1-m1	i1-d1	babA2+	absent	neither	iceA1+A2	PZ1 only
BM012A	**h019**	CP006888	(Other) Australia	hpEurope	unknown	cag+	s1-?	i2-d2	babA2+	absent	neither	iceA1 only	PZ1+PZ2
BM012S	**h020**	CP006889	(Other) Australia	hpEurope	unknown	cag+	s1-?	i2-d2	babA2+	absent	neither	iceA1 only	PZ1+PZ2
CCHI_33	**h021**	AOTU01	North America	hpAfrica1	unknown	cag+	s1-m1	i1-d1	babA2+	short	917+918	only iceA2	PZ1 only
CG-IMSS-2012	h022	AWUL01	South America	hpEurope	gastric cancer	cag+	s1-m1	i1-d1	babA2+	absent	only 918	iceA1+A2	PZ1+PZ2
CPY1124	h023	AKNJ01	East Asia	hspEAsia	peptic ulcer	cag+	s1-m1	i1-d1	babA2+	absent	only 918	iceA1+A2	neither
CPY1313	h024	AKNK01	East Asia	hspEAsia	peptic ulcer	cag+	s1-m1	i1-d1	babA2+	absent	only 918	iceA1 only	PZ1+PZ2
CPY1662	h025	AOTT01	East Asia	hspEAsia	unknown	cag+	s1-m2	i1-d1	babA2+	absent	only 918	iceA1 only	PZ1 only
CPY1962	h026	AKNL01	East Asia	hspEAsia	peptic ulcer	cag+	s1-m1	i1-d1	babA2+	short	neither	iceA1 only	PZ2 only
CPY3281	h027	AKNM01	East Asia	hspEAsia	peptic ulcer	cag+	s1-m1	i1-d1	babA2+	long	917+918	iceA1+A2	PZ1 only
CPY6081	h028	AKNN01	East Asia	hspEAsia	gastric cancer	cag+	s1-m1	i1-d1	babA2+	long	917+918	iceA1+A2	PZ1 only
CPY6261	h029	AKNO01	East Asia	hspEAsia	gastric cancer	cag+	s1-m1	i1-d1	babA2+	long	neither	iceA1 only	PZ1+PZ2
CPY6271	h030	AKNP01	East Asia	hspEAsia	gastric cancer	cag+	s1-m1	i1-d1	babA2+	long	917+918	iceA1+A2	PZ1 only
CPY6311	h031	AKNQ01	East Asia	hspEAsia	gastric cancer	cag+	s1-m1	i1-d1	babA2+	absent	neither	iceA1+A2	neither
Cuz20	**h032**	CP002076	South America	hspAmerInd	unknown	cag+	s1-m1	i2-d2	babA2+	long	917+918	only iceA2	neither
E48	h033	AYHQ01	(Other) Russia	hspEAsia	gastritis	cag+	s1-m1	i1-d1	babA2+	long	917+918	iceA1 only	neither
ELS37	**h034**	CP002953	South America	hpEurope	gastric cancer	cag+	s1-m1	i1-d1	babA2+	absent	neither	iceA1 only	PZ2 only
F16	**h035**	AP011940	East Asia	hspEAsia	unknown	cag+	s1-m1	i1-d1	babA2+	absent	neither	iceA1 only	neither
F30	**h036**	AP011941	East Asia	hspEAsia	unknown	cag+	s1-m1	i1-d1	babA2+	long	neither	iceA1 only	neither
F32	**h037**	AP011943	East Asia	hspEAsia	unknown	cag+	s1-m1	i1-d1	babA2+	absent	neither	iceA1 only	PZ2 only
F57	**h038**	AP011945	East Asia	hspEAsia	unknown	cag+	s1-m1	i1-d1	babA2+	long	917+918	iceA1 only	PZ1 only
FD423	**h039**	AKHM02	East Asia	hpAsia2	dyspepsia	cag+	s1-?	i1-d1	babA2+	long	neither	iceA1 only	PZ1 only
FD430	h040	AKHN02	East Asia	hpAsia2	dyspepsia	cag+	?-m1	i1-d1	babA2+	absent	neither	iceA1+A2	PZ1 only
FD506	h041	AKHO02	East Asia	hspEAsia	dyspepsia	cag+	?-?	i1-d1	babA2+	short	only 918	iceA1 only	PZ1+PZ2
FD535	h042	AKHP02	East Asia	hpAsia2	dyspepsia	cag+	s1-m1	i1-d1	babA2+	long	917+918	iceA1 only	PZ1 only
FD568	h043	AKHQ02	East Asia	hspEAsia	dyspepsia	cag+	?-m1	i1-d1	no babA2	long	917+918	iceA1 only	PZ1 only
FD577	h044	AKHR02	East Asia	hspEAsia	dyspepsia	cag+	s1-m1	i1-d1	babA2+	short	only 918	iceA1 only	PZ1+PZ2

FD662	h045	AKHT02	East Asia	hpAsia2	dyspepsia	cag+	?-m1	i1-d1	babA2+	absent	neither	only iceA2	PZ2 only
FD703	h046	AKHS02	East Asia	hpAsia2	dyspepsia	cag+	?-m1	i1-d1	babA2+	absent	neither	iceA1 only	PZ1+PZ2
FD719	h047	AKHU02	East Asia	hpAsia2	dyspepsia	cag+	?-m1	i1-d1	babA2+	absent	neither	iceA1+A2	PZ1 only
G27	**h048**	NC_011333	Europe	hpEurope	unknown	cag+	s1-m1	i1-d1	babA2+	long	917+918	only iceA2	neither
GAM100Ai	h049	ANFP01	Africa	hpAfrica1	unknown	cag+	s1-m1	i1-d1	babA2+	short	917+918	neither	PZ1+PZ2
GAM101Biv	h050	APCW01	Africa	hpAfrica1	unknown	cag-neg	s2-m2	i2-d2	no babA2	long	917+918	iceA1+A2	PZ2 only
GAM103Bi	**h051**	APCX01	Africa	hpAfrica1	unknown	cag+	s1-m1	i1-d1	babA2+	short	917+918	iceA1+A2	PZ1 only
GAM105Ai	h052	APCY01	Africa	hpAfrica1	unknown	cag+	s1-m1	i1-d1	babA2+	absent	neither	only iceA2	PZ2 only
GAM112Ai	h053	APCZ01	Africa	hpAfrica1	unknown	cag+	s1-m1	i1-d1	babA2+	long	917+918	only iceA2	PZ1 only
GAM114Ai	h054	APDA01	Africa	hpAfrica1	unknown	cag+	s1-m1	i1-d1	babA2+	absent	only 918	only iceA2	PZ2 only
GAM115Ai	h055	APDB01	Africa	hpAfrica1	unknown	cag+	s1-m2	i1-d1	no babA2	short	917+918	only iceA2	PZ1+PZ2
GAM117Ai	h056	AWER01	Africa	hpAfrica1	unknown	cag+	s1-m1	i1-d1	babA2+	short	917 only	only iceA2	PZ2 only
GAM118Bi	h057	APDD01	Africa	hpAfrica1	unknown	cag+	s1-m2	i2-d1	babA2+	long	917+918	only iceA2	PZ1+PZ2
GAM119Bi	h058	APDE01	Africa	hpAfrica1	unknown	cag+	s1-m1	i1-d1	babA2+	short	917+918	iceA1 only	PZ1+PZ2
GAM120Ai	h059	APDF01	Africa	hpAfrica1	unknown	cag+	s1-m1	i1-d1	babA2+	short	917+918	only iceA2	PZ1+PZ2
GAM121Aii	h060	APDG01	Africa	hpAfrica1	unknown	cag+	s1-m1	i1-d1	babA2+	short	917+918	only iceA2	PZ1+PZ2
GAM201Ai	h061	APDC01	Africa	hpAfrica1	unknown	cag-neg	s2-m2	i2-d2	no babA2	long	917+918	iceA1+A2	neither
GAM210Bi	h062	APDH01	Africa	hpAfrica1	unknown	cag+	s1-m1	i1-d1	babA2+	short	917+918	iceA1+A2	PZ1 only
GAM231Ai	h063	APDI01	Africa	hpAfrica1	unknown	cag-neg	s2-m2	i2-d2	no babA2	long	917+918	iceA1 only	PZ1+PZ2
GAM239Bi	h064	APDJ01	Africa	hpAfrica1	unknown	cag-neg	s2-m2	i2-d2	no babA2	short	917+918	iceA1 only	PZ2 only
GAM244Ai	h065	APDK01	Africa	hpAfrica1	unknown	cag-neg	s2-m2	i2-d2	no babA2	absent	only 918	only iceA2	PZ1+PZ2
GAM245Ai	h066	APDL01	Africa	hpAfrica1	unknown	cag+	?-m1	i1-d1	babA2+	absent	neither	only iceA2	PZ1+PZ2
GAM246Ai	h067	APDM01	Africa	hpAfrica1	unknown	cag+	s1-m1	i1-d1	babA2+	short	917+918	only iceA2	PZ1+PZ2
GAM249T	h068	APDN01	Africa	hpAfrica1	unknown	cag+	s1-m1	i1-d1	babA2+	short	917+918	only iceA2	PZ1 only
GAM250AFi	h069	APDO01	Africa	hpAfrica1	unknown	cag+	s1-m1	i1-d1	babA2+	absent	only 918	iceA1+A2	neither
GAM250T	h070	APDP01	Africa	hpAfrica1	unknown	cag+	s1-m1	i1-d1	no babA2	absent	only 918	iceA1+A2	neither
GAM252Bi	h071	APDQ01	Africa	hpAfrica1	unknown	cag+	s1-m1	i1-d1	babA2+	absent	only 918	iceA1+A2	neither
GAM252T	h072	APDR01	Africa	hpAfrica1	unknown	cag+	s1-m1	i1-d1	babA2+	absent	only 918	iceA1+A2	neither
GAM254Ai	h073	APDS01	Africa	hpAfrica1	unknown	cag+	s1-m2	i2-d2	babA2+	long	917+918	only iceA2	PZ1 only
GAM260ASi	h074	APDT01	Africa	hpAfrica1	unknown	cag+	s1-m1	i1-d1	babA2+	short	917+918	iceA1 only	PZ1 only
GAM260Bi	h075	APDU01	Africa	hpAfrica1	unknown	cag+	s1-m1	i1-d1	babA2+	short	917+918	only iceA2	PZ1+PZ2
GAM260BSi	**h076**	APDV01	Africa	hpAfrica1	unknown	cag-neg	s2-m2	i2-d2	no babA2	long	917+918	iceA1+A2	neither
GAM263BFi	h077	APDW01	Africa	hpAfrica1	unknown	cag+	s1-m1	i1-d1	babA2+	long	917+918	only iceA2	PZ1+PZ2
GAM264Ai	h078	APDX01	Africa	hpAfrica1	unknown	cag-neg	s2-m2	i2-d2	no babA2	absent	only 918	iceA1+A2	PZ1+PZ2
GAM265BSii	h079	APDY01	Africa	hpAfrica1	unknown	cag+	s1-m2	i2-d1	no babA2	short	917+918	iceA1 only	PZ1 only
GAM268Bii	h080	APDZ01	Africa	hpAfrica1	unknown	cag+	s1-m1	i1-d1	babA2+	short	917+918	iceA1 only	PZ1 only
GAM270ASi	h081	APEA01	Africa	hpAfrica1	unknown	cag+	s1-m1	i1-d1	babA2+	absent	neither	only iceA2	PZ1+PZ2
GAM42Ai	h082	APEB01	Africa	hpAfrica1	unknown	cag+	s1-m1	i1-d1	no babA2	long	917+918	only iceA2	PZ1 only
GAM71Ai	h083	APEC01	Africa	hpAfrica1	unknown	cag-neg	s2-m2	i2-d2	babA2+	long	917+918	iceA1+A2	PZ2 only
GAM80Ai	h084	APED01	Africa	hpAfrica1	unknown	cag+	s1-m1	i1-d1	babA2+	absent	neither	only iceA2	PZ1 only
GAM83Bi	h085	APEE01	Africa	hpAfrica1	unknown	cag-neg	s2-m2	i2-d2	babA2+	long	917+918	iceA1+A2	PZ1 only
GAM83T	h086	APEF01	Africa	hpAfrica1	unknown	cag-neg	s2-m2	i2-d2	babA2+	long	917+918	iceA1+A2	PZ1 only
GAM93Bi	h087	APEG01	Africa	hpAfrica1	unknown	cag+	s1-m1	i1-d1	no babA2	short	917+918	iceA1+A2	PZ1 only
GAM96Ai	h088	APEH01	Africa	hpAfrica1	unknown	cag+	?-m2	i2-d2	babA2+	absent	neither	only iceA2	neither
Gambia9424	**h089**	CP002332	Africa	hpAfrica1	unknown	cag+	s1-m1	i1-d1	babA2+	short	917+918	only iceA2	PZ1+PZ2
GAMchJs106B	h090	APEI01	Africa	hpAfrica1	unknown	cag-neg	s2-m2	i2-d2	babA2+	absent	only 918	iceA1 only	PZ1+PZ2
GAMchJs114i	h091	APEJ01	Africa	hpAfrica1	unknown	cag-neg	s2-m2	i2-d2	no babA2	absent	only 918	only iceA2	PZ1+PZ2
GAMchJs117Ai	h092	APEK01	Africa	hpAfrica1	unknown	cag-neg	s2-m2	i2-d2	babA2+	long	917+918	iceA1 only	PZ1+PZ2
GAMchJs124i	h093	APEL01	Africa	hpAfrica1	unknown	cag-neg	s2-m2	i2-d2	no babA2	long	917+918	iceA1 only	PZ1+PZ2
GAMchJs136i	h094	APEM01	Africa	hpAfrica1	unknown	cag+	s1-m1	i1-d1	babA2+	short	917+918	only iceA2	PZ1+PZ2
GC26	h095	AKHV02	East Asia	hspEAsia	gastric cancer	cag+	s1-m1	i1-d1	no babA2	long	917+918	iceA1 only	PZ1 only

H13-1	h096	AYUH01	(Other) Russia	hspEAsia	gastritis	cag+	s1-m1	i1-d1	babA2+	absent	only 918	only iceA2	PZ1+PZ2
HLJHP193	h097	ALJI01	East Asia	hspEAsia	peptic ulcer	cag+	s1-m1	i1-d1	babA2+	long	only 918	iceA1 only	PZ1 only
HLJHP253	h098	ALKC01	East Asia	hspEAsia	peptic ulcer	cag+	s1-m2	i1-d1	babA2+	short	neither	iceA1 only	neither
HLJHP256	h099	ALKA01	East Asia	hspEAsia	peptic ulcer	cag+	s1-m2	i1-d1	babA2+	short	neither	iceA1 only	neither
HLJHP271	h100	ALKB01	East Asia	hspEAsia	peptic ulcer	cag+	s1-m2	i1-d1	babA2+	short	neither	iceA1 only	neither
Hp_A-11	h101	AOTW01	North America	hpEurope	peptic ulcer	cag+	s1-m2	i1-d1	babA2+	long	917+918	iceA1 only	PZ1+PZ2
Hp_A-14	h102	AKOT01	North America	hpEurope	gastritis	cag-neg	s2-m2	i2-d2	babA2+	absent	only 918	iceA1 only	PZ1 only
Hp_A-16	**h103**	AKOU01	North America	hpAfrica1	gastritis	cag+	s1-m1	i1-d1	babA2+	short	917+918	only iceA2	PZ1 only
Hp_A-17	h104	AKOD01	North America	hpAfrica1	peptic ulcer	cag-neg	s1-m1	i1-d1	babA2+	short	917+918	only iceA2	PZ1+PZ2
Hp_A-20	h105	AKOE01	North America	hpAfrica1	peptic ulcer	cag+	s1-m1	i1-d1	babA2+	short	917+918	iceA1 only	PZ1 only
Hp_A-26	h106	AKOV01	North America	hpEurope	gastritis	cag-neg	s2-m2	i2-d2	no babA2	absent	neither	iceA1 only	PZ1+PZ2
Hp_A-27	h107	AKOW01	North America	hpEurope	gastritis	cag+	s1-m2	i1-d?	babA2+	long	917+918	iceA1 only	PZ1+PZ2
Hp_A-4	h108	AKOA01	North America	hpAfrica1	peptic ulcer	cag+	s1-m2	i1-d1	babA2+	short	917+918	iceA1 only	neither
Hp_A-5	h109	AKOB01	North America	hpAfrica1	peptic ulcer	cag+	s1-m1	i1-d1	babA2+	long	neither	iceA1+A2	PZ1+PZ2
Hp_A-6	h110	AKOR01	North America	hpAfrica1	gastritis	cag+	s1-m1	i1-d1	babA2+	short	917+918	only iceA2	PZ1+PZ2
Hp_A-8	h111	AKOS01	North America	hpAfrica1	gastritis	cag+	s1-m1	i1-d1	babA2+	short	917+918	only iceA2	PZ1 only
Hp_A-9	h112	AKOC01	North America	hpEurope	peptic ulcer	cag+	s1-m2	i2-d2	babA2+	long	917+918	only iceA2	PZ2 only
Hp_H-1	h113	AOTX01	North America	hpAfrica1	gastritis	cag+	s1-m1	i1-d1	babA2+	short	917+918	only iceA2	PZ1 only
Hp_H-10	h114	AKPB01	North America	hpAfrica1	gastritis	cag+	s1-m1	i1-d1	babA2+	short	917 only	only iceA2	PZ1 only
Hp_H-11	h115	AKPC01	North America	hpEurope	gastritis	cag+	s1-m2	i2-d2	no babA2	absent	neither	only iceA2	PZ2 only
Hp_H-16	h116	AKOF01	North America	hpAfrica1	peptic ulcer	cag+	s1-m1	i1-d1	babA2+	absent	neither	only iceA2	PZ1+PZ2
Hp_H-18	h117	AKPD01	North America	hpAfrica1	gastritis	cag+	s1-m1	i1-d1	babA2+	short	917+918	only iceA2	PZ1+PZ2
Hp_H-19	h118	AKPE01	North America	hpAfrica1	gastritis	cag+	s1-m1	i1-d1	babA2+	short	917 only	only iceA2	PZ1 only
Hp_H-21	h119	AKPF01	North America	hpAfrica1	gastritis	cag-neg	s1-m1	i1-d1	babA2+	short	917+918	only iceA2	PZ1+PZ2
Hp_H-23	h120	AKPG01	North America	hpAfrica1	gastritis	cag+	s1-m1	i1-d1	babA2+	long	917+918	only iceA2	PZ1 only
Hp_H-24	h121	AKOG01	North America	hpAfrica1	peptic ulcer	cag+	s1-m1	i1-d1	babA2+	short	917+918	iceA1 only	PZ1 only
Hp_H-24b	h122	AKPY01	North America	hpAfrica1	gastritis	cag+	s1-m1	i1-d1	no babA2	short	917+918	iceA1 only	PZ1 only
Hp_H-24c	h123	AKPZ01	North America	hpAfrica1	gastritis	cag+	s1-m1	i1-d1	babA2+	short	917+918	iceA1 only	PZ1 only
Hp_H-27	h124	AKOH01	North America	hpEurope	peptic ulcer	cag-neg	s2-m2	i2-d2	no babA2	long	neither	only iceA2	PZ1+PZ2
Hp_H-28	h125	AKOI01	North America	hpEurope	peptic ulcer	cag+	s1-m1	i1-d1	babA2+	absent	neither	iceA1+A2	neither
Hp_H-29	h126	AKOJ01	North America	hpAfrica1	peptic ulcer	cag+	s1-m1	i1-d1	babA2+	short	917+918	neither	PZ1+PZ2
Hp_H-3	h127	AKOX01	North America	hpAfrica1	gastritis	cag+	s1-m1	i1-d1	babA2+	long	917+918	only iceA2	PZ1+PZ2
Hp_H-30	h128	AKOK01	North America	hpAfrica1	peptic ulcer	cag+	s1-m1	i1-d1	babA2+	short	917+918	only iceA2	PZ1 only
Hp_H-34	**h129**	AKPH01	North America	hpAfrica1	gastritis	cag-neg	s1-m1	i1-d1	babA2+	short	917+918	iceA1+A2	PZ2 only
Hp_H-36	h130	AKOL01	North America	hpAfrica1	peptic ulcer	cag+	s1-m1	i1-d1	babA2+	short	neither	only iceA2	PZ1+PZ2
Hp_H-4	h131	AKOY01	North America	hpAfrica1	gastritis	cag+	s1-m1	i1-d1	babA2+	short	neither	iceA1 only	PZ1+PZ2
Hp_H-41	h132	AKOM01	North America	hpAfrica1	peptic ulcer	cag+	s1-m1	i1-d1	babA2+	short	917+918	only iceA2	PZ1 only
Hp_H-42	h133	AKON01	North America	hpAfrica1	peptic ulcer	cag+	s1-m1	i1-d1	babA2+	short	917+918	only iceA2	PZ1+PZ2
Hp_H-43	h134	AKOO01	North America	hpEurope	peptic ulcer	cag-neg	s2-m2	i2-d2	no babA2	absent	neither	iceA1+A2	PZ1+PZ2
Hp_H-44	h135	AKOP01	North America	hpAfrica1	peptic ulcer	cag+	s1-m1	i1-d1	babA2+	short	917+918	iceA1 only	PZ1+PZ2
Hp_H-45	h136	AKOQ01	North America	hpEurope	peptic ulcer	cag+	s1-m1	i1-d1	babA2+	absent	neither	iceA1 only	PZ1 only
Hp_H-5b	h137	AKQF01	North America	hpAfrica1	gastritis	cag+	s1-m1	i1-d1	babA2+	short	917+918	only iceA2	PZ2 only
Hp_H-6	h138	AKOZ01	North America	hpAfrica1	gastritis	cag+	s1-m2	i1-d1	babA2+	short	917+918	only iceA2	PZ1+PZ2
Hp_H-9	h139	AKPA01	North America	hpEurope	gastritis	cag+	s1-m1	i1-d1	babA2+	long	917+918	iceA1 only	PZ1+PZ2
Hp_M1	h140	AKQN01	North America	hpAfrica1	mouse	cag+	s1-m1	i1-d1	babA2+	short	917+918	iceA1 only	PZ1 only
Hp_M2	h141	AKQO01	North America	hpAfrica1	mouse	cag+	s1-m1	i1-d1	babA2+	short	917+918	iceA1 only	PZ1 only
Hp_M3	h142	AKQP01	North America	hpAfrica1	mouse	cag+	s1-m1	i1-d1	babA2+	short	917+918	iceA1 only	PZ1 only
Hp_M4	h143	AKQQ01	North America	hpAfrica1	mouse	cag+	s1-m1	i1-d1	babA2+	short	917+918	iceA1 only	PZ1 only
Hp_M5	h144	AKQR01	North America	hpAfrica1	mouse	cag+	s1-m1	i1-d1	babA2+	short	917+918	iceA1 only	PZ1 only
Hp_M6	h145	AKQS01	North America	hpAfrica1	mouse	cag+	s1-m1	i1-d1	babA2+	short	917+918	iceA1 only	PZ1 only
Hp_M9	**h146**	AKQT01	North America	hpAfrica1	mouse	cag+	s1-m1	i1-d1	babA2+	short	917+918	iceA1 only	PZ1 only

Hp_P-1	h147	AKPI01	North America	hpAfrica1	gastritis	cag+	s1-m1	i1-d1	babA2+	short	917+918	only iceA2	PZ1+PZ2
Hp_P-11	h148	AKPN01	North America	hpAfrica1	gastritis	cag+	s1-m1	i1-d1	no babA2	long	917+918	only iceA2	PZ1+PZ2
Hp_P-11b	h149	AKQH01	North America	hpAfrica1	gastritis	cag+	s1-m1	i1-d1	no babA2	long	917+918	only iceA2	PZ1+PZ2
Hp_P-13	h150	AKPO01	North America	hpAfrica1	gastritis	cag+	s1-m1	i1-d1	babA2+	long	917+918	only iceA2	PZ1+PZ2
Hp_P-13b	h151	AKQI01	North America	hpAfrica1	gastritis	cag+	s1-m1	i1-d1	babA2+	long	917+918	only iceA2	PZ1+PZ2
Hp_P-15	h152	AKPP01	North America	hpEurope	gastritis	cag-neg	s2-m2	i2-d2	no babA2	absent	neither	only iceA2	PZ1+PZ2
Hp_P-15b	h153	AKQJ01	North America	hpEurope	gastritis	cag-neg	s2-m2	i2-d2	no babA2	absent	neither	only iceA2	PZ1+PZ2
Hp_P-16	h154	AKPQ01	North America	hpEurope	gastritis	cag-neg	s2-m2	i2-d2	no babA2	absent	neither	iceA1 only	PZ2 only
Hp_P-1b	h155	AKQA01	North America	hpAfrica1	gastritis	cag+	s1-m1	i1-d1	babA2+	short	917+918	only iceA2	PZ1+PZ2
Hp_P-2	h156	AKPJ01	North America	hpAfrica1	gastritis	cag+	s1-m1	i1-d1	babA2+	long	917+918	only iceA2	PZ1 only
Hp_P-23	h157	AKPR01	North America	hpEurope	gastritis	cag-neg	s2-m2	i2-d2	no babA2	absent	neither	only iceA2	PZ1+PZ2
Hp_P-25	h158	AKPS01	North America	hpAfrica1	gastritis	cag+	s1-?	i1-d1	babA2+	short	917+918	only iceA2	PZ1 only
Hp_P-25c	h159	AKQK01	North America	hpAfrica1	gastritis	cag+	s1-m1	i1-d1	babA2+	short	917+918	only iceA2	PZ1 only
Hp_P-25d	h160	AKQL01	North America	hpAfrica1	gastritis	cag+	s1-m1	i1-d1	babA2+	short	917+918	only iceA2	PZ1 only
Hp_P-26	h161	AKPT01	North America	hpAfrica1	gastritis	cag+	s1-m1	i1-d1	babA2+	long	917+918	only iceA2	PZ1+PZ2
Hp_P-28b	h162	AKQM01	North America	hpAfrica1	gastritis	cag-neg	s1-m1	i1-d1	babA2+	long	917+918	only iceA2	PZ2 only
Hp_P-2b	h163	AKQB01	North America	hpAfrica1	gastritis	cag+	s1-m1	i1-d1	babA2+	long	917+918	only iceA2	PZ1 only
Hp_P-3	h164	AKPK01	North America	hpAfrica1	gastritis	cag+	s1-m1	i1-d1	no babA2	short	917+918	only iceA2	PZ1 only
Hp_P-30	h165	AKPU01	North America	hpEurope	gastritis	cag-neg	s2-m2	i2-d2	babA2+	long	917+918	only iceA2	PZ2 only
Hp_P-3b	h166	AKQC01	North America	hpAfrica1	gastritis	cag+	s1-m1	i1-d1	babA2+	short	917+918	only iceA2	PZ1 only
Hp_P-4	h167	AKPL01	North America	hpAfrica1	gastritis	cag+	s1-m1	i1-d1	babA2+	short	917+918	only iceA2	PZ1 only
Hp_P-41	h168	AKPV01	North America	hpAfrica1	gastritis	cag+	?-m1	i1-d1	babA2+	short	917+918	iceA1+A2	PZ1+PZ2
Hp_P-4c	h169	AKQD01	North America	hpAfrica1	gastritis	cag+	s1-m1	i1-d1	babA2+	short	917+918	only iceA2	PZ1 only
Hp_P-4d	h170	AKQE01	North America	hpAfrica1	gastritis	cag+	s1-m1	i1-d1	babA2+	short	917+918	only iceA2	PZ1 only
Hp_P-62	h171	AKPW01	North America	hpAfrica1	gastritis	cag+	s1-m1	i1-d1	babA2+	short	917+918	only iceA2	PZ1+PZ2
Hp_P-74	h172	AKPX01	North America	hpEurope	gastritis	cag-neg	s1-m2	i2-d2	no babA2	absent	neither	only iceA2	PZ1+PZ2
Hp_P-8	h173	AKPM01	North America	hpAfrica1	gastritis	cag-neg	s1-m1	i1-d1	babA2+	short	917+918	iceA1+A2	PZ1 only
Hp_P-8b	h174	AKQG01	North America	hpAfrica1	gastritis	cag-neg	s1-m1	i1-d1	babA2+	short	917+918	iceA1+A2	PZ1 only
HP116Bi	**h175**	APEN01	Africa	hpAfrica1	unknown	cag+	s1-m1	i1-d1	no babA2	short	917+918	only iceA2	PZ1+PZ2
HP250AFii	h176	APEO01	Africa	hpAfrica1	unknown	cag+	s1-m1	i1-d1	babA2+	absent	only 918	iceA1+A2	neither
HP250AFiii	h177	APEP01	Africa	hpAfrica1	unknown	cag+	s1-m1	i1-d1	babA2+	absent	only 918	iceA1+A2	neither
HP250AFiV	h178	APEQ01	Africa	hpAfrica1	unknown	cag+	s1-m1	i1-d1	babA2+	absent	only 918	iceA1+A2	neither
HP250ASi	h179	APER01	Africa	hpAfrica1	unknown	cag+	s1-m1	i1-d1	babA2+	absent	only 918	iceA1+A2	neither
HP250ASii	h180	APES01	Africa	hpAfrica1	unknown	cag+	s1-m1	i1-d1	no babA2	absent	only 918	iceA1+A2	neither
HP250BFi	h181	APET01	Africa	hpAfrica1	unknown	cag+	s1-m1	i1-d1	babA2+	absent	only 918	iceA1+A2	neither
HP250BFii	h182	APEU01	Africa	hpAfrica1	unknown	cag+	s1-m1	i1-d1	babA2+	absent	only 918	iceA1+A2	neither
HP250BFiii	h183	APEV01	Africa	hpAfrica1	unknown	cag+	s1-m1	i1-d1	babA2+	absent	only 918	iceA1+A2	neither
HP250BFiV	h184	APEW01	Africa	hpAfrica1	unknown	cag+	s1-m1	i1-d1	babA2+	absent	only 918	iceA1+A2	neither
HP250BSi	h185	APEX01	Africa	hpAfrica1	unknown	cag+	s1-m1	i1-d1	babA2+	absent	only 918	iceA1+A2	neither
HP260AFi	h186	APEY01	Africa	hpAfrica1	unknown	cag+	s1-m1	i1-d1	babA2+	short	917+918	iceA1 only	PZ1 only
HP260AFii	h187	APEZ01	Africa	hpAfrica1	unknown	cag+	s1-m1	i1-d1	babA2+	short	917+918	iceA1 only	PZ1 only
HP260ASii	h188	APFA01	Africa	hpAfrica1	unknown	cag+	s1-m1	i1-d1	babA2+	short	917+918	iceA1 only	PZ1 only
HP260BFii	h189	APFB01	Africa	hpAfrica1	unknown	cag-neg	s2-m2	i2-d2	babA2+	long	917+918	iceA1+A2	neither
HP260Bi	h190	APFC01	Africa	hpAfrica1	unknown	cag+	s1-m1	i1-d1	babA2+	short	917+918	only iceA2	PZ1+PZ2
HP87hu	h191	CBRI01	Europe	hpEurope	gerbil	cag+	s1-m1	i1-d1	no babA2	absent	neither	only iceA2	PZ2 only
HP87P7	h192	CBRJ01	Europe	hpEurope	gerbil	cag+	s1-m1	i1-d1	no babA2	long	917+918	only iceA2	PZ1+PZ2
HP87P7tlpDRI	h193	CBRL01	Europe	hpEurope	gerbil	cag+	s1-m1	i1-d1	no babA2	long	917+918	only iceA2	PZ1+PZ2
HP87tlpD	h194	CBRK01	Europe	hpEurope	gerbil	cag+	s1-m1	i1-d1	no babA2	long	917+918	only iceA2	PZ1+PZ2
HPAG1	**h195**	NC_008086	Europe	hpEurope	atrophic gastritis	cag+	s1-m1	i1-d1	babA2+	absent	neither	iceA1 only	neither
HPARG63	h196	CBKY01	South America	hpEurope	unknown	cag+	s1-m1	i1-d1	babA2+	long	neither	iceA1 only	PZ1+PZ2
HPARG8G	h197	CBKZ01	South America	hpEurope	unknown	cag+	s1-m2	i1-d1	babA2+	absent	neither	iceA1 only	neither

HUP-B14	**h200**	CP003486	Europe	hpEurope	unknown	cag+	s1-m1	i1-d1	babA2+	absent	neither	only iceA2	neither
India7	**h201**	CP002331	(Other) India	hpAsia2	unknown	cag+	s1-m2	i2-d2	babA2+	absent	neither	only iceA2	neither
J99	**h202**	NC_000921	North America	hpAfrica1	peptic ulcer	cag+	s1-m1	i1-d1	babA2+	short	917+918	only iceA2	PZ1+PZ2
Lithuania75	**h203**	CP002334	Europe	hpEurope	unknown	cag+	s1-m1	i1-d1	babA2+	absent	neither	only iceA2	PZ2 only
MALT	h204	AOTV01	North America	hpEurope	MALT lymphoma	cag-neg	s2-m2	i2-d2	no babA2	absent	neither	iceA1+A2	neither
N6	h205	CAHX01	Europe	hpEurope	gastritis	cag+	s1-m2	i1-d1	no babA2	absent	neither	iceA1 only	PZ1+PZ2
NAB47	**h206**	AJFA02	(Other) India	hpAsia2	unknown	cag+	?-m1	i1-d?	babA2+	absent	neither	iceA1 only	PZ1 only
NAD1	h207	AJGJ02	(Other) India	hpEurope	unknown	cag+	?-m1	i1-d?	babA2+	absent	only 918	iceA1 only	PZ1 only
NAK7	h208	AONJ01	(Other) India	hpAsia2	unknown	cag+	s1-m1	i1-d1	babA2+	absent	neither	iceA1 only	neither
NCTC_11637	h209	AIHX01	(Other) Australia	hpEurope	gastritis	cag+	?-m1	i1-d1	babA2+	absent	only 918	iceA1+A2	neither
NQ1671	h210	CADM01	South America	hpEurope	unknown	cag+	s1-m1	i1-d1	babA2+	short	917+918	only iceA2	PZ1 only
NQ1701	h211	CADH01	South America	hpEurope	unknown	cag+	s1-m1	i1-d1	babA2+	short	917+918	iceA1 only	PZ1+PZ2
NQ1707	h212	CADJ01	South America	hpEurope	unknown	cag+	s1-m1	i1-d1	babA2+	short	917+918	only iceA2	PZ1+PZ2
NQ1712	h213	CADF01	South America	hpEurope	unknown	cag-neg	s1-m1	i1-d1	no babA2	long	917+918	iceA1+A2	neither
NQ315	h214	CADE01	South America	hpEurope	unknown	cag+	s1-m1	i1-d1	no babA2	long	917+918	iceA1 only	neither
NQ352	h215	CADG01	South America	hpEurope	unknown	cag+	s1-m1	i1-d1	babA2+	short	917+918	iceA1 only	PZ1+PZ2
NQ367	h216	CADL01	South America	hpEurope	unknown	cag+	s1-m1	i1-d1	babA2+	short	917+918	iceA1+A2	PZ1 only
NQ392	h217	CADI01	South America	hpEurope	unknown	cag+	s1-m1	i1-d1	no babA2	short	917+918	only iceA2	PZ1+PZ2
NQ4044	h218	AKNW01	South America	hpEurope	intestinal metaplasia	cag+	?-m1	i1-d1	babA2+	long	917+918	iceA1 only	PZ2 only
NQ4053	h219	AKNV01	South America	hpEurope	dysplasia	cag-neg	s1-m1	i1-d1	babA2+	long	917+918	iceA1 only	PZ1 only
NQ4060	h220	CADK01	South America	hpEurope	unknown	cag+	s1-m1	i1-d1	babA2+	short	917+918	only iceA2	PZ1+PZ2
NQ4076	h221	AKNX01	South America	hpEurope	atrophic gastritis	cag+	s1-m1	i1-d1	babA2+	absent	neither	iceA1+A2	PZ1+PZ2
NQ4099	h222	AKNU01	South America	hpEurope	dysplasia	cag+	s1-m1	i1-d1	babA2+	absent	neither	only iceA2	PZ2 only
NQ4110	h223	AKNZ01	South America	hpEurope	atrophic gastritis	cag-neg	s2-m2	i2-d2	no babA2	absent	neither	only iceA2	PZ1 only
NQ4161	h224	AKNY01	South America	hpEurope	atrophic gastritis	cag-neg	s1-m1	i1-d1	babA2+	absent	neither	only iceA2	PZ2 only
NQ4191	h225	CADN01	South America	hpEurope	unknown	cag+	s1-m1	i1-d1	babA2+	short	917+918	iceA1+A2	PZ1 only
NQ4200	h226	AKNS01	South America	hpEurope	intestinal metaplasia	cag+	s1-m1	i1-d1	babA2+	long	917+918	only iceA2	PZ2 only
NQ4216	h227	AKNR01	South America	hpEurope	dysplasia	cag+	s1-m1	i1-d1	babA2+	absent	neither	iceA1 only	neither
NQ4228	h228	AKNT01	South America	hpEurope	intestinal metaplasia	cag+	s1-m1	i1-d1	babA2+	short	917+918	only iceA2	PZ1 only
OK113	**h229**	AP012600	East Asia	hspEAsia	unknown	cag+	s1-m1	i1-d1	babA2+	absent	neither	iceA1 only	neither
OK310	**h230**	AP012601	East Asia	hspEAsia	unknown	cag+	s1-m1	i1-d1	babA2+	absent	neither	iceA1+A2	PZ2 only
P12	**h231**	NC_011498	Europe	hpEurope	peptic ulcer	cag+	s1-m1	i1-d1	babA2+	absent	neither	iceA1 only	PZ1+PZ2
P79	h232	AIHW01	Europe	hpEurope	unknown	cag+	?-m1	i1-d1	no babA2	absent	neither	iceA1 only	PZ1+PZ2
PeCan18	**h233**	CP003475	South America	hpAfrica1	gastritis	cag+	s1-m1	i1-d1	babA2+	absent	neither	only iceA2	PZ2 only
PeCan4	**h234**	NC_014555	South America	hpEurope	gastric cancer	cag+	s1-m1	i1-d1	babA2+	absent	neither	iceA1 only	PZ1 only
Puno120	**h235**	CP002980	South America	hspAmerInd	gastritis	cag+	s1-m1	i1-d1	no babA2	absent	neither	only iceA2	PZ2 only
Puno135	**h236**	CP002982	South America	hspAmerInd	gastritis	cag+	s1-m1	i1-d1	no babA2	long	917+918	iceA1 only	PZ2 only
PZ5004	h237	ASZF01	South America	hpEurope	unknown	cag+	s1-m1	i1-d?	no babA2	short	917+918	iceA1 only	neither
PZ5024	h238	ASYS01	South America	hpAfrica1	unknown	cag+	?-m1	i1-d1	babA2+	short	917+918	iceA1+A2	neither
PZ5026	h239	ASYT01	South America	hpEurope	unknown	cag+	s1-m1	i1-d1	babA2+	short	917+918	iceA1+A2	PZ1 only
PZ5056	h240	ASYU01	South America	hpEurope	unknown	cag+	s1-m1	i1-d1	no babA2	short	917+918	iceA1 only	PZ1 only
PZ5080	h241	ASYV01	South America	hpEurope	unknown	cag+	s1-m1	i1-d1	no babA2	short	neither	iceA1 only	PZ1+PZ2
PZ5086	h242	ASYW01	South America	hpAfrica1	unknown	cag+	?-m1	i2-?	babA2+	absent	only 918	iceA1 only	PZ1 only
R018c	h243	AMOQ01	North America	hpEurope	unknown	cag+	s1-m2	i1-d2	babA2+	absent	neither	only iceA2	PZ1+PZ2
R030b	**h244**	AMOR01	North America	hpAfrica1	unknown	cag+	s1-m1	i1-d1	no babA2	absent	neither	iceA1 only	PZ1 only
R036d	h245	AMOT01	North America	hpEurope	unknown	cag+	s2-m2	i2-d2	babA2+	absent	neither	only iceA2	PZ1+PZ2
R037c	h246	AMOU01	North America	hpEurope	unknown	cag-neg	s2-m2	i2-d2	babA2+	long	917+918	iceA1 only	PZ1+PZ2
R038b	h247	AMOV01	North America	hpEurope	unknown	cag-neg	s2-m2	i2-d2	no babA2	long	917+918	iceA1 only	neither
R046Wa	h248	AMOW01	North America	hpEurope	unknown	cag-neg	?-m1	i1-d1	babA2+	absent	neither	iceA1 only	PZ1+PZ2
R055a	h249	AMOX01	North America	hpEurope	unknown	cag+	s1-m2	i2-d2	babA2+	long	917+918	iceA1 only	PZ1 only
R056a	h250	AMOY01	North America	hpEurope	unknown	cag+	s1-m2	i1-d2	babA2+	absent	neither	only iceA2	PZ1+PZ2

R32b	h251	AMOS01	North America	hpEurope	unknown	cag+	s1-m1	i1-d1	babA2+	absent	neither	only iceA2	PZ1 only
Rif1	h252	CP003905	Europe	hpEurope	unknown	cag+	s1-m1	i1-d1	no babA2	absent	neither	iceA1 only	PZ2 only
Rif2	h253	CP003906	Europe	hpEurope	unknown	cag+	s1-m1	i1-d1	no babA2	absent	neither	iceA1 only	PZ2 only
Sat464	**h254**	CP002071	South America	hspAmerInd	unknown	cag-neg	s1-m1	i1-d1	babA2+	absent	neither	only iceA2	neither
Shi112	**h255**	CP003474	South America	hspAmerInd	gastritis	cag+	s1-m2	i2-d2	babA2+	long	917+918	only iceA2	neither
Shi169	**h256**	CP003473	South America	hspAmerInd	gastritis	cag+	s1-m1	i1-d1	babA2+	long	917+918	only iceA2	neither
Shi417	**h257**	CP003472	South America	hspAmerInd	gastritis	cag+	s1-m1	i1-d1	babA2+	long	917+918	iceA1 only	neither
Shi470	**h258**	NC_010698	South America	hspAmerInd	peptic ulcer	cag+	s1-m1	i1-d1	babA2+	long	917+918	only iceA2	neither
SJM180	**h259**	NC_014560	South America	hpEurope	gastritis	cag+	s1-m1	i1-d1	babA2+	absent	neither	iceA1 only	PZ1+PZ2
SNT49	**h260**	CP002983	(Other) India	hpAsia2	unknown	cag+	s1-m2	i1-d1	babA2+	long	917+918	only iceA2	PZ1 only
SouthAfrica20	**h261**	CP006691	Africa	hpAfrica2	unknown	cag-neg	s2-m2	i2-d2	babA2+	long	917+918	neither	PZ1 only
SouthAfrica50	h262	AVNI01	Africa	hpAfrica2	unknown	cag-neg	s2-m2	i2-d2	babA2+	long	917+918	iceA1 only	neither
SouthAfrica7	**h263**	CP002336	Africa	hpAfrica2	unknown	cag-neg	s2-?	i2-?	babA2+	long	917+918	iceA1 only	PZ1 only
UM007	h264	AONM01	East Asia	hspEAsia	unknown	cag+	s1-m2	i1-d1	no babA2	short	neither	iceA1 only	neither
UM018	h265	AONK01	East Asia	hpAsia2	unknown	cag+	s1-m1	i1-d1	babA2+	absent	only 918	iceA1 only	PZ1+PZ2
UM023	h266	AUSK01	East Asia	hspEAsia	peptic ulcer	cag+	s1-m1	i1-d1	babA2+	absent	neither	iceA1 only	PZ2 only
UM032	**h267**	CP005490	East Asia	hspEAsia	unknown	cag+	s1-m2	i1-d1	babA2+	absent	neither	iceA1 only	PZ1 only
UM034	h268	AONN01	East Asia	hspEAsia	unknown	cag+	s1-m2	i1-d1	babA2+	long	only 918	iceA1+A2	PZ2 only
UM037	h269	AUSI01	East Asia	hpEurope	gastric cancer	cag+	s1-?	i1-d1	babA2+	absent	only 918	only iceA2	PZ1+PZ2
UM038	h270	AUSL01	East Asia	hspEAsia	dyspepsia	cag+	s1-m2	i1-d1	babA2+	short	neither	iceA1 only	PZ2 only
UM045	h271	AONO01	East Asia	hpEurope	unknown	cag+	s1-m1	i1-d1	no babA2	absent	neither	iceA1+A2	PZ1 only
UM054	**h272**	AONL01	East Asia	hpAsia2	unknown	cag+	s2-m2	i2-d2	babA2+	absent	only 918	only iceA2	PZ1 only
UM065	h273	AUSM01	East Asia	hspEAsia	peptic ulcer	cag+	s1-m1	i1-d1	no babA2	absent	neither	iceA1 only	neither
UM066	h274	AUSJ01	East Asia	hspEAsia	peptic ulcer	cag+	s1-m2	i1-d1	babA2+	long	917+918	iceA1 only	PZ2 only
UM067	h275	AUSN01	East Asia	hpAsia2	peptic ulcer	cag+	s1-m2	i2-d2	babA2+	absent	neither	only iceA2	PZ2 only
UM077	h276	AUSQ01	East Asia	hspEAsia	peptic ulcer	cag+	s1-m1	i1-d1	babA2+	short	neither	iceA1 only	PZ2 only
UM084	h277	AUSO01	East Asia	hpAsia2	peptic ulcer	cag+	s1-m1	i1-d1	babA2+	absent	neither	iceA1 only	PZ2 only
UM085	h278	AUSP01	East Asia	hspEAsia	dyspepsia	cag+	s1-m2	i1-d1	babA2+	short	neither	iceA1 only	PZ2 only
UM111	h279	AUSR01	East Asia	hspEAsia	dyspepsia	cag+	s1-m2	i1-d1	babA2+	short	neither	iceA1 only	PZ1 only
UM114	h280	AUSS01	East Asia	hpAsia2	peptic ulcer	cag+	s1-m1	i1-d1	babA2+	absent	neither	only iceA2	PZ2 only
UM298	**h281**	CP006610	East Asia	hspEAsia	unknown	cag+	s1-m2	i1-d1	babA2+	absent	neither	iceA1 only	neither
UM299	**h282**	CP005491	East Asia	hspEAsia	unknown	cag+	s1-m2	i1-d1	babA2+	absent	neither	iceA1 only	neither
UMB_G1	h283	AOTV01	North America	hpEurope	unknown	cag-neg	s2-m2	i2-d2	no babA2	absent	neither	iceA1+A2	neither
v225	**h284**	CP001582	South America	hspAmerInd	gastritis	cag+	s1-m1	i1-d1	babA2+	absent	neither	iceA1 only	neither
wls-5-12	h285	AUPY01	East Asia	hspEAsia	gastritis	cag+	s1-m1	i1-d1	babA2+	absent	only 918	iceA1+A2	PZ1 only
wls-5-3	h286	AUPD01	East Asia	hspEAsia	gastritis	cag+	s1-m1	i1-d1	babA2+	short	neither	only iceA2	PZ2 only
X47-2AL	h287	AWNG01	North America	hpEurope	cat	cag-neg	s1-m2	i2-d2	no babA2	long	neither	iceA1 only	PZ1+PZ2
XZ274	**h288**	CP003419	East Asia	hspEAsia	gastric cancer	cag+	s1-m1	i1-d1	babA2+	long	917+918	iceA1 only	neither
SA144A	h289	CBNJ01	Africa	hpAfrica2	unknown	cag-neg	?-?	i2-?	no babA2	long	only 918	iceA1 only	PZ1+PZ2
SA144C	h290	CBOX01	Africa	hpAfrica2	unknown	cag-neg	?-?	i2-?	no babA2	long	only 918	iceA1 only	PZ1+PZ2
SA146A	h291	CBPZ01	Africa	hpAfrica1	unknown	cag+	s1-m1	i1-d1	babA2+	short	917+918	neither	PZ1+PZ2
SA146C	h292	CBPF01	Africa	hpAfrica1	unknown	cag+	s1-m1	i1-d1	babA2+	short	917+918	neither	PZ1+PZ2
SA155A	h293	CBNZ01	Africa	hpAfrica2	unknown	cag-neg	s2-?	i2-d1	no babA2	long	917+918	neither	PZ2 only
SA155C	h294	CBOH01	Africa	hpAfrica2	unknown	cag-neg	s2-?	i2-?	no babA2	long	917+918	neither	PZ2 only
SA156A	h295	CBNR01	Africa	hpAfrica1	unknown	cag+	s1-m1	i1-d1	babA2+	short	917+918	iceA1 only	PZ1+PZ2
SA157A	h296	CBPV01	Africa	hpAfrica1	unknown	cag+	s1-m1	i1-d1	babA2+	short	917+918	only iceA2	PZ1+PZ2
SA157C	h297	CBNF01	Africa	hpAfrica1	unknown	cag+	s1-m1	i1-d1	babA2+	short	917+918	only iceA2	PZ1 only
SA158A	**h298**	CBPJ01	Africa	hpAfrica1	unknown	cag+	s1-m1	i1-d1	no babA2	short	917+918	iceA1 only	PZ1 only
SA158C	h299	CBOL01	Africa	hpAfrica1	unknown	cag+	s1-m1	i1-d1	no babA2	short	917+918	iceA1 only	PZ1 only
SA160A	h300	CBPY01	Africa	hpAfrica2	unknown	cag-neg	s2-?	i2-?	no babA2	long	neither	neither	PZ2 only
SA160C	h301	CBQN01	Africa	hpAfrica2	unknown	cag-neg	s2-?	i2-?	no babA2	long	neither	neither	PZ2 only

SA161A	h302	CBPE01	Africa	hpAfrica1	unknown	cag-neg	s1-m1	i1-d1	babA2+	short	917+918	only iceA2	neither
SA161C	h303	CBNM01	Africa	hpAfrica1	unknown	cag+	s1-m1	i1-d1	babA2+	short	917+918	only iceA2	PZ1 only
SA162A	h304	CBOS01	Africa	hpAfrica1	unknown	cag+	s1-m1	i1-d1	babA2+	short	917+918	only iceA2	PZ1+PZ2
SA162C	h305	CBNQ01	Africa	hpAfrica1	unknown	cag+	s1-m1	i1-d1	babA2+	short	917+918	only iceA2	PZ1+PZ2
SA163A	h306	CBOG01	Africa	hpAfrica1	unknown	cag+	s1-m1	i1-d1	no babA2	short	917+918	iceA1 only	PZ1 only
SA163C	h307	CBOW01	Africa	hpAfrica1	unknown	cag+	s1-m1	i1-d1	no babA2	short	917+918	iceA1 only	PZ1 only
SA164A	h308	CBQK01	Africa	hpEurope	unknown	cag-neg	s2-m2	i2-d2	babA2+	long	917+918	iceA1+A2	PZ1+PZ2
SA164C	h309	CBQM01	Africa	hpEurope	unknown	cag-neg	s2-m2	i2-d2	babA2+	short	917+918	iceA1+A2	PZ2 only
SA165A	h310	CBQD01	Africa	hpEurope	unknown	cag-neg	s2-m2	i2-d2	babA2+	long	917+918	only iceA2	PZ1+PZ2
SA165C	h311	CBQJ01	Africa	hpEurope	unknown	cag-neg	s2-m2	i2-d2	babA2+	short	917+918	iceA1+A2	PZ2 only
SA166A	h312	CBPA01	Africa	hpAfrica2	unknown	cag-neg	?-?	i2-?	no babA2	short	917+918	iceA1 only	neither
SA168A	h313	CBPU01	Africa	hpAfrica1	unknown	cag+	s1-m2	i1-d1	babA2+	short	917+918	only iceA2	PZ1+PZ2
SA168C	h314	CBOC01	Africa	hpAfrica1	unknown	cag+	s1-m2	i1-d1	babA2+	short	917+918	only iceA2	PZ1+PZ2
SA169C	h315	CBOK01	Africa	hpAfrica2	unknown	cag-neg	s2-?	i2-?	no babA2	short	917+918	iceA1 only	PZ2 only
SA170A	h316	CBMZ01	Africa	hpAfrica1	unknown	cag+	s1-m1	i1-d1	babA2+	short	917+918	iceA1 only	PZ1 only
SA170C	h317	CBQL01	Africa	hpAfrica1	unknown	cag+	s1-m1	i1-d1	babA2+	short	917+918	iceA1 only	PZ1 only
SA171A	h318	CBOA01	Africa	hpEurope	unknown	cag-neg	?-?	i2-?	babA2+	long	917+918	iceA1+A2	PZ2 only
SA171C	h319	CBPC01	Africa	hpEurope	unknown	cag-neg	?-?	i2-?	babA2+	long	917+918	iceA1+A2	PZ2 only
SA172A	h320	CBOY01	Africa	hpEurope	unknown	cag-neg	?-?	i2-?	babA2+	long	917+918	iceA1+A2	PZ2 only
SA172C	h321	CBPG01	Africa	hpAfrica2	unknown	cag-neg	s2-?	i2-?	no babA2	absent	neither	iceA1 only	PZ1+PZ2
SA173A	h322	CBOU01	Africa	hpEurope	unknown	cag-neg	s2-m2	i2-d2	babA2+	absent	neither	only iceA2	neither
SA173C	h323	CBNG01	Africa	hpEurope	unknown	cag-neg	s2-m2	i2-d2	babA2+	absent	neither	only iceA2	PZ2 only
SA174A	h324	CBPS01	Africa	hpAfrica2	unknown	cag-neg	?-?	i2-?	no babA2	long	917+918	iceA1 only	PZ2 only
SA175A	h325	CBPO01	Africa	hpAfrica2	unknown	cag-neg	?-?	i2-?	babA2+	long	917+918	iceA1 only	PZ1 only
SA175C	h326	CBOI01	Africa	hpAfrica2	unknown	cag-neg	?-?	i2-?	babA2+	long	917+918	iceA1 only	PZ1 only
SA194A	h327	CBNK01	Africa	hpAfrica2	unknown	cag-neg	?-?	i2-?	babA2+	long	neither	iceA1 only	neither
SA194C	h328	CBPW01	Africa	hpAfrica2	unknown	cag-neg	?-?	i2-?	babA2+	long	neither	iceA1 only	neither
SA210A	**h329**	CBNW01	Africa	hpAfrica1	unknown	cag+	s1-m1	i1-d1	no babA2	short	917+918	iceA1 only	PZ1+PZ2
SA210C	h330	CBOQ01	Africa	hpAfrica1	unknown	cag+	s1-m1	i1-d1	no babA2	short	917+918	iceA1 only	PZ1+PZ2
SA213A	h331	CBOM01	Africa	hpEurope	unknown	cag-neg	?-m1	i2-?	no babA2	absent	only 918	iceA1+A2	PZ1 only
SA214A	**h332**	CBPK01	Africa	hpAfrica1	unknown	cag-neg	s1-m1	i1-d1	no babA2	short	917+918	iceA1+A2	neither
SA214C	h333	CBNS01	Africa	hpAfrica1	unknown	cag-neg	s1-m1	i1-d1	no babA2	short	917+918	only iceA2	PZ1 only
SA215C	h334	CBNO01	Africa	hpAfrica1	unknown	cag+	s1-m1	i1-d1	no babA2	short	917+918	only iceA2	PZ1 only
SA216C	h335	CBNC01	Africa	hpAfrica1	unknown	cag+	s1-m1	i1-d1	no babA2	short	only 918	iceA1+A2	PZ1 only
SA220A	h336	CBND01	Africa	hpAfrica1	unknown	cag+	s1-m2	i1-d1	no babA2	short	917+918	only iceA2	PZ1+PZ2
SA220C	h337	CBPX01	Africa	hpAfrica1	unknown	cag+	s1-m2	i1-d1	no babA2	short	917+918	only iceA2	PZ1+PZ2
SA221A	h338	CBQA01	Africa	hpEurope	unknown	cag-neg	s2-m2	i2-d2	babA2+	absent	only 918	iceA1+A2	PZ1 only
SA221C	h339	CBPT01	Africa	hpEurope	unknown	cag-neg	s2-m2	i2-d2	babA2+	absent	only 918	iceA1+A2	PZ1+PZ2
SA222A	h340	CBQE01	Africa	hpAsia2	unknown	cag+	s1-m1	i1-d1	babA2+	absent	neither	neither	PZ1 only
SA222C	**h341**	CBON01	Africa	hpAsia2	unknown	cag-neg	s1-m1	i1-d1	babA2+	absent	neither	iceA1 only	PZ1 only
SA224A	h342	CBOB01	Africa	hpAfrica1	unknown	cag+	s1-m1	i1-d1	babA2+	short	917+918	only iceA2	PZ1+PZ2
SA224C	h343	CBPP01	Africa	hpAfrica1	unknown	cag+	s1-m1	i1-d1	babA2+	short	917+918	only iceA2	PZ1+PZ2
SA226A	h344	CBNH01	Africa	hpAfrica1	unknown	cag+	s1-m1	i1-d1	babA2+	long	917+918	only iceA2	PZ1+PZ2
SA227A	h345	CBNX01	Africa	hpAfrica1	unknown	cag+	s1-m1	i1-d1	no babA2	short	917+918	only iceA2	PZ1 only
SA233A	h346	CBMX01	Africa	hpAfrica2	unknown	cag-neg	s2-?	i2-?	no babA2	long	only 918	iceA1 only	PZ1 only
SA233C	h347	CBPD01	Africa	hpAfrica2	unknown	cag-neg	?-?	i2-?	no babA2	long	only 918	iceA1 only	PZ1 only
SA251A	h348	CBOZ01	Africa	hpAfrica2	unknown	cag-neg	?-?	i2-?	no babA2	long	only 918	neither	PZ1+PZ2
SA251C	h349	CBQC01	Africa	hpAfrica2	unknown	cag-neg	?-?	i2-?	no babA2	long	only 918	iceA1 only	PZ1 only
SA252A	h350	CBNP01	Africa	hpAfrica1	unknown	cag+	s1-m1	i1-d1	babA2+	short	917+918	iceA1+A2	PZ1+PZ2
SA252C	h351	CBOV01	Africa	hpAfrica1	unknown	cag+	s1-m1	i1-d1	babA2+	short	917+918	only iceA2	PZ1+PZ2
SA253A	h352	CBOJ01	Africa	hpAfrica2	unknown	cag-neg	s2-?	i2-?	babA2+	long	neither	neither	PZ1+PZ2

SA253C	h353	CBOR01	Africa	hpAfrica2	unknown	cag-neg	s2-?	i2-?	no babA2	long	neither	neither	PZ1+PZ2
SA29A	h354	CBMV01	Africa	hpAfrica2	unknown	cag-neg	?-?	i2-?	babA2+	long	917+918	iceA1 only	neither
SA29C	h355	CBNI01	Africa	hpAfrica1	unknown	cag+	s1-m1	i1-d1	babA2+	short	917+918	only iceA2	PZ1+PZ2
SA300A	h356	CBQG01	Africa	hpAfrica1	unknown	cag+	s1-m1	i1-d1	no babA2	short	917+918	iceA1 only	PZ1 only
SA300C	h357	CBPL01	Africa	hpAfrica1	unknown	cag+	s1-m1	i1-d1	no babA2	short	917+918	iceA1 only	PZ1 only
SA301A	h358	CBPH01	Africa	hpAfrica1	unknown	cag+	s1-m1	i1-d1	babA2+	short	917+918	only iceA2	PZ1 only
SA301C	h359	CBNT01	Africa	hpAfrica1	unknown	cag+	s1-m1	i1-d1	babA2+	short	917+918	only iceA2	PZ1 only
SA302A	h360	CBOF01	Africa	hpEurope	unknown	cag-neg	?-?	i2-?	babA2+	short	only 918	iceA1+A2	PZ1 only
SA302C	h361	CBNA01	Africa	hpEurope	unknown	cag-neg	?-?	i2-?	babA2+	long	917+918	iceA1+A2	PZ1 only
SA303C	h362	CBMW01	Africa	hpAfrica2	unknown	cag-neg	s2-m2	i2-d2	no babA2	absent	only 918	iceA1 only	PZ1+PZ2
SA30A	h363	CBPQ01	Africa	hpAfrica1	unknown	cag+	s1-m1	i1-d1	no babA2	short	917+918	iceA1 only	PZ1+PZ2
SA30C	h364	CBQB01	Africa	hpAfrica1	unknown	cag+	s1-m1	i1-d1	no babA2	short	917+918	iceA1 only	PZ1+PZ2
SA31C	h365	CBPM01	Africa	hpAfrica1	unknown	cag+	s1-m1	i1-d1	no babA2	short	917+918	iceA1 only	PZ1 only
SA34A	h366	CBPI01	Africa	hpAfrica2	unknown	cag-neg	s2-m2	i2-d2	no babA2	long	917+918	neither	PZ1 only
SA34C	h367	CBNY01	Africa	hpAfrica2	unknown	cag-neg	s2-?	i2-?	no babA2	absent	neither	iceA1 only	PZ1 only
SA35A	h368	CBNE01	Africa	hpAfrica1	unknown	cag+	s1-m1	i1-d1	no babA2	short	917+918	iceA1 only	PZ1 only
SA36C	h369	CBQI01	Africa	hpAfrica2	unknown	cag-neg	?-?	i2-?	no babA2	short	917+918	iceA1 only	PZ1 only
SA37A	h370	CBOO01	Africa	hpEurope	unknown	cag-neg	s2-?	i2-?	no babA2	absent	only 918	iceA1 only	neither
SA37C	h371	CBOP01	Africa	hpEurope	unknown	cag-neg	s2-?	i2-?	no babA2	absent	only 918	iceA1 only	neither
SA40A	h372	CBNN01	Africa	hpAfrica2	unknown	cag-neg	s2-m2	i2-d2	no babA2	long	917+918	iceA1 only	PZ1+PZ2
SA40C	h373	CBPN01	Africa	hpAfrica2	unknown	cag-neg	s2-m2	i2-d2	no babA2	absent	neither	iceA1 only	PZ1 only
SA45A	h374	CBNB01	Africa	hpAfrica1	unknown	cag+	s1-m1	i1-d1	no babA2	short	917+918	iceA1 only	PZ1+PZ2
SA45C	h375	CBPR01	Africa	hpAfrica1	unknown	cag+	s1-m1	i1-d1	no babA2	short	917+918	iceA1 only	PZ1+PZ2
SA46C	h376	CBOD01	Africa	hpAfrica1	unknown	cag+	s1-m1	i1-d1	babA2+	short	917+918	iceA1 only	PZ1 only
SA47A	h377	CBOT01	Africa	hpAfrica2	unknown	cag-neg	s2-m2	i2-d2	no babA2	long	917+918	only iceA2	PZ1+PZ2
SA47C	h378	CBPB01	Africa	hpAfrica2	unknown	cag-neg	s2-m2	i2-d2	no babA2	long	917+918	only iceA2	PZ1+PZ2
Sahul64	h379	ALWV01	(Other) Australia	hpSahul	unknown	cag-neg	?-?	?-?	no babA2	long	only 918	iceA1+A2	PZ1 only

Table S3. Primers used for in silico PCR genotyping of *H. pylori* virulence markers.

Marker	Forward Primer (5' to 3')	Reverse Primer (5' to 3')	Size (nt)	Source
cagA	AATGCTAAATTAGACAACTTGAGCGA	AGAATAATCAACAAACATCACGCCAT	298	(7)
cagC	ATGAAATTTTTTACAAGAAT	TTAGCTAGCTCCTCCACCCT	328	(8)
cagE	GCGATTGTTATTGTGCTTGTAG	GAAGTGGTTAAAAATCAATGCCCC	329	(9)
cagG	GCCATGTTAACACCCCCTAG	TTAATGCGCTAGAATAGTGC	497	(10)
cagM	CAAATACAAAAAAGAAAAAGAGGC	ATTTTTCAACAAGTTAGAAAAAGCC	587	(10)
cagN	GTCGCTCTTTATTCTTTAGT	CTATTTTTTTCCATGAGCGA	890	(8)
cagT	TCTAAAAAGATTACGCTCATAGGCG	CTTTGGCTTGCATGTTCAAGTTGCC	490	(10)
vacAs1	ATGGAAATACAACAAACACAC	CTGCTTGAATGCGCCAAAC	259	(11)
vacAs2	ATGGAAATACAACAAACACAC	CTGCTTGAATGCGCCAAAC	286	(11)
vacAm1	CAATCTGTCCAATCAAGCGAG	GCGTCTAAATAATTCCAAGG	570	(12)
vacAm2	CAATCTGTCCAATCAAGCGAG	GCGTCTAAATAATTCCAAGG	645	(12)
vacAi1	GTTGGGATTGGGGGAATGCCG	TTAATTTAACGCTGTTTGAAG	426	(13)
vacAi2	GTTGGGATTGGGGGAATGCCG	GATCAACGCTCTGATTTGA	432	(13)
vacAd1	ACTAATATTGGCACACTGGATTTG	CTCGCTTGATTGGACAGATTG	367	(14)
vacAd2	ACTAATATTGGCACACTGGATTTG	CTCGCTTGATTGGACAGATTG	298	(14)
babA2	AATCCAAAAAGGAGAAAAAGTATGAAA	TGTTAGTGATTTCGGTGTAGGACA	832	(15)
dupAL	ATGTTTCTTGGTTTAGAGGG	TTATACATATTGAATATTCTCGC	2499	(16)
dupAS	GGTTTCTACTGACAGAGCGC	CGTATTTAGTCAGTAAGTTGGCG	468	(16)
dupA	TAAGCGTGATCAATATGGATT	GGAACGCCGCATTCTATTA	350	(7)
iceA1	GTGTTTTTAACCAAAGTATC	CTATAGCCASTYTCTTTGCA	247	(17)
iceA2	GTTGGGTATATCACAATTTAT	TTTCCCTATTTTCTAGTAGGT	229	(17)
jhp0917	TGGTTTCTACTGACAGAGCGC	AACACGCTGACAGGACAATCTCCC	307	(7)
jhp0918	AAGCTGAAGCGTTTGTAACG	CCTATATCGCTAACGCGCTCG	276	(7)
jhp940	GAAATGTCCTATACCAATGG	CCTAAGTAGTGCATCAAGG	591	(10)
jhp940	AGCACAAGATACATGGACCAACA	TCATCTGCGTTGTCATGGGA	473	(10)
jhp945	TGCAATAGGCAGTGGTATTGATAC	AGTAGTATTGCGTTTGCTTGATG	450	(10)
jhp945	ACTCCAGCCAGTATTGTAAA	TTCTTGCGAGTTAGGATTGG	174	(10)
jhp947	CAAGATAGAGCGTTTAGTCA	TGCATAGCTTTGCATTTGCA	348	(10)
jhp947	GATAATCCTACGCAGAACG	GCTAAAGTCATTTGGCTGTC	611	(10)
jhp949	CAAGATTCTTTAGTTGTGCA	CAGCATTTAGCAAGTTAGCAG	358	(10)
jhp949	ATAGGAGTGGGTGCTTACTT	AGCAACAACAAAGGCATGTA	788	(10)
jhp926	CAGGTTTGAACACCTTTTGA	CTTAAGCAAGAACCACCACA	550	(10)
jhp926	ACCTTTCAATACCGCTAGAAG	GATGAGCAAATCAATGGCATG	994	(10)

Table S4. Presence / absence analysis of genes belonging to the *H. pylori cag* pathogenicity island.

[a] Genome number labeled **blue** are included in Figure 2

[b] based on PCRs using primers listed in Table S3

[c] based on nomenclature of *H. pylori* strain 26695, red genes are essential for function, light blue are non-essential. Black blocks, gene and full length protein present; white, gene absent; yellow, gene present, no full length protein. See also Fig. 5

[d] classification: cag+, functional *cag* PAI; cag-neg (white), *cag* PAI absent, cag-neg (yellow): parts of *cag* PAI present, cag-neg (blue), essential *cag* genes inactivated

			cag PAI genes [c]																							
**Isolate name**	**number** [a]	***cag* by PCR** [b]	**1**	**3**	**4**	**β**	**α**	**Z**	**Y**	**X**	**W**	**V**	**U**	**T**	**M**	**N**	**L**	**I**	**H**	**G**	**F**	**E**	**D**	**C**	**A**	**Classification** [d]
2017	**h001**	cag+																								cag+
2018	**h002**	cag+																								cag+
26695	**h003**	cag+																								cag+
35A	**h004**	cag+																								cag+
51	**h005**	cag+																								cag+
52	**h006**	cag+																								cag+
83	**h007**	cag+																								cag+
8A3	h008	cag-neg																								cag-neg
908	**h009**	cag+																								cag+
98-10	h010	cag+																								cag+
A45	h011	cag-neg																								cag-neg
Aklavik117	**h012**	cag-neg																								cag-neg
Aklavik86	**h013**	cag-neg																								cag-neg
B128	h014	cag+																								cag+
B38	**h015**	cag-neg																								cag-neg
B45	h016	cag+																								cag-neg
B8	**h017**	cag+																								cag+
BCS100H1	h018	cag-neg																								cag-neg
BM012A	**h019**	cag+																								cag+
BM012S	**h020**	cag+																								cag+
CCHI_33	**h021**	cag+																								cag+
CG-IMSS-2012	h022	cag+																								cag+
CPY1124	h023	cag+																								cag+
CPY1313	h024	cag+																								cag+
CPY1662	h025	cag+																								cag+
CPY1962	h026	cag+																								cag+
CPY3281	h027	cag+																								cag+
CPY6081	h028	cag+																								cag-neg
CPY6261	h029	cag+																								cag+
CPY6271	h030	cag+																								cag+
CPY6311	h031	cag+																								cag+
Cuz20	**h032**	cag+																								cag+
E48	h033	cag+																								cag+

ELS37	**h034**	cag+	[redacted]	cag+
F16	**h035**	cag+	[redacted]	cag+
F30	**h036**	cag+	[redacted]	cag+
F32	**h037**	cag+	[redacted]	cag+
F57	**h038**	cag+	[redacted]	cag+
FD423	**h039**	cag+	[redacted]	cag+
FD430	h040	cag+	[redacted]	cag+
FD506	h041	cag+	[redacted]	cag+
FD535	h042	cag+	[redacted]	cag+
FD568	h043	cag+	[redacted]	cag+
FD577	h044	cag+	[redacted]	cag+
FD662	h045	cag+	[redacted]	cag+
FD703	h046	cag+	[redacted]	cag+
FD719	h047	cag+	[redacted]	cag+
G27	**h048**	cag+	[redacted]	cag+
GAM100Ai	h049	cag+	[redacted]	cag+
GAM101Biv	h050	cag-neg		cag-neg
GAM103Bi	**h051**	cag+	[redacted]	cag+
GAM105Ai	h052	cag+	[redacted]	cag+
GAM112Ai	h053	cag+	[redacted]	cag+
GAM114Ai	h054	cag+	[redacted]	cag+
GAM115Ai	h055	cag+	[redacted]	cag+
GAM117Ai	h056	cag+	[redacted]	cag+
GAM118Bi	h057	cag+	[redacted]	cag+
GAM119Bi	h058	cag+	[redacted]	cag+
GAM120Ai	h059	cag+	[redacted]	cag+
GAM121Aii	h060	cag+	[redacted]	cag+
GAM201Ai	h061	cag-neg		cag-neg
GAM210Bi	h062	cag+	[redacted]	cag+
GAM231Ai	h063	cag-neg		cag-neg
GAM239Bi	h064	cag-neg		cag-neg
GAM244Ai	h065	cag-neg		cag-neg
GAM245Ai	h066	cag+	[redacted]	cag+
GAM246Ai	h067	cag+	[redacted]	cag+
GAM249T	h068	cag+	[redacted]	cag+
GAM250AFi	h069	cag+	[redacted]	cag+
GAM250T	h070	cag+	[redacted]	cag+
GAM252Bi	h071	cag+	[redacted]	cag+
GAM252T	h072	cag+	[redacted]	cag+
GAM254Ai	h073	cag+	[redacted]	cag+
GAM260ASi	h074	cag+	[redacted]	cag+
GAM260Bi	h075	cag+	[redacted]	cag+

GAM260BSi	**h076**	cag-neg	cag-neg
GAM263BFi	h077	cag+	cag+
GAM264Ai	h078	cag-neg	cag-neg
GAM265BSii	h079	cag+	cag+
GAM268Bii	h080	cag+	cag+
GAM270ASi	h081	cag+	cag+
GAM42Ai	h082	cag+	cag+
GAM71Ai	h083	cag-neg	cag-neg
GAM80Ai	h084	cag+	cag+
GAM83Bi	h085	cag-neg	cag-neg
GAM83T	h086	cag-neg	cag-neg
GAM93Bi	h087	cag+	cag+
GAM96Ai	h088	cag-neg	cag-neg
Gambia9424	**h089**	cag+	cag+
GAMchJs106B	h090	cag-neg	cag-neg
GAMchJs114i	h091	cag-neg	cag-neg
GAMchJs117Ai	h092	cag-neg	cag-neg
GAMchJs124i	h093	cag-neg	cag-neg
GAMchJs136i	h094	cag+	cag+
GC26	h095	cag+	cag+
H13-1	h096	cag+	cag+
HLJHP193	h097	cag+	cag+
HLJHP253	h098	cag+	cag+
HLJHP256	h099	cag+	cag+
HLJHP271	h100	cag+	cag+
Hp_A-11	h101	cag+	cag+
Hp_A-14	h102	cag-neg	cag-neg
Hp_A-16	**h103**	cag+	cag+
Hp_A-17	h104	cag-neg	cag-neg
Hp_A-20	h105	cag+	cag+
Hp_A-26	h106	cag-neg	cag-neg
Hp_A-27	h107	cag+	cag+
Hp_A-4	h108	cag+	cag+
Hp_A-5	h109	cag+	cag+
Hp_A-6	h110	cag+	cag+
Hp_A-8	h111	cag+	cag+
Hp_A-9	h112	cag+	cag+
Hp_H-1	h113	cag+	cag+
Hp_H-10	h114	cag+	cag-neg
Hp_H-11	h115	cag+	cag+
Hp_H-16	h116	cag+	cag+
Hp_H-18	h117	cag+	cag+

Hp_H-19	h118	cag+	cag+
Hp_H-21	h119	cag-neg	cag-neg
Hp_H-23	h120	cag+	cag+
Hp_H-24	h121	cag+	cag-neg
Hp_H-24b	h122	cag+	cag+
Hp_H-24c	h123	cag+	cag-neg
Hp_H-27	h124	cag-neg	cag-neg
Hp_H-28	h125	cag+	cag+
Hp_H-29	h126	cag+	cag+
Hp_H-3	h127	cag+	cag+
Hp_H-30	h128	cag+	cag-neg
Hp_H-34	**h129**	cag-neg	cag-neg
Hp_H-36	h130	cag+	cag+
Hp_H-4	h131	cag+	cag+
Hp_H-41	h132	cag+	cag+
Hp_H-42	h133	cag+	cag-neg
Hp_H-43	h134	cag-neg	cag-neg
Hp_H-44	h135	cag+	cag+
Hp_H-45	h136	cag+	cag+
Hp_H-5b	h137	cag+	cag+
Hp_H-6	h138	cag+	cag+
Hp_H-9	h139	cag+	cag+
Hp_M1	h140	cag+	cag+
Hp_M2	h141	cag+	cag+
Hp_M3	h142	cag+	cag+
Hp_M4	h143	cag+	cag+
Hp_M5	h144	cag+	cag+
Hp_M6	h145	cag+	cag+
Hp_M9	**h146**	cag+	cag+
Hp_P-1	h147	cag+	cag+
Hp_P-11	h148	cag+	cag+
Hp_P-11b	h149	cag+	cag+
Hp_P-13	h150	cag+	cag+
Hp_P-13b	h151	cag+	cag+
Hp_P-15	h152	cag-neg	cag-neg
Hp_P-15b	h153	cag-neg	cag-neg
Hp_P-16	h154	cag-neg	cag-neg
Hp_P-1b	h155	cag+	cag+
Hp_P-2	h156	cag+	cag+
Hp_P-23	h157	cag-neg	cag-neg
Hp_P-25	h158	cag+	cag-neg
Hp_P-25c	h159	cag+	cag-neg

Hp_P-25d	h160	cag+	cag-neg
Hp_P-26	h161	cag+	cag+
Hp_P-28b	h162	cag-neg	cag-neg
Hp_P-2b	h163	cag+	cag+
Hp_P-3	h164	cag+	cag+
Hp_P-30	h165	cag-neg	cag-neg
Hp_P-3b	h166	cag+	cag+
Hp_P-4	h167	cag+	cag+
Hp_P-41	h168	cag+	cag+
Hp_P-4c	h169	cag+	cag+
Hp_P-4d	h170	cag+	cag+
Hp_P-62	h171	cag+	cag+
Hp_P-74	h172	cag-neg	cag-neg
Hp_P-8	h173	cag-neg	cag-neg
Hp_P-8b	h174	cag-neg	cag-neg
HP116Bi	**h175**	cag+	cag+
HP250AFii	h176	cag+	cag+
HP250AFiii	h177	cag+	cag+
HP250AFiV	h178	cag+	cag+
HP250ASi	h179	cag+	cag+
HP250ASii	h180	cag+	cag+
HP250BFi	h181	cag+	cag+
HP250BFii	h182	cag+	cag+
HP250BFiii	h183	cag+	cag+
HP250BFiV	h184	cag+	cag+
HP250BSi	h185	cag+	cag+
HP260AFi	h186	cag+	cag+
HP260AFii	h187	cag+	cag+
HP260ASii	h188	cag+	cag+
HP260BFii	h189	cag-neg	cag-neg
HP260Bi	h190	cag+	cag+
HP87hu	h191	cag+	cag+
HP87P7	h192	cag+	cag+
HP87P7tlpDRI	h193	cag+	cag+
HP87tlpD	h194	cag+	cag+
HPAG1	**h195**	cag+	cag-neg
HPARG63	h196	cag+	cag+
HPARG8G	h197	cag+	cag+
HUP-B14	**h200**	cag+	cag+
India7	**h201**	cag+	cag+
J99	**h202**	cag+	cag+
Lithuania75	**h203**	cag+	cag+

MALT	h204	cag-neg	cag-neg
N6	h205	cag+	cag+
NAB47	**h206**	cag+	cag-neg
NAD1	h207	cag+	cag-neg
NAK7	h208	cag+	cag+
NCTC_11637	h209	cag+	cag+
NQ1671	h210	cag+	cag+
NQ1701	h211	cag+	cag+
NQ1707	h212	cag+	cag+
NQ1712	h213	cag-neg	cag-neg
NQ315	h214	cag+	cag+
NQ352	h215	cag+	cag+
NQ367	h216	cag+	cag+
NQ392	h217	cag+	cag+
NQ4044	h218	cag+	cag+
NQ4053	h219	cag-neg	cag-neg
NQ4060	h220	cag+	cag+
NQ4076	h221	cag+	cag+
NQ4099	h222	cag+	cag+
NQ4110	h223	cag-neg	cag-neg
NQ4161	h224	cag-neg	cag-neg
NQ4191	h225	cag+	cag+
NQ4200	h226	cag+	cag+
NQ4216	h227	cag+	cag+
NQ4228	h228	cag+	cag+
OK113	**h229**	cag+	cag+
OK310	**h230**	cag+	cag+
P12	**h231**	cag+	cag+
P79	h232	cag+	cag+
PeCan18	**h233**	cag+	cag+
PeCan4	**h234**	cag+	cag+
Puno120	**h235**	cag+	cag+
Puno135	**h236**	cag+	cag+
PZ5004	h237	cag+	cag+
PZ5024	h238	cag+	cag+
PZ5026	h239	cag+	cag+
PZ5056	h240	cag+	cag+
PZ5080	h241	cag+	cag+
PZ5086	h242	cag+	cag+
R018c	h243	cag+	cag+
R030b	**h244**	cag+	cag+
R036d	h245	cag+	cag+

R037c	h246	cag-neg	cag-neg
R038b	h247	cag-neg	cag-neg
R046Wa	h248	cag-neg	cag-neg
R055a	h249	cag+	cag+
R056a	h250	cag+	cag+
R32b	h251	cag+	cag+
Rif1	h252	cag+	cag+
Rif2	h253	cag+	cag+
Sat464	**h254**	cag+	cag-neg
Shi112	**h255**	cag+	cag+
Shi169	**h256**	cag+	cag+
Shi417	**h257**	cag-neg	cag+
Shi470	**h258**	cag-neg	cag+
SJM180	**h259**	cag+	cag+
SNT49	**h260**	cag+	cag+
SouthAfrica20	**h261**	cag-neg	cag-neg
SouthAfrica50	h262	cag-neg	cag-neg
SouthAfrica7	**h263**	cag-neg	cag-neg
UM007	h264	cag+	cag+
UM018	h265	cag+	cag+
UM023	h266	cag+	cag+
UM032	**h267**	cag+	cag+
UM034	h268	cag+	cag+
UM037	h269	cag+	cag+
UM038	h270	cag+	cag+
UM045	h271	cag+	cag+
UM054	**h272**	cag+	cag+
UM065	h273	cag+	cag+
UM066	h274	cag+	cag+
UM067	h275	cag+	cag+
UM077	h276	cag+	cag+
UM084	h277	cag+	cag+
UM085	h278	cag+	cag+
UM111	h279	cag+	cag+
UM114	h280	cag+	cag+
UM298	**h281**	cag+	cag+
UM299	**h282**	cag+	cag+
UMB_G1	h283	cag-neg	cag-neg
v225	**h284**	cag+	cag-neg
wls-5-12	h285	cag+	cag+
wls-5-3	h286	cag+	cag+
X47-2AL	h287	cag-neg	cag-neg

XZ274	**h288**	cag+	cag-neg
SA144A	h289	cag-neg	cag-neg
SA144C	h290	cag-neg	cag-neg
SA146A	h291	cag+	cag-neg
SA146C	h292	cag+	cag+
SA155A	h293	cag-neg	cag-neg
SA155C	h294	cag-neg	cag-neg
SA156A	h295	cag+	cag+
SA157A	h296	cag+	cag+
SA157C	h297	cag+	cag+
SA158A	**h298**	cag+	cag+
SA158C	h299	cag+	cag+
SA160A	h300	cag-neg	cag-neg
SA160C	h301	cag-neg	cag-neg
SA161A	h302	cag-neg	cag-neg
SA161C	h303	cag+	cag+
SA162A	h304	cag+	cag+
SA162C	h305	cag+	cag+
SA163A	h306	cag+	cag+
SA163C	h307	cag+	cag+
SA164A	h308	cag-neg	cag-neg
SA164C	h309	cag-neg	cag-neg
SA165A	h310	cag-neg	cag-neg
SA165C	h311	cag-neg	cag-neg
SA166A	h312	cag-neg	cag-neg
SA168A	h313	cag+	cag+
SA168C	h314	cag+	cag+
SA169C	h315	cag-neg	cag-neg
SA170A	h316	cag+	cag+
SA170C	h317	cag+	cag+
SA171A	h318	cag-neg	cag-neg
SA171C	h319	cag-neg	cag-neg
SA172A	h320	cag-neg	cag-neg
SA172C	h321	cag-neg	cag-neg
SA173A	h322	cag-neg	cag-neg
SA173C	h323	cag-neg	cag-neg
SA174A	h324	cag-neg	cag-neg
SA175A	h325	cag-neg	cag-neg
SA175C	h326	cag-neg	cag-neg
SA194A	h327	cag-neg	cag-neg
SA194C	h328	cag-neg	cag-neg
SA210A	**h329**	cag+	cag+

SA210C	h330	cag+	cag+
SA213A	h331	cag-neg	cag-neg
SA214A	**h332**	cag-neg	cag-neg
SA214C	h333	cag-neg	cag-neg
SA215C	h334	cag+	cag+
SA216C	h335	cag+	cag-neg
SA220A	h336	cag+	cag+
SA220C	h337	cag+	cag+
SA221A	h338	cag-neg	cag-neg
SA221C	h339	cag-neg	cag-neg
SA222A	h340	cag+	cag+
SA222C	**h341**	cag-neg	cag-neg
SA224A	h342	cag+	cag+
SA224C	h343	cag+	cag+
SA226A	h344	cag+	cag+
SA227A	h345	cag+	cag+
SA233A	h346	cag-neg	cag-neg
SA233C	h347	cag-neg	cag-neg
SA251A	h348	cag-neg	cag-neg
SA251C	h349	cag-neg	cag-neg
SA252A	h350	cag+	cag+
SA252C	h351	cag+	cag+
SA253A	h352	cag-neg	cag-neg
SA253C	h353	cag-neg	cag-neg
SA29A	h354	cag-neg	cag-neg
SA29C	h355	cag+	cag+
SA300A	h356	cag+	cag+
SA300C	h357	cag+	cag+
SA301A	h358	cag+	cag+
SA301C	h359	cag+	cag+
SA302A	h360	cag-neg	cag-neg
SA302C	h361	cag-neg	cag-neg
SA303C	h362	cag-neg	cag-neg
SA30A	h363	cag+	cag+
SA30C	h364	cag+	cag+
SA31C	h365	cag+	cag+
SA34A	h366	cag-neg	cag-neg
SA34C	h367	cag-neg	cag-neg
SA35A	h368	cag+	cag+
SA36C	h369	cag-neg	cag-neg
SA37A	h370	cag-neg	cag-neg
SA37C	h371	cag-neg	cag-neg

SA40A	h372	cag-neg	cag-neg
SA40C	h373	cag-neg	cag-neg
SA45A	h374	cag+	cag+
SA45C	h375	cag+	cag+
SA46C	h376	cag+	cag+
SA47A	h377	cag-neg	cag-neg
SA47C	h378	cag-neg	cag-neg
Sahul64	h379	cag-neg	cag-neg

Table S5. Prediction of antibiotic resistance encoded in *H. pylori* genomes

[a] Genome number labeled **blue** are included in Figure 2

[b] based on continent/country of isolation

[c] resistance to clarithromycin is based on the presence of either A2142, A2143 or T2182 mutations in the 23S rRNA gene. WT = wildtype sequence, T2182C is annotated as "low level?"

[d] resistance to metronidazole is based on inactivation/truncation of the *rdxA* (*hp0954*) and *frxA* (*hp0642*) genes. WT = wildtype (*rdxA*[+] *frxA*[+])

[e] resistance to fluoroquinolone is based on the presence of either N87 or D91 mutations in the *gyrA* gene. WT = wildtype sequence. Only N87K, N87I, D91N, D91G and D91Y are scored as resistant

[f] resistance to tetracyline is based on the presence of AGA926-928 mutations in the 16S rRNA gene. WT = wildtype sequence. AGC, TGC and TGA are scored as low level resistance, AGT and GGA as susceptible

				Clarithromycin resistance (23S rRNA gene)[c]				Metronidazole resistance (*rdxA*/*frxA*)[d]			Fluoroquinolone resistance (*gyrA*)[e]			Tetracycline resistance (16S rRNA gene)[f]	
**Isolate**	**number[a]**	**geography[b]**	**MLST**	**clarithromycin**	**A2142G/C**	**A2143G**	**T2182C**	**metronidazole**	**rdxA**	**frxA**	**fluoroquinolone**	**N87**	**D91**	**tetracycline**	**AGA926-928**
2017	**h001**	Africa	hpAfrica1	susceptible	WT	WT	WT	resistant	rdxA-	WT	susceptible	N87T	WT	susceptible	WT
2018	**h002**	Africa	hpAfrica1	susceptible	WT	WT	WT	resistant	rdxA-	WT	susceptible	N87T	WT	susceptible	WT
26695	**h003**	Europe	hpEurope	susceptible	WT	WT	WT	susceptible	WT	WT	susceptible	WT	WT	susceptible	WT
35A	**h004**	North America	hspEAsia	susceptible	WT	WT	WT	susceptible	WT	WT	susceptible	WT	WT	susceptible	WT
51	**h005**	East Asia	hspEAsia	resistant	WT	A2143G	T2182C	susceptible	WT	WT	susceptible	WT	WT	susceptible	WT
52	**h006**	East Asia	hspEAsia	low level?	WT	WT	T2182C	susceptible	WT	WT	susceptible	WT	WT	susceptible	WT
83	**h007**	North America	hspEAsia	low level?	WT	WT	T2182C	susceptible	WT	WT	susceptible	WT	WT	susceptible	WT
8A3	h008	North America	hspEAsia	low level?	WT	WT	T2182C	susceptible	WT	WT	susceptible	WT	WT	susceptible	WT
908	**h009**	Africa	hpAfrica1	susceptible	WT	WT	WT	resistant	rdxA-	frxA-	susceptible	N87T	WT	susceptible	WT
98-10	h010	East Asia	hspEAsia	low level?	WT	WT	T2182C	susceptible	WT	WT	susceptible	WT	WT	susceptible	WT
A45	h011	Europe	hpEurope	susceptible	WT	WT	WT	resistant	WT	frxA-	susceptible	WT	WT	susceptible	WT
Aklavik117	**h012**	North America	hspAmerInd	low level?	WT	WT	T2182C	susceptible	WT	WT	susceptible	WT	WT	susceptible	WT
Aklavik86	**h013**	North America	hspAmerInd	low level?	WT	WT	T2182C	unknown	WT	n/a	susceptible	WT	WT	susceptible	WT
B128	h014	Europe	hpEurope	susceptible	WT	WT	WT	susceptible	WT	WT	susceptible	WT	WT	susceptible	WT
B38	**h015**	Europe	hpEurope	susceptible	WT	WT	WT	susceptible	WT	WT	susceptible	WT	WT	susceptible	WT
B45	h016	Europe	hpEurope	susceptible	WT	WT	WT	susceptible	WT	WT	susceptible	WT	WT	susceptible	WT
B8	**h017**	Europe	hpEurope	susceptible	WT	WT	WT	susceptible	WT	WT	susceptible	WT	WT	susceptible	WT
BCS100H1	h018	North America	hspEAsia	low level?	WT	WT	T2182C	susceptible	WT	WT	susceptible	WT	WT	susceptible	WT
BM012A	**h019**	other (Australia)	hpEurope	susceptible	WT	WT	WT	susceptible	WT	WT	susceptible	WT	WT	susceptible	WT
BM012S	**h020**	other (Australia)	hpEurope	susceptible	WT	WT	WT	susceptible	WT	WT	susceptible	WT	WT	susceptible	WT
CCHI_33	**h021**	North America	hpAfrica1	resistant	A2142G	WT	WT	resistant	WT	frxA-	susceptible	N87T	WT	unknown	N/A
CG-IMSS-2012	h022	South America	hpEurope	susceptible	WT	WT	WT	resistant	WT	frxA-	susceptible	WT	WT	susceptible	GGA
CPY1124	h023	East Asia	hspEAsia	unknown	unknown	unknown	unknown	susceptible	WT	WT	susceptible	WT	WT	susceptible	WT
CPY1313	h024	East Asia	hspEAsia	low level?	WT	WT	T2182C	resistant	WT	frxA-	susceptible	WT	WT	susceptible	WT
CPY1662	h025	East Asia	hspEAsia	low level?	WT	WT	T2182C	susceptible	WT	WT	susceptible	WT	WT	susceptible	WT
CPY1962	h026	East Asia	hspEAsia	unknown	unknown	unknown	unknown	resistant	WT	frxA-	susceptible	WT	WT	susceptible	WT
CPY3281	h027	East Asia	hspEAsia	low level?	WT	WT	T2182C	susceptible	WT	WT	susceptible	WT	WT	susceptible	WT
CPY6081	h028	East Asia	hspEAsia	low level?	WT	WT	T2182C	susceptible	WT	WT	susceptible	WT	WT	susceptible	WT
CPY6261	h029	East Asia	hspEAsia	low level?	WT	WT	T2182C	susceptible	WT	WT	susceptible	WT	WT	susceptible	WT
CPY6271	h030	East Asia	hspEAsia	low level?	WT	WT	T2182C	susceptible	WT	WT	susceptible	WT	WT	susceptible	WT
CPY6311	h031	East Asia	hspEAsia	unknown	unknown	unknown	unknown	resistant	WT	frxA-	susceptible	WT	WT	susceptible	WT
Cuz20	**h032**	South America	hspAmerInd	low level?	WT	WT	T2182C	susceptible	WT	WT	susceptible	WT	WT	susceptible	WT
E48	h033	other (Russia)	hspEAsia	low level?	WT	WT	T2182C	susceptible	WT	WT	susceptible	WT	WT	susceptible	WT
ELS37	**h034**	South America	hpEurope	susceptible	WT	WT	WT	resistant	WT	frxA-	susceptible	WT	WT	susceptible	WT
F16	**h035**	East Asia	hspEAsia	low level?	WT	WT	T2182C	resistant	WT	frxA-	susceptible	WT	WT	susceptible	WT
F30	**h036**	East Asia	hspEAsia	low level?	WT	WT	T2182C	resistant	WT	frxA-	susceptible	WT	WT	susceptible	WT
F32	**h037**	East Asia	hspEAsia	low level?	WT	WT	T2182C	susceptible	WT	WT	susceptible	WT	WT	susceptible	WT
F57	**h038**	East Asia	hspEAsia	low level?	WT	WT	T2182C	susceptible	WT	WT	susceptible	WT	WT	susceptible	WT
FD423	**h039**	East Asia	hpAsia2	low level?	WT	WT	T2182C	resistant	n/a	frxA-	susceptible	WT	WT	susceptible	WT
FD430	h040	East Asia	hpAsia2	low level?	WT	WT	T2182C	unknown	WT	n/a	susceptible	WT	WT	susceptible	WT
FD506	h041	East Asia	hspEAsia	low level?	WT	WT	T2182C	unknown	WT	n/a	susceptible	WT	WT	susceptible	WT
FD535	h042	East Asia	hpAsia2	low level?	WT	WT	T2182C	resistant	WT	frxA-	susceptible	WT	WT	susceptible	WT
FD568	h043	East Asia	hspEAsia	low level?	WT	WT	T2182C	resistant	WT	frxA-	susceptible	WT	WT	susceptible	WT
FD577	h044	East Asia	hspEAsia	low level?	WT	WT	T2182C	susceptible	WT	WT	susceptible	WT	WT	susceptible	WT
FD662	h045	East Asia	hpAsia2	low level?	WT	WT	T2182C	susceptible	WT	WT	susceptible	WT	WT	susceptible	WT
FD703	h046	East Asia	hpAsia2	low level?	WT	WT	T2182C	susceptible	WT	WT	susceptible	WT	WT	susceptible	WT

FD719	h047	East Asia	hpAsia2	low level?	WT	WT	T2182C	unknown	WT	n/a	susceptible	WT	WT	susceptible	WT
G27	**h048**	Europe	hpEurope	susceptible	WT	WT	WT	susceptible	WT	WT	susceptible	WT	WT	susceptible	WT
GAM100Ai	h049	Africa	hpAfrica1	susceptible	WT	WT	WT	resistant	rdxA-	frxA-	susceptible	WT	WT	susceptible	WT
GAM101Biv	h050	Africa	hpAfrica1	susceptible	WT	WT	WT	resistant	rdxA-	WT	susceptible	WT	WT	susceptible	WT
GAM103Bi	**h051**	Africa	hpAfrica1	susceptible	WT	WT	WT	resistant	WT	frxA-	susceptible	N87T	WT	susceptible	GGA
GAM105Ai	h052	Africa	hpAfrica1	susceptible	WT	WT	WT	susceptible	WT	WT	susceptible	WT	WT	susceptible	WT
GAM112Ai	h053	Africa	hpAfrica1	susceptible	WT	WT	WT	resistant	rdxA-	frxA-	susceptible	WT	WT	susceptible	WT
GAM114Ai	h054	Africa	hpAfrica1	susceptible	WT	WT	WT	resistant	WT	frxA-	susceptible	N87T	WT	susceptible	WT
GAM115Ai	h055	Africa	hpAfrica1	susceptible	WT	WT	WT	resistant	rdxA-	frxA-	susceptible	WT	WT	susceptible	WT
GAM117Ai	h056	Africa	hpAfrica1	susceptible	WT	WT	WT	resistant	WT	frxA-	resistant	N87T	D91N	susceptible	WT
GAM118Bi	h057	Africa	hpAfrica1	susceptible	WT	WT	WT	resistant	rdxA-	frxA-	susceptible	WT	WT	susceptible	WT
GAM119Bi	h058	Africa	hpAfrica1	susceptible	WT	WT	WT	susceptible	WT	WT	resistant	N87I	WT	susceptible	WT
GAM120Ai	h059	Africa	hpAfrica1	susceptible	WT	WT	WT	resistant	WT	frxA-	susceptible	N87T	WT	susceptible	WT
GAM121Aii	h060	Africa	hpAfrica1	susceptible	WT	WT	WT	resistant	WT	frxA-	susceptible	N87T	WT	susceptible	WT
GAM201Ai	h061	Africa	hpAfrica1	susceptible	WT	WT	WT	resistant	WT	frxA-	susceptible	WT	WT	susceptible	WT
GAM210Bi	h062	Africa	hpAfrica1	susceptible	WT	WT	WT	resistant	rdxA-	frxA-	susceptible	N87T	WT	susceptible	WT
GAM231Ai	h063	Africa	hpAfrica1	susceptible	WT	WT	WT	susceptible	WT	WT	susceptible	N87T	WT	susceptible	WT
GAM239Bi	h064	Africa	hpAfrica1	susceptible	WT	WT	WT	susceptible	WT	WT	susceptible	WT	WT	susceptible	WT
GAM244Ai	h065	Africa	hpAfrica1	susceptible	WT	WT	WT	resistant	rdxA-	frxA-	susceptible	N87T	WT	susceptible	WT
GAM245Ai	h066	Africa	hpAfrica1	susceptible	WT	WT	WT	resistant	WT	frxA-	susceptible	N87T	WT	susceptible	WT
GAM246Ai	h067	Africa	hpAfrica1	susceptible	WT	WT	WT	resistant	rdxA-	frxA-	susceptible	N87T	WT	susceptible	WT
GAM249T	h068	Africa	hpAfrica1	susceptible	WT	WT	WT	resistant	rdxA-	frxA-	resistant	N87T	D91N	susceptible	WT
GAM250AFi	h069	Africa	hpAfrica1	susceptible	WT	WT	WT	resistant	WT	frxA-	susceptible	N87T	WT	low level	AGC
GAM250T	h070	Africa	hpAfrica1	susceptible	WT	WT	WT	resistant	WT	frxA-	susceptible	N87T	WT	low level	AGC
GAM252Bi	h071	Africa	hpAfrica1	susceptible	WT	WT	WT	resistant	WT	frxA-	susceptible	N87T	WT	low level	AGC
GAM252T	h072	Africa	hpAfrica1	susceptible	WT	WT	WT	resistant	WT	frxA-	susceptible	N87T	WT	low level	AGC
GAM254Ai	h073	Africa	hpAfrica1	susceptible	WT	WT	WT	resistant	rdxA-	frxA-	susceptible	N87T	WT	susceptible	WT
GAM260ASi	h074	Africa	hpAfrica1	susceptible	WT	WT	WT	resistant	WT	frxA-	susceptible	N87T	WT	susceptible	WT
GAM260Bi	h075	Africa	hpAfrica1	susceptible	WT	WT	WT	resistant	WT	frxA-	susceptible	N87T	WT	susceptible	WT
GAM260BSi	**h076**	Africa	hpAfrica1	susceptible	WT	WT	WT	resistant	rdxA-	WT	susceptible	WT	WT	susceptible	WT
GAM263BFi	h077	Africa	hpAfrica1	susceptible	WT	WT	WT	resistant	rdxA-	frxA-	susceptible	WT	WT	susceptible	WT
GAM264Ai	h078	Africa	hpAfrica1	susceptible	WT	WT	WT	resistant	WT	frxA-	susceptible	N87T	WT	susceptible	WT
GAM265BSii	h079	Africa	hpAfrica1	susceptible	WT	WT	WT	resistant	WT	frxA-	susceptible	N87T	WT	susceptible	WT
GAM268Bii	h080	Africa	hpAfrica1	susceptible	WT	WT	WT	resistant	WT	frxA-	susceptible	N87T	WT	susceptible	WT
GAM270ASi	h081	Africa	hpAfrica1	susceptible	WT	WT	WT	resistant	rdxA-	frxA-	susceptible	N87T	WT	susceptible	WT
GAM42Ai	h082	Africa	hpAfrica1	susceptible	WT	WT	WT	susceptible	WT	WT	susceptible	WT	WT	susceptible	WT
GAM71Ai	h083	Africa	hpAfrica1	susceptible	WT	WT	WT	resistant	rdxA-	frxA-	susceptible	WT	WT	susceptible	WT
GAM80Ai	h084	Africa	hpAfrica1	susceptible	WT	WT	WT	susceptible	WT	WT	susceptible	WT	WT	susceptible	WT
GAM83Bi	h085	Africa	hpAfrica1	susceptible	WT	WT	WT	susceptible	WT	WT	resistant	N87K	WT	low level	TGC
GAM83T	h086	Africa	hpAfrica1	susceptible	WT	WT	WT	resistant	WT	frxA-	resistant	WT	D91G	low level	TGC
GAM93Bi	h087	Africa	hpAfrica1	susceptible	WT	WT	WT	resistant	rdxA-	frxA-	resistant	N87I	WT	susceptible	GGA
GAM96Ai	h088	Africa	hpAfrica1	susceptible	WT	WT	WT	resistant	rdxA-	frxA-	susceptible	N87T	WT	susceptible	WT
Gambia9424	**h089**	Africa	hpAfrica1	susceptible	WT	WT	WT	resistant	WT	frxA-	susceptible	WT	WT	susceptible	WT
GAMchJs106B	h090	Africa	hpAfrica1	susceptible	WT	WT	WT	resistant	WT	frxA-	susceptible	N87T	WT	susceptible	WT
GAMchJs114i	h091	Africa	hpAfrica1	susceptible	WT	WT	WT	resistant	rdxA-	frxA-	susceptible	N87T	WT	susceptible	WT
GAMchJs117Ai	h092	Africa	hpAfrica1	susceptible	WT	WT	WT	susceptible	WT	WT	susceptible	WT	WT	susceptible	WT
GAMchJs124i	h093	Africa	hpAfrica1	susceptible	WT	WT	WT	susceptible	WT	WT	susceptible	WT	WT	susceptible	WT
GAMchJs136i	h094	Africa	hpAfrica1	susceptible	WT	WT	WT	resistant	WT	frxA-	susceptible	N87T	WT	susceptible	WT
GC26	h095	East Asia	hspEAsia	low level?	WT	WT	T2182C	resistant	WT	frxA-	susceptible	WT	WT	susceptible	WT
H13-1	h096	other (Russia)	hspEAsia	low level?	WT	WT	T2182C	susceptible	WT	WT	susceptible	WT	WT	susceptible	WT
HLJHP193	h097	East Asia	hspEAsia	resistant	WT	A2143G	T2182C	resistant	WT	frxA-	susceptible	WT	WT	susceptible	WT
HLJHP253	h098	East Asia	hspEAsia	low level?	WT	WT	T2182C	susceptible	WT	WT	resistant	WT	D91G	susceptible	WT
HLJHP256	h099	East Asia	hspEAsia	low level?	WT	WT	T2182C	resistant	WT	frxA-	resistant	WT	D91G	susceptible	WT
HLJHP271	h100	East Asia	hspEAsia	low level?	WT	WT	T2182C	resistant	rdxA-	WT	susceptible	WT	WT	unknown	N/A
Hp_A-11	h101	North America	hpEurope	low level?	WT	WT	T2182C	susceptible	WT	WT	susceptible	WT	WT	susceptible	WT
Hp_A-14	h102	North America	hpEurope	susceptible	WT	WT	WT	susceptible	WT	WT	susceptible	WT	WT	unknown	N/A
Hp_A-16	**h103**	North America	hpAfrica1	susceptible	WT	WT	WT	susceptible	WT	WT	susceptible	N87T	WT	susceptible	WT

Hp_A-17	h104	North America	hpAfrica1	susceptible	WT	WT	WT	susceptible	WT	WT	susceptible	WT	WT	susceptible	WT
Hp_A-20	h105	North America	hpAfrica1	susceptible	WT	WT	WT	susceptible	WT	WT	susceptible	N87T	WT	susceptible	WT
Hp_A-26	h106	North America	hpEurope	susceptible	WT	WT	WT	resistant	WT	frxA-	susceptible	WT	WT	susceptible	WT
Hp_A-27	h107	North America	hpEurope	susceptible	WT	WT	WT	susceptible	WT	WT	susceptible	WT	WT	susceptible	WT
Hp_A-4	h108	North America	hpAfrica1	susceptible	WT	WT	WT	susceptible	WT	WT	susceptible	N87T	WT	unknown	N/A
Hp_A-5	h109	North America	hpAfrica1	susceptible	WT	WT	WT	susceptible	WT	WT	susceptible	N87T	WT	susceptible	WT
Hp_A-6	h110	North America	hpAfrica1	susceptible	WT	WT	WT	susceptible	WT	WT	susceptible	N87T	WT	susceptible	WT
Hp_A-8	h111	North America	hpAfrica1	susceptible	WT	WT	WT	susceptible	WT	WT	susceptible	N87T	WT	susceptible	WT
Hp_A-9	h112	North America	hpEurope	low level?	WT	WT	T2182C	susceptible	WT	WT	susceptible	WT	WT	susceptible	WT
Hp_H-1	h113	North America	hpAfrica1	unknown	unknown	unknown	unknown	susceptible	WT	WT	susceptible	N87T	WT	unknown	N/A
Hp_H-10	h114	North America	hpAfrica1	susceptible	WT	WT	WT	susceptible	WT	WT	susceptible	WT	WT	susceptible	WT
Hp_H-11	h115	North America	hpEurope	low level?	WT	WT	T2182C	resistant	WT	frxA-	susceptible	WT	WT	susceptible	WT
Hp_H-16	h116	North America	hpAfrica1	susceptible	WT	WT	WT	resistant	rdxA-	WT	susceptible	N87T	WT	susceptible	WT
Hp_H-18	h117	North America	hpAfrica1	susceptible	WT	WT	WT	resistant	WT	frxA-	susceptible	N87T	WT	susceptible	WT
Hp_H-19	h118	North America	hpAfrica1	susceptible	WT	WT	WT	resistant	WT	frxA-	susceptible	N87T	WT	susceptible	WT
Hp_H-21	h119	North America	hpAfrica1	susceptible	WT	WT	WT	susceptible	WT	WT	susceptible	N87T	WT	susceptible	WT
Hp_H-23	h120	North America	hpAfrica1	susceptible	WT	WT	WT	resistant	WT	frxA-	susceptible	N87T	WT	susceptible	WT
Hp_H-24	h121	North America	hpAfrica1	susceptible	WT	WT	WT	resistant	WT	frxA-	susceptible	N87T	WT	low level	AGC
Hp_H-24b	h122	North America	hpAfrica1	susceptible	WT	WT	WT	resistant	WT	frxA-	susceptible	N87T	WT	low level	AGC
Hp_H-24c	h123	North America	hpAfrica1	susceptible	WT	WT	WT	resistant	WT	frxA-	susceptible	N87T	WT	low level	AGC
Hp_H-27	h124	North America	hpEurope	susceptible	WT	WT	WT	susceptible	WT	WT	susceptible	WT	WT	susceptible	WT
Hp_H-28	h125	North America	hpEurope	susceptible	WT	WT	WT	susceptible	WT	WT	susceptible	WT	WT	unknown	N/A
Hp_H-29	h126	North America	hpAfrica1	susceptible	WT	WT	WT	susceptible	WT	WT	susceptible	N87T	WT	susceptible	WT
Hp_H-3	h127	North America	hpAfrica1	susceptible	WT	WT	WT	susceptible	WT	WT	resistant	N87I	WT	susceptible	WT
Hp_H-30	h128	North America	hpAfrica1	susceptible	WT	WT	WT	resistant	WT	frxA-	susceptible	N87T	WT	susceptible	WT
Hp_H-34	**h129**	North America	hpAfrica1	susceptible	WT	WT	WT	resistant	rdxA-	frxA-	susceptible	N87T	WT	susceptible	WT
Hp_H-36	h130	North America	hpAfrica1	susceptible	WT	WT	WT	resistant	WT	frxA-	susceptible	N87T	WT	susceptible	WT
Hp_H-4	h131	North America	hpAfrica1	susceptible	WT	WT	WT	susceptible	WT	WT	susceptible	N87T	WT	susceptible	WT
Hp_H-41	h132	North America	hpAfrica1	susceptible	WT	WT	WT	resistant	rdxA-	WT	susceptible	N87T	WT	susceptible	WT
Hp_H-42	h133	North America	hpAfrica1	susceptible	WT	WT	WT	susceptible	WT	WT	susceptible	N87T	WT	susceptible	WT
Hp_H-43	h134	North America	hpEurope	susceptible	WT	WT	WT	resistant	WT	frxA-	susceptible	WT	WT	susceptible	WT
Hp_H-44	h135	North America	hpAfrica1	unknown	unknown	unknown	unknown	susceptible	WT	WT	susceptible	N87T	WT	susceptible	WT
Hp_H-45	h136	North America	hpEurope	susceptible	WT	WT	WT	susceptible	WT	WT	susceptible	WT	WT	susceptible	WT
Hp_H-5b	h137	North America	hpAfrica1	susceptible	WT	WT	WT	susceptible	WT	WT	susceptible	N87T	WT	unknown	N/A
Hp_H-6	h138	North America	hpAfrica1	susceptible	WT	WT	WT	resistant	rdxA-	WT	susceptible	WT	WT	susceptible	WT
Hp_H-9	h139	North America	hpEurope	unknown	unknown	unknown	unknown	susceptible	WT	WT	susceptible	WT	WT	susceptible	WT
Hp_M1	h140	North America	hpAfrica1	unknown	unknown	unknown	unknown	resistant	WT	frxA-	susceptible	N87T	WT	low level	AGC
Hp_M2	h141	North America	hpAfrica1	susceptible	WT	WT	WT	resistant	WT	frxA-	susceptible	N87T	WT	low level	AGC
Hp_M3	h142	North America	hpAfrica1	susceptible	WT	WT	WT	resistant	WT	frxA-	susceptible	N87T	WT	low level	AGC
Hp_M4	h143	North America	hpAfrica1	susceptible	WT	WT	WT	resistant	WT	frxA-	susceptible	N87T	WT	low level	AGC
Hp_M5	h144	North America	hpAfrica1	susceptible	WT	WT	WT	resistant	WT	frxA-	susceptible	N87T	WT	low level	AGC
Hp_M6	h145	North America	hpAfrica1	susceptible	WT	WT	WT	resistant	WT	frxA-	susceptible	N87T	WT	low level	AGC
Hp_M9	**h146**	North America	hpAfrica1	susceptible	WT	WT	WT	resistant	WT	frxA-	susceptible	N87T	WT	low level	AGC
Hp_P-1	h147	North America	hpAfrica1	susceptible	WT	WT	WT	susceptible	WT	WT	susceptible	WT	WT	susceptible	WT
Hp_P-11	h148	North America	hpAfrica1	susceptible	WT	WT	WT	resistant	WT	frxA-	susceptible	N87T	WT	susceptible	WT
Hp_P-11b	h149	North America	hpAfrica1	susceptible	WT	WT	WT	resistant	WT	frxA-	susceptible	N87T	WT	susceptible	WT
Hp_P-13	h150	North America	hpAfrica1	susceptible	WT	WT	WT	susceptible	WT	WT	susceptible	WT	WT	susceptible	WT
Hp_P-13b	h151	North America	hpAfrica1	susceptible	WT	WT	WT	susceptible	WT	WT	susceptible	WT	WT	susceptible	WT
Hp_P-15	h152	North America	hpEurope	susceptible	WT	WT	WT	susceptible	WT	WT	susceptible	WT	WT	susceptible	WT
Hp_P-15b	h153	North America	hpEurope	susceptible	WT	WT	WT	susceptible	WT	WT	susceptible	WT	WT	susceptible	WT
Hp_P-16	h154	North America	hpEurope	susceptible	WT	WT	WT	susceptible	WT	WT	susceptible	WT	WT	susceptible	WT
Hp_P-1b	h155	North America	hpAfrica1	unknown	unknown	unknown	unknown	susceptible	WT	WT	susceptible	WT	WT	susceptible	WT
Hp_P-2	h156	North America	hpAfrica1	susceptible	WT	WT	WT	susceptible	WT	WT	susceptible	N87T	WT	susceptible	WT
Hp_P-23	h157	North America	hpEurope	unknown	unknown	unknown	unknown	susceptible	WT	WT	susceptible	WT	WT	susceptible	WT
Hp_P-25	h158	North America	hpAfrica1	susceptible	WT	WT	WT	resistant	rdxA-	frxA-	susceptible	N87T	WT	susceptible	WT
Hp_P-25c	h159	North America	hpAfrica1	susceptible	WT	WT	WT	resistant	rdxA-	frxA-	susceptible	N87T	WT	susceptible	WT
Hp_P-25d	h160	North America	hpAfrica1	susceptible	WT	WT	WT	resistant	rdxA-	frxA-	susceptible	N87T	WT	susceptible	WT

Hp_P-26	h161	North America	hpAfrica1	susceptible	WT	WT	WT	susceptible	WT	WT	susceptible	N87T	WT	susceptible	WT
Hp_P-28b	h162	North America	hpAfrica1	susceptible	WT	WT	WT	resistant	rdxA-	WT	susceptible	N87T	WT	susceptible	WT
Hp_P-2b	h163	North America	hpAfrica1	susceptible	WT	WT	WT	susceptible	WT	WT	susceptible	N87T	WT	susceptible	WT
Hp_P-3	h164	North America	hpAfrica1	susceptible	WT	WT	WT	susceptible	WT	WT	susceptible	N87T	WT	susceptible	WT
Hp_P-30	h165	North America	hpEurope	resistant	WT	A2143G	WT	susceptible	WT	WT	susceptible	WT	WT	susceptible	WT
Hp_P-3b	h166	North America	hpAfrica1	susceptible	WT	WT	WT	susceptible	WT	WT	susceptible	N87T	WT	susceptible	WT
Hp_P-4	h167	North America	hpAfrica1	susceptible	WT	WT	WT	susceptible	WT	WT	susceptible	N87T	WT	unknown	N/A
Hp_P-41	h168	North America	hpAfrica1	susceptible	WT	WT	WT	susceptible	WT	WT	susceptible	N87T	WT	susceptible	WT
Hp_P-4c	h169	North America	hpAfrica1	susceptible	WT	WT	WT	resistant	WT	frxA-	susceptible	N87T	WT	susceptible	WT
Hp_P-4d	h170	North America	hpAfrica1	unknown	unknown	unknown	unknown	susceptible	WT	WT	susceptible	N87T	WT	susceptible	WT
Hp_P-62	h171	North America	hpAfrica1	susceptible	WT	WT	WT	susceptible	WT	WT	susceptible	WT	WT	susceptible	WT
Hp_P-74	h172	North America	hpEurope	resistant	WT	A2143G	WT	susceptible	WT	WT	susceptible	WT	WT	susceptible	WT
Hp_P-8	h173	North America	hpAfrica1	susceptible	WT	WT	WT	unknown	WT	n/a	susceptible	N87T	WT	susceptible	WT
Hp_P-8b	h174	North America	hpAfrica1	susceptible	WT	WT	WT	unknown	WT	n/a	susceptible	N87T	WT	susceptible	WT
HP116Bi	**h175**	Africa	hpAfrica1	susceptible	WT	WT	WT	resistant	rdxA-	WT	susceptible	WT	WT	susceptible	GGA
HP250AFii	h176	Africa	hpAfrica1	susceptible	WT	WT	WT	resistant	WT	frxA-	susceptible	N87T	WT	low level	AGC
HP250AFiii	h177	Africa	hpAfrica1	susceptible	WT	WT	WT	resistant	WT	frxA-	susceptible	N87T	WT	low level	AGC
HP250AFiV	h178	Africa	hpAfrica1	susceptible	WT	WT	WT	resistant	WT	frxA-	susceptible	N87T	WT	low level	AGC
HP250ASi	h179	Africa	hpAfrica1	susceptible	WT	WT	WT	resistant	WT	frxA-	susceptible	N87T	WT	low level	AGC
HP250ASii	h180	Africa	hpAfrica1	susceptible	WT	WT	WT	resistant	rdxA-	frxA-	susceptible	N87T	WT	low level	AGC
HP250BFi	h181	Africa	hpAfrica1	susceptible	WT	WT	WT	resistant	WT	frxA-	susceptible	N87T	WT	low level	AGC
HP250BFii	h182	Africa	hpAfrica1	susceptible	WT	WT	WT	resistant	WT	frxA-	susceptible	N87T	WT	low level	AGC
HP250BFiii	h183	Africa	hpAfrica1	susceptible	WT	WT	WT	resistant	WT	frxA-	susceptible	N87T	WT	low level	AGC
HP250BFiV	h184	Africa	hpAfrica1	susceptible	WT	WT	WT	resistant	WT	frxA-	susceptible	N87T	WT	low level	AGC
HP250BSi	h185	Africa	hpAfrica1	susceptible	WT	WT	WT	resistant	rdxA-	frxA-	susceptible	N87T	WT	low level	AGC
HP260AFi	h186	Africa	hpAfrica1	susceptible	WT	WT	WT	resistant	WT	frxA-	susceptible	N87T	WT	susceptible	WT
HP260AFii	h187	Africa	hpAfrica1	susceptible	WT	WT	WT	resistant	WT	frxA-	susceptible	N87T	WT	susceptible	WT
HP260ASii	h188	Africa	hpAfrica1	susceptible	WT	WT	WT	resistant	rdxA-	frxA-	susceptible	N87T	WT	susceptible	WT
HP260BFii	h189	Africa	hpAfrica1	susceptible	WT	WT	WT	resistant	rdxA-	WT	susceptible	WT	WT	susceptible	WT
HP260Bi	h190	Africa	hpAfrica1	susceptible	WT	WT	WT	resistant	WT	frxA-	susceptible	N87T	WT	susceptible	WT
HP87hu	h191	Europe	hpEurope	low level?	WT	WT	T2182C	susceptible	WT	WT	susceptible	WT	WT	susceptible	WT
HP87P7	h192	Europe	hpEurope	susceptible	WT	WT	WT	susceptible	WT	WT	susceptible	WT	WT	susceptible	WT
HP87P7tlpDRI	h193	Europe	hpEurope	susceptible	WT	WT	WT	susceptible	WT	WT	susceptible	WT	WT	susceptible	WT
HP87tlpD	h194	Europe	hpEurope	susceptible	WT	WT	WT	susceptible	WT	WT	susceptible	WT	WT	susceptible	WT
HPAG1	**h195**	Europe	hpEurope	low level?	WT	WT	T2182C	resistant	WT	frxA-	susceptible	WT	WT	susceptible	WT
HPARG63	h196	South America	hpEurope	resistant	A2142G	WT	WT	resistant	WT	frxA-	susceptible	WT	WT	susceptible	WT
HPARG8G	h197	South America	hpEurope	resistant	A2142C	WT	WT	resistant	WT	frxA-	resistant	N87K	WT	susceptible	WT
HUP-B14	**h200**	Europe	hpEurope	susceptible	WT	WT	WT	susceptible	WT	WT	susceptible	N87T	WT	susceptible	WT
India7	**h201**	other (India)	hpAsia2	low level?	WT	WT	T2182C	resistant	rdxA-	WT	susceptible	WT	WT	susceptible	WT
J99	**h202**	North America	hpAfrica1	susceptible	WT	WT	WT	susceptible	WT	WT	susceptible	N87T	WT	susceptible	WT
Lithuania75	**h203**	Europe	hpEurope	susceptible	WT	WT	WT	susceptible	WT	WT	susceptible	WT	WT	susceptible	AGT
MALT	h204	North America	hpEurope	resistant	WT	A2143G	WT	resistant	rdxA-	frxA-	resistant	WT	D91N	susceptible	WT
N6	h205	Europe	hpEurope	low level?	WT	WT	T2182C	resistant	rdxA-	WT	susceptible	WT	WT	susceptible	WT
NAB47	**h206**	other (India)	hpAsia2	low level?	WT	WT	T2182C	susceptible	WT	WT	susceptible	WT	WT	susceptible	WT
NAD1	h207	other (India)	hpEurope	low level?	WT	WT	T2182C	resistant	rdxA-	n/a	resistant	N87I	WT	susceptible	WT
NAK7	h208	other (India)	hpAsia2	low level?	WT	WT	T2182C	susceptible	WT	WT	susceptible	WT	WT	susceptible	WT
NCTC_11637	h209	other (Australia)	hpEurope	susceptible	WT	WT	WT	susceptible	WT	WT	susceptible	WT	WT	susceptible	WT
NQ1671	h210	South America	hpEurope	susceptible	WT	WT	WT	resistant	WT	frxA-	susceptible	WT	WT	susceptible	WT
NQ1701	h211	South America	hpEurope	susceptible	WT	WT	WT	resistant	WT	frxA-	susceptible	WT	WT	susceptible	WT
NQ1707	h212	South America	hpEurope	susceptible	WT	WT	WT	resistant	WT	frxA-	susceptible	WT	WT	susceptible	WT
NQ1712	h213	South America	hpEurope	susceptible	WT	WT	WT	susceptible	WT	WT	susceptible	WT	WT	susceptible	WT
NQ315	h214	South America	hpEurope	susceptible	WT	WT	WT	resistant	WT	frxA-	susceptible	WT	WT	susceptible	WT
NQ352	h215	South America	hpEurope	susceptible	WT	WT	WT	resistant	rdxA-	frxA-	susceptible	WT	WT	susceptible	WT
NQ367	h216	South America	hpEurope	susceptible	WT	WT	WT	susceptible	WT	WT	susceptible	WT	WT	susceptible	WT
NQ392	h217	South America	hpEurope	susceptible	WT	WT	WT	resistant	WT	frxA-	susceptible	WT	WT	susceptible	WT
NQ4044	h218	South America	hpEurope	susceptible	WT	WT	WT	resistant	rdxA-	WT	susceptible	WT	WT	susceptible	GGA
NQ4053	h219	South America	hpEurope	susceptible	WT	WT	WT	susceptible	WT	WT	susceptible	WT	WT	susceptible	WT

NQ4060	h220	South America	hpEurope	susceptible	WT	WT	WT	resistant	rdxA-	frxA-	susceptible	WT	WT	susceptible	WT
NQ4076	h221	South America	hpEurope	susceptible	WT	WT	WT	susceptible	WT	WT	susceptible	WT	WT	susceptible	WT
NQ4099	h222	South America	hpEurope	susceptible	WT	WT	WT	resistant	WT	frxA-	resistant	WT	D91N	low level	AGC
NQ4110	h223	South America	hpEurope	susceptible	WT	WT	WT	resistant	WT	frxA-	susceptible	WT	WT	susceptible	WT
NQ4161	h224	South America	hpEurope	susceptible	WT	WT	WT	resistant	rdxA-	WT	susceptible	WT	WT	susceptible	WT
NQ4191	h225	South America	hpEurope	susceptible	WT	WT	WT	resistant	WT	frxA-	susceptible	WT	WT	susceptible	WT
NQ4200	h226	South America	hpEurope	susceptible	WT	WT	WT	resistant	WT	frxA-	susceptible	WT	WT	susceptible	WT
NQ4216	h227	South America	hpEurope	susceptible	WT	WT	WT	resistant	WT	frxA-	susceptible	WT	WT	susceptible	WT
NQ4228	h228	South America	hpEurope	susceptible	WT	WT	WT	resistant	WT	frxA-	susceptible	WT	WT	susceptible	WT
OK113	**h229**	East Asia	hspEAsia	low level?	WT	WT	T2182C	susceptible	WT	WT	susceptible	WT	WT	susceptible	WT
OK310	**h230**	East Asia	hspEAsia	low level?	WT	WT	T2182C	susceptible	WT	WT	susceptible	WT	WT	susceptible	WT
P12	**h231**	Europe	hpEurope	susceptible	WT	WT	WT	susceptible	WT	WT	susceptible	WT	WT	susceptible	WT
P79	h232	Europe	hpEurope	susceptible	WT	WT	WT	susceptible	WT	WT	susceptible	WT	WT	susceptible	WT
PeCan18	**h233**	South America	hpAfrica1	resistant	WT	A2143G	T2182C	resistant	rdxA-	frxA-	susceptible	WT	WT	susceptible	WT
PeCan4	**h234**	South America	hpEurope	susceptible	WT	WT	WT	resistant	WT	frxA-	resistant	WT	D91G	susceptible	WT
Puno120	**h235**	South America	hspAmerInd	low level?	WT	WT	T2182C	susceptible	WT	WT	resistant	WT	D91G	susceptible	WT
Puno135	**h236**	South America	hspAmerInd	low level?	WT	WT	T2182C	susceptible	WT	WT	susceptible	WT	WT	susceptible	WT
PZ5004	h237	South America	hpEurope	unknown	unknown	unknown	unknown	resistant	n/a	frxA-	susceptible	WT	WT	susceptible	WT
PZ5024	h238	South America	hpAfrica1	unknown	unknown	unknown	unknown	resistant	WT	frxA-	susceptible	N87T	WT	unknown	N/A
PZ5026	h239	South America	hpEurope	susceptible	WT	WT	WT	resistant	rdxA-	frxA-	susceptible	N87T	WT	susceptible	WT
PZ5056	h240	South America	hpEurope	susceptible	WT	WT	WT	resistant	rdxA-	frxA-	susceptible	WT	WT	unknown	N/A
PZ5080	h241	South America	hpEurope	unknown	unknown	unknown	unknown	resistant	WT	frxA-	susceptible	WT	WT	susceptible	WT
PZ5086	h242	South America	hpAfrica1	susceptible	WT	WT	WT	susceptible	WT	WT	susceptible	WT	WT	unknown	N/A
R018c	h243	North America	hpEurope	low level?	WT	WT	T2182C	susceptible	WT	WT	susceptible	WT	WT	unknown	N/A
R030b	**h244**	North America	hpAfrica1	susceptible	WT	WT	WT	susceptible	WT	WT	susceptible	N87T	WT	susceptible	WT
R036d	h245	North America	hpEurope	low level?	WT	WT	T2182C	susceptible	WT	WT	susceptible	WT	WT	susceptible	WT
R037c	h246	North America	hpEurope	susceptible	WT	WT	WT	resistant	rdxA-	WT	susceptible	WT	WT	susceptible	WT
R038b	h247	North America	hpEurope	susceptible	WT	WT	WT	susceptible	WT	WT	susceptible	WT	WT	unknown	N/A
R046Wa	h248	North America	hpEurope	susceptible	WT	WT	WT	susceptible	WT	WT	susceptible	WT	WT	susceptible	WT
R055a	h249	North America	hpEurope	low level?	WT	WT	T2182C	resistant	WT	frxA-	susceptible	WT	WT	unknown	N/A
R056a	h250	North America	hpEurope	low level?	WT	WT	T2182C	susceptible	WT	WT	susceptible	WT	WT	susceptible	WT
R32b	h251	North America	hpEurope	susceptible	WT	WT	WT	susceptible	WT	WT	susceptible	WT	WT	unknown	N/A
Rif1	h252	Europe	hpEurope	susceptible	WT	WT	WT	susceptible	WT	WT	susceptible	WT	WT	susceptible	WT
Rif2	h253	Europe	hpEurope	susceptible	WT	WT	WT	susceptible	WT	WT	susceptible	WT	WT	susceptible	WT
Sat464	**h254**	South America	hspAmerInd	low level?	WT	WT	T2182C	susceptible	WT	WT	susceptible	WT	WT	susceptible	WT
Shi112	**h255**	South America	hspAmerInd	low level?	WT	WT	T2182C	susceptible	WT	WT	susceptible	WT	WT	susceptible	WT
Shi169	**h256**	South America	hspAmerInd	low level?	WT	WT	T2182C	susceptible	WT	WT	susceptible	WT	WT	susceptible	WT
Shi417	**h257**	South America	hspAmerInd	low level?	WT	WT	T2182C	susceptible	WT	WT	susceptible	WT	WT	susceptible	WT
Shi470	**h258**	South America	hspAmerInd	low level?	WT	WT	T2182C	resistant	rdxA-	frxA-	susceptible	WT	WT	susceptible	WT
SJM180	**h259**	South America	hpEurope	susceptible	WT	WT	WT	susceptible	WT	WT	susceptible	WT	WT	susceptible	WT
SNT49	**h260**	other (India)	hpAsia2	low level?	WT	WT	T2182C	resistant	WT	frxA-	resistant	WT	D91N	susceptible	WT
SouthAfrica20	**h261**	Africa	hpAfrica2	susceptible	WT	WT	WT	resistant	rdxA-	WT	susceptible	N87S	WT	susceptible	WT
SouthAfrica50	h262	Africa	hpAfrica2	resistant	WT	A2143G	WT	resistant	rdxA-	frxA-	susceptible	N87R	WT	susceptible	WT
SouthAfrica7	**h263**	Africa	hpAfrica2	susceptible	WT	WT	WT	susceptible	WT	WT	susceptible	N87S	WT	susceptible	WT
UM007	h264	East Asia	hspEAsia	low level?	WT	WT	T2182C	resistant	rdxA-	WT	susceptible	WT	WT	susceptible	WT
UM018	h265	East Asia	hpAsia2	low level?	WT	WT	T2182C	resistant	WT	frxA-	susceptible	WT	WT	susceptible	WT
UM023	h266	East Asia	hspEAsia	low level?	WT	WT	T2182C	resistant	rdxA-	WT	susceptible	WT	WT	susceptible	WT
UM032	**h267**	East Asia	hspEAsia	low level?	WT	WT	T2182C	susceptible	WT	WT	susceptible	WT	WT	susceptible	WT
UM034	h268	East Asia	hspEAsia	low level?	WT	WT	T2182C	resistant	rdxA-	frxA-	susceptible	WT	WT	susceptible	WT
UM037	h269	East Asia	hpEurope	resistant	A2142G	WT	WT	resistant	WT	frxA-	susceptible	WT	WT	susceptible	WT
UM038	h270	East Asia	hspEAsia	resistant	WT	A2143G	T2182C	susceptible	WT	WT	resistant	WT	D91Y	susceptible	WT
UM045	h271	East Asia	hpEurope	susceptible	WT	WT	WT	resistant	rdxA-	frxA-	susceptible	WT	WT	susceptible	WT
UM054	**h272**	East Asia	hpAsia2	low level?	WT	WT	T2182C	susceptible	WT	WT	susceptible	WT	WT	susceptible	WT
UM065	h273	East Asia	hspEAsia	low level?	WT	WT	T2182C	susceptible	WT	WT	susceptible	WT	WT	susceptible	WT
UM066	h274	East Asia	hspEAsia	low level?	WT	WT	T2182C	susceptible	WT	WT	susceptible	WT	WT	susceptible	WT
UM067	h275	East Asia	hpAsia2	susceptible	WT	WT	WT	resistant	rdxA-	frxA-	susceptible	WT	WT	susceptible	WT
UM077	h276	East Asia	hspEAsia	low level?	WT	WT	T2182C	susceptible	WT	WT	resistant	N87K	WT	susceptible	WT

UM084	h277	East Asia	hpAsia2	low level?	WT	WT	T2182C	resistant	WT	frxA-	susceptible	WT	WT	susceptible	WT
UM085	h278	East Asia	hspEAsia	resistant	WT	A2143G	T2182C	susceptible	WT	WT	resistant	N87K	WT	susceptible	WT
UM111	h279	East Asia	hspEAsia	resistant	WT	A2143G	WT	resistant	WT	frxA-	susceptible	WT	WT	susceptible	WT
UM114	h280	East Asia	hpAsia2	low level?	WT	WT	T2182C	resistant	rdxA-	frxA-	resistant	N87K	WT	susceptible	WT
UM298	**h281**	East Asia	hspEAsia	low level?	WT	WT	T2182C	susceptible	WT	WT	susceptible	WT	WT	susceptible	WT
UM299	**h282**	East Asia	hspEAsia	low level?	WT	WT	T2182C	susceptible	WT	WT	susceptible	WT	WT	susceptible	WT
UMB_G1	h283	North America	hpEurope	resistant	WT	A2143G	WT	resistant	rdxA-	frxA-	resistant	WT	D91N	susceptible	WT
v225	**h284**	South America	hspAmerInd	low level?	WT	WT	T2182C	susceptible	WT	WT	susceptible	WT	WT	susceptible	WT
wls-5-12	h285	East Asia	hspEAsia	low level?	WT	WT	T2182C	resistant	rdxA-	WT	susceptible	WT	WT	susceptible	WT
wls-5-3	h286	East Asia	hspEAsia	low level?	WT	WT	T2182C	susceptible	WT	WT	susceptible	WT	WT	susceptible	WT
X47-2AL	h287	North America	hpEurope	susceptible	WT	WT	WT	susceptible	WT	WT	susceptible	WT	WT	susceptible	WT
XZ274	**h288**	East Asia	hspEAsia	low level?	WT	WT	T2182C	resistant	rdxA-	frxA-	susceptible	WT	WT	susceptible	WT
SA144A	h289	Africa	hpAfrica2	susceptible	WT	WT	WT	resistant	WT	frxA-	susceptible	N87S	WT	susceptible	WT
SA144C	h290	Africa	hpAfrica2	susceptible	WT	WT	WT	unknown	n/a	WT	susceptible	N87S	WT	low level	TGA
SA146A	h291	Africa	hpAfrica1	susceptible	WT	WT	WT	susceptible	WT	WT	susceptible	WT	WT	susceptible	GGA
SA146C	h292	Africa	hpAfrica1	susceptible	WT	WT	WT	susceptible	WT	WT	susceptible	WT	WT	susceptible	GGA
SA155A	h293	Africa	hpAfrica2	susceptible	WT	WT	WT	resistant	rdxA-	frxA-	susceptible	N87S	WT	susceptible	WT
SA155C	h294	Africa	hpAfrica2	susceptible	WT	WT	WT	resistant	rdxA-	WT	susceptible	N87S	WT	susceptible	WT
SA156A	h295	Africa	hpAfrica1	susceptible	WT	WT	WT	susceptible	WT	WT	susceptible	WT	WT	susceptible	WT
SA157A	h296	Africa	hpAfrica1	susceptible	WT	WT	WT	susceptible	WT	WT	susceptible	WT	WT	susceptible	WT
SA157C	h297	Africa	hpAfrica1	susceptible	WT	WT	WT	susceptible	WT	WT	susceptible	WT	WT	susceptible	WT
SA158A	**h298**	Africa	hpAfrica1	susceptible	WT	WT	WT	resistant	WT	frxA-	susceptible	WT	WT	susceptible	WT
SA158C	h299	Africa	hpAfrica1	susceptible	WT	WT	WT	susceptible	WT	WT	susceptible	WT	WT	susceptible	WT
SA160A	h300	Africa	hpAfrica2	susceptible	WT	WT	WT	susceptible	WT	WT	susceptible	N87S	WT	susceptible	WT
SA160C	h301	Africa	hpAfrica2	susceptible	WT	WT	WT	susceptible	WT	WT	susceptible	N87S	WT	susceptible	WT
SA161A	h302	Africa	hpAfrica1	susceptible	WT	WT	WT	susceptible	WT	WT	susceptible	N87S	WT	susceptible	WT
SA161C	h303	Africa	hpAfrica1	susceptible	WT	WT	WT	susceptible	WT	WT	susceptible	N87T	WT	susceptible	WT
SA162A	h304	Africa	hpAfrica1	susceptible	WT	WT	WT	susceptible	WT	WT	susceptible	N87T	WT	susceptible	WT
SA162C	h305	Africa	hpAfrica1	susceptible	WT	WT	WT	susceptible	WT	WT	susceptible	N87T	WT	susceptible	WT
SA163A	h306	Africa	hpAfrica1	susceptible	WT	WT	WT	susceptible	WT	WT	susceptible	WT	WT	susceptible	WT
SA163C	h307	Africa	hpAfrica1	susceptible	WT	WT	WT	susceptible	WT	WT	susceptible	WT	WT	susceptible	WT
SA164A	h308	Africa	hpEurope	susceptible	WT	WT	WT	resistant	WT	frxA-	susceptible	WT	WT	susceptible	WT
SA164C	h309	Africa	hpEurope	susceptible	WT	WT	WT	resistant	WT	frxA-	susceptible	WT	WT	susceptible	WT
SA165A	h310	Africa	hpEurope	susceptible	WT	WT	WT	resistant	WT	frxA-	susceptible	WT	WT	susceptible	WT
SA165C	h311	Africa	hpEurope	susceptible	WT	WT	WT	resistant	WT	frxA-	susceptible	WT	WT	susceptible	WT
SA166A	h312	Africa	hpAfrica2	susceptible	WT	WT	WT	resistant	WT	frxA-	susceptible	N87S	WT	susceptible	WT
SA168A	h313	Africa	hpAfrica1	susceptible	WT	WT	WT	resistant	rdxA-	frxA-	susceptible	N87S	WT	low level	TGA
SA168C	h314	Africa	hpAfrica1	susceptible	WT	WT	WT	susceptible	WT	WT	susceptible	N87S	WT	low level	TGA
SA169C	h315	Africa	hpAfrica2	resistant	WT	A2143G	WT	resistant	rdxA-	WT	susceptible	N87S	WT	susceptible	GGA
SA170A	h316	Africa	hpAfrica1	susceptible	WT	WT	WT	susceptible	WT	WT	susceptible	WT	WT	susceptible	WT
SA170C	h317	Africa	hpAfrica1	susceptible	WT	WT	WT	susceptible	WT	WT	susceptible	WT	WT	susceptible	WT
SA171A	h318	Africa	hpEurope	susceptible	WT	WT	WT	susceptible	WT	WT	susceptible	WT	WT	susceptible	WT
SA171C	h319	Africa	hpEurope	susceptible	WT	WT	WT	susceptible	WT	WT	susceptible	WT	WT	susceptible	WT
SA172A	h320	Africa	hpEurope	susceptible	WT	WT	WT	susceptible	WT	WT	susceptible	WT	WT	susceptible	WT
SA172C	h321	Africa	hpAfrica2	susceptible	WT	WT	WT	resistant	WT	frxA-	susceptible	N87S	WT	susceptible	WT
SA173A	h322	Africa	hpEurope	susceptible	WT	WT	WT	susceptible	WT	WT	susceptible	WT	WT	susceptible	WT
SA173C	h323	Africa	hpEurope	susceptible	WT	WT	WT	susceptible	WT	WT	susceptible	WT	WT	susceptible	WT
SA174A	h324	Africa	hpAfrica2	susceptible	WT	WT	WT	susceptible	WT	WT	susceptible	N87S	WT	susceptible	WT
SA175A	h325	Africa	hpAfrica2	susceptible	WT	WT	WT	susceptible	WT	WT	susceptible	N87T	WT	susceptible	WT
SA175C	h326	Africa	hpAfrica2	susceptible	WT	WT	WT	susceptible	WT	WT	susceptible	N87T	WT	susceptible	WT
SA194A	h327	Africa	hpAfrica2	susceptible	WT	WT	WT	susceptible	WT	WT	susceptible	N87S	WT	susceptible	WT
SA194C	h328	Africa	hpAfrica2	susceptible	WT	WT	WT	unknown	n/a	n/a	susceptible	N87S	WT	susceptible	WT
SA210A	**h329**	Africa	hpAfrica1	susceptible	WT	WT	WT	susceptible	WT	WT	susceptible	WT	WT	susceptible	WT
SA210C	h330	Africa	hpAfrica1	susceptible	WT	WT	WT	susceptible	WT	WT	susceptible	WT	WT	susceptible	WT
SA213A	h331	Africa	hpEurope	susceptible	WT	WT	WT	resistant	rdxA-	frxA-	susceptible	WT	WT	susceptible	WT
SA214A	**h332**	Africa	hpAfrica1	susceptible	WT	WT	WT	susceptible	WT	WT	susceptible	WT	WT	susceptible	WT
SA214C	h333	Africa	hpAfrica1	susceptible	WT	WT	WT	susceptible	WT	WT	susceptible	WT	WT	susceptible	WT

SA215C	h334	Africa	hpAfrica1	susceptible	WT	WT	WT	susceptible	WT	WT	susceptible	WT	WT	susceptible	WT
SA216C	h335	Africa	hpAfrica1	susceptible	WT	WT	WT	susceptible	WT	WT	susceptible	WT	WT	susceptible	WT
SA220A	h336	Africa	hpAfrica1	susceptible	WT	WT	WT	resistant	WT	frxA-	susceptible	WT	WT	susceptible	WT
SA220C	h337	Africa	hpAfrica1	susceptible	WT	WT	WT	resistant	WT	frxA-	susceptible	WT	WT	susceptible	WT
SA221A	h338	Africa	hpEurope	susceptible	WT	WT	WT	unknown	n/a	WT	susceptible	WT	WT	susceptible	WT
SA221C	h339	Africa	hpEurope	susceptible	WT	WT	WT	unknown	n/a	n/a	susceptible	WT	WT	susceptible	WT
SA222A	h340	Africa	hpAsia2	low level?	WT	WT	T2182C	resistant	rdxA-	frxA-	susceptible	WT	WT	susceptible	WT
SA222C	**h341**	Africa	hpAsia2	low level?	WT	WT	T2182C	resistant	rdxA-	WT	susceptible	WT	WT	susceptible	WT
SA224A	h342	Africa	hpAfrica1	susceptible	WT	WT	WT	resistant	rdxA-	frxA-	susceptible	N87S	WT	susceptible	WT
SA224C	h343	Africa	hpAfrica1	susceptible	WT	WT	WT	resistant	rdxA-	frxA-	susceptible	N87S	WT	susceptible	GGA
SA226A	h344	Africa	hpAfrica1	susceptible	WT	WT	WT	susceptible	WT	WT	susceptible	WT	WT	susceptible	WT
SA227A	h345	Africa	hpAfrica1	susceptible	WT	WT	WT	resistant	rdxA-	WT	susceptible	WT	WT	susceptible	WT
SA233A	h346	Africa	hpAfrica2	susceptible	WT	WT	WT	susceptible	WT	WT	susceptible	N87S	WT	susceptible	WT
SA233C	h347	Africa	hpAfrica2	susceptible	WT	WT	WT	susceptible	WT	WT	susceptible	N87S	WT	susceptible	WT
SA251A	h348	Africa	hpAfrica2	susceptible	WT	WT	WT	unknown	n/a	n/a	susceptible	N87S	WT	susceptible	WT
SA251C	h349	Africa	hpAfrica2	susceptible	WT	WT	WT	susceptible	WT	WT	susceptible	N87S	WT	susceptible	WT
SA252A	h350	Africa	hpAfrica1	susceptible	WT	WT	WT	susceptible	WT	WT	susceptible	N87T	WT	low level	TGA
SA252C	h351	Africa	hpAfrica1	susceptible	WT	WT	WT	resistant	WT	frxA-	susceptible	N87S	WT	low level	TGA
SA253A	h352	Africa	hpAfrica2	susceptible	WT	WT	WT	unknown	n/a	n/a	susceptible	N87S	WT	susceptible	WT
SA253C	h353	Africa	hpAfrica2	susceptible	WT	WT	WT	resistant	rdxA-	WT	susceptible	N87S	WT	susceptible	WT
SA29A	h354	Africa	hpAfrica2	susceptible	WT	WT	WT	unknown	n/a	WT	susceptible	N87S	WT	susceptible	WT
SA29C	h355	Africa	hpAfrica1	susceptible	WT	WT	WT	susceptible	WT	WT	susceptible	N87S	WT	susceptible	GGA
SA300A	h356	Africa	hpAfrica1	susceptible	WT	WT	WT	susceptible	WT	WT	susceptible	WT	WT	susceptible	WT
SA300C	h357	Africa	hpAfrica1	susceptible	WT	WT	WT	susceptible	WT	WT	susceptible	WT	WT	susceptible	WT
SA301A	h358	Africa	hpAfrica1	susceptible	WT	WT	WT	resistant	WT	frxA-	susceptible	N87T	WT	susceptible	WT
SA301C	h359	Africa	hpAfrica1	susceptible	WT	WT	WT	resistant	rdxA-	frxA-	susceptible	N87T	WT	susceptible	WT
SA302A	h360	Africa	hpEurope	resistant	WT	A2143G	WT	resistant	rdxA-	frxA-	susceptible	WT	WT	susceptible	WT
SA302C	h361	Africa	hpEurope	resistant	WT	A2143G	WT	resistant	rdxA-	frxA-	susceptible	WT	WT	susceptible	WT
SA303C	h362	Africa	hpAfrica2	susceptible	WT	WT	WT	susceptible	WT	WT	susceptible	N87S	WT	susceptible	GGA
SA30A	h363	Africa	hpAfrica1	susceptible	WT	WT	WT	susceptible	WT	WT	susceptible	WT	WT	susceptible	WT
SA30C	h364	Africa	hpAfrica1	susceptible	WT	WT	WT	susceptible	WT	WT	susceptible	WT	WT	susceptible	WT
SA31C	h365	Africa	hpAfrica1	susceptible	WT	WT	WT	resistant	rdxA-	WT	susceptible	WT	WT	susceptible	WT
SA34A	h366	Africa	hpAfrica2	susceptible	WT	WT	WT	resistant	rdxA-	WT	susceptible	N87S	WT	susceptible	WT
SA34C	h367	Africa	hpAfrica2	susceptible	WT	WT	WT	susceptible	WT	WT	susceptible	N87S	WT	susceptible	WT
SA35A	h368	Africa	hpAfrica1	susceptible	WT	WT	WT	resistant	rdxA-	WT	susceptible	WT	WT	susceptible	WT
SA36C	h369	Africa	hpAfrica2	susceptible	WT	WT	WT	resistant	rdxA-	WT	susceptible	N87S	WT	susceptible	WT
SA37A	h370	Africa	hpEurope	susceptible	WT	WT	WT	susceptible	WT	WT	susceptible	WT	WT	susceptible	WT
SA37C	h371	Africa	hpEurope	susceptible	WT	WT	WT	susceptible	WT	WT	susceptible	WT	WT	susceptible	WT
SA40A	h372	Africa	hpAfrica2	susceptible	WT	WT	WT	resistant	WT	frxA-	susceptible	N87S	WT	susceptible	WT
SA40C	h373	Africa	hpAfrica2	susceptible	WT	WT	WT	resistant	WT	frxA-	susceptible	N87S	WT	susceptible	WT
SA45A	h374	Africa	hpAfrica1	susceptible	WT	WT	WT	susceptible	WT	WT	susceptible	WT	WT	susceptible	WT
SA45C	h375	Africa	hpAfrica1	susceptible	WT	WT	WT	susceptible	WT	WT	susceptible	N87T	WT	susceptible	WT
SA46C	h376	Africa	hpAfrica1	susceptible	WT	WT	WT	resistant	rdxA-	WT	susceptible	WT	WT	susceptible	WT
SA47A	h377	Africa	hpAfrica2	susceptible	WT	WT	WT	susceptible	WT	WT	susceptible	N87S	WT	susceptible	WT
SA47C	h378	Africa	hpAfrica2	susceptible	WT	WT	WT	susceptible	WT	WT	susceptible	N87S	WT	susceptible	WT
Sahul64	h379	other (Australia)	hpSahul	susceptible	WT	WT	WT	resistant	rdxA-	WT	susceptible	WT	WT	susceptible	WT

References

1. **Sims GE, Jun SR, Wu GA, Kim SH.** 2009. Alignment-free genome comparison with feature frequency profiles (FFP) and optimal resolutions. Proc Natl Acad Sci U S A **106:**2677-2682.
2. **Zerbino DR, Birney E.** 2008. Velvet: algorithms for de novo short read assembly using de Bruijn graphs. Genome Res **18:**821-829.
3. **Didelot X, Nell S, Yang I, Woltemate S, van der Merwe S, Suerbaum S.** 2013. Genomic evolution and transmission of Helicobacter pylori in two South African families. Proc Natl Acad Sci U S A **110:**13880-13885.
4. **Tamura K, Peterson D, Peterson N, Stecher G, Nei M, Kumar S.** 2011. MEGA5: molecular evolutionary genetics analysis using maximum likelihood, evolutionary distance, and maximum parsimony methods. Mol Biol Evol **28:**2731-2739.
5. **Falush D, Kraft C, Taylor NS, Correa P, Fox JG, Achtman M, Suerbaum S.** 2001. Recombination and mutation during long-term gastric colonization by Helicobacter pylori: estimates of clock rates, recombination size, and minimal age. Proc Natl Acad Sci U S A **98:**15056-15061.
6. **Moodley Y, Linz B, Bond RP, Nieuwoudt M, Soodyall H, Schlebusch CM, Bernhoft S, Hale J, Suerbaum S, Mugisha L, van der Merwe SW, Achtman M.** 2012. Age of the association between Helicobacter pylori and man. PLoS Pathog **8:**e1002693.
7. **Abadi AT, Taghvaei T, Wolfram L, Kusters JG.** 2012. Infection with Helicobacter pylori strains lacking dupA is associated with an increased risk of gastric ulcer and gastric cancer development. J Med Microbiol **61:**23-30.
8. **Maeda S, Yoshida H, Ikenoue T, Ogura K, Kanai F, Kato N, Shiratori Y, Omata M.** 1999. Structure of cag pathogenicity island in Japanese Helicobacter pylori isolates. Gut **44:**336-341.
9. **Tummuru MK, Cover TL, Blaser MJ.** 1993. Cloning and expression of a high-molecular-mass major antigen of Helicobacter pylori: evidence of linkage to cytotoxin production. Infect Immun **61:**1799-1809.
10. **Armitano RI, Matteo MJ, Goldman C, Wonaga A, Viola LA, De Palma GZ, Catalano M.** 2013. Helicobacter pylori heterogeneity in patients with gastritis and peptic ulcer disease. Infect Genet Evol **16:**377-385.

11. **Atherton JC, Cao P, Peek RM, Jr., Tummuru MK, Blaser MJ, Cover TL.** 1995. Mosaicism in vacuolating cytotoxin alleles of Helicobacter pylori. Association of specific vacA types with cytotoxin production and peptic ulceration. J Biol Chem **270:**17771-17777.
12. **Atherton JC, Cover TL, Twells RJ, Morales MR, Hawkey CJ, Blaser MJ.** 1999. Simple and accurate PCR-based system for typing vacuolating cytotoxin alleles of Helicobacter pylori. J Clin Microbiol **37:**2979-2982.
13. **Rhead JL, Letley DP, Mohammadi M, Hussein N, Mohagheghi MA, Eshagh Hosseini M, Atherton JC.** 2007. A new Helicobacter pylori vacuolating cytotoxin determinant, the intermediate region, is associated with gastric cancer. Gastroenterology **133:**926-936.
14. **Ogiwara H, Sugimoto M, Ohno T, Vilaichone RK, Mahachai V, Graham DY, Yamaoka Y.** 2009. Role of deletion located between the intermediate and middle regions of the Helicobacter pylori vacA gene in cases of gastroduodenal diseases. J Clin Microbiol **47:**3493-3500.
15. **Mizushima T, Sugiyama T, Komatsu Y, Ishizuka J, Kato M, Asaka M.** 2001. Clinical relevance of the babA2 genotype of Helicobacter pylori in Japanese clinical isolates. J Clin Microbiol **39:**2463-2465.
16. **Takahashi A, Shiota S, Matsunari O, Watada M, Suzuki R, Nakachi S, Kinjo N, Kinjo F, Yamaoka Y.** 2013. Intact long-type dupA as a marker for gastroduodenal diseases in Okinawan subpopulation, Japan. Helicobacter **18:**66-72.
17. **van Doorn LJ, Figueiredo C, Sanna R, Plaisier A, Schneeberger P, de Boer W, Quint W.** 1998. Clinical relevance of the cagA, vacA, and iceA status of Helicobacter pylori. Gastroenterology **115:**58-66.
